# Efficacy, safety and genomic analysis of SCT200, an anti-EGFR monoclonal antibody, in patients with fluorouracil, irinotecan and oxaliplatin refractory *RAS* and *BRAF* wild-type metastatic colorectal cancer: a phase Ⅱ study

**DOI:** 10.1016/j.ebiom.2024.104966

**Published:** 2024-01-13

**Authors:** Lin Yang, Wen Zhang, Nanfeng Fan, Peiguo Cao, Ying Cheng, Lingjun Zhu, Suxia Luo, Hong Zong, Yuxian Bai, Jianfeng Zhou, Yanhong Deng, Yi Ba, Tianshu Liu, Mayinuer Aili, Xianli Yin, Kangsheng Gu, Guanghai Dai, Jieer Ying, Jianhua Shi, Yajie Gao, Wei Li, Guohua Yu, Liangzhi Xie, Wenlin Gai, Yan Wang, Peng Meng, Yuankai Shi

**Affiliations:** a Department of Medical Oncology, National Cancer Center/National Clinical Research Center for Cancer/Cancer Hospital, Chinese Academy of Medical Sciences & Peking Union Medical College, Beijing Key Laboratory of Clinical Study on Anticancer Molecular Targeted Drugs, Beijing, China; b Department of Abdominal Oncology, Fujian Provincial Cancer Hospital, Fuzhou, China; c Department of Oncology, The Third Xiangya Hospital of Central South University, Changsha, China; d Department of Oncology, Cancer Hospital of Jilin Province, Changchun, China; e Department of Oncology, Jiangsu Province Hospital, Nanjing, China; f Department of Medical Oncology, Henan Provincial Cancer Hospital, Zhengzhou, China; g Department of Oncology, The First Affiliated Hospital of Zhengzhou University, Zhengzhou, China; h Department of Internal Medicine, Harbin Medical University Cancer Hospital, Harbin, China; i Department of Medical Oncology, Peking Union Medical College Hospital, Chinese Academy of Medical Sciences & Peking Union Medical College, Beijing, China; j Department of Medical Oncology, The Sixth Affiliated Hospital, Sun Yat-Sen University, Guangzhou, China; k Department of Gastroenterology, Tianjin Medical University Cancer Institute & Hospital, Tianjin, China; l Department of Medical Oncology, Zhongshan Hospital Affiliated to Fudan University, Shanghai, China; m The Third Department of Oncology, Cancer Center, The First Affiliated Hospital of Xinjiang Medical University, Urumqi, China; n Department of Gastroenterology, Hunan Cancer Hospital, Changsha, China; o Department of Medical Oncology, The First Affiliated Hospital of Anhui Medical University, Hefei, China; p Department of Medical Oncology, Chinese PLA General Hospital, Beijing, China; q Department of Abdominal Oncology, Zhejiang Cancer Hospital, Hangzhou, China; r Department of Medical Oncology, Linyi Cancer Hospital, Linyi, China; s Department of Oncology, The First Affiliated Hospital of Dalian Medical University, Dalian, China; t Cancer Center, The First Hospital of Jilin University, Changchun, China; u Department of Oncology, Weifang People's Hospital, Weifang, China; v Beijing Engineering Research Center of Protein and Antibody, Sinocelltech Ltd., Beijing, China; w Burning Rock Biotech, Shanghai, China

**Keywords:** Anti-epidermal growth factor receptor, Monoclonal antibody, Metastatic colorectal cancer, *RAS*, *BRAF*, Circulating tumor DNA

## Abstract

**Background:**

Limited therapeutic options are available for metastatic colorectal cancer (mCRC) patients after failure of first- and second-line therapies, representing an unmet medical need for novel therapies.

**Methods:**

This is an open-label, single arm, multicenter, phase Ⅱ study aiming to perform the efficacy, safety and genomic analysis of SCT200, a noval fully humanized IgG1 anti-epidermal growth factor receptor (EGFR) monoclonal antibody, in patients with fluorouracil, irinotecan and oxaliplatin refractory *RAS* and *BRAF* wild-type mCRC. SCT200 (6 mg/kg) was given weekly for the first six weeks, followed by a higher dose of 8 mg/kg every two weeks until disease progression or unacceptable toxicity. Primary endpoint was independent review committee (IRC)-assessed objective response rate (ORR) and secondary endpoints included ORR in patients with left-sided tumor, disease control rate (DCR), duration of response (DoR), time to response (TTR), progression-free survival (PFS), overall survival (OS) and safety.

**Findings:**

From February 12, 2018 to December 1, 2019, a total of 110 patients aged between 26 and 77 years (median: 55; interquartile range [IQR]: 47–63) with fluorouracil, oxaliplatin, and irinotecan refractory *RAS* and *BRAF* wild-type mCRC were enrolled from 22 hospitals in China. As the data cut-off date on May 15, 2020, the IRC-assessed ORR and DCR was 31% (34/110, 95% confidence interval [CI] 22–40%) and 75% (82/110, 95% CI 65–82%), respectively. Thirty one percent (34/110) patients achieved confirmed partial response (PR). The median PFS and median OS were 5.1 months (95% CI 3.4–5.2) and 16.2 months (95% CI 11.1-not available [NA]), respectively. The most common ≥ grade 3 treatment-related adverse events (TRAEs) were hypomagnesemia (17%, 19/110) and acneiform dermatitis (11%, 12/110). No deaths occurred. Genomic analysis suggested positive association between *MYC* amplification and patients’ response (*P* = 0.0058). *RAS/RAF* mutation and *MET* amplification were the most frequently detected resistance mechanisms. Patients with high circulating tumor DNA (ctDNA) at baseline or without ctDNA clearance at the 7th week after the first dose of SCT200 administration before receiving SCT200 had worse PFS and OS.

**Interpretation:**

SCT200 exhibited promising clinical efficacy and manageable safety profiles in *RAS* and *BRAF* wild-type mCRC patients progressed on fluorouracil, irinotecan and oxaliplatin treatment. The baseline ctDNA and ctDNA clearance status at the 7th week after the first dose of SCT200 administration before receiving SCT200 could be a potential prognostic biomarker for *RAS* and *BRAF* wild-type mCRC patients with SCT200 therapy.

**Funding:**

This study was sponsored by Sinocelltech Ltd., Beijing, China and partly supported by the National Science and Technology Major Project for Key New Drug Development (2019ZX09732001-006, 2017ZX09304015).

**Research in context**

**Evidence before this study**

Few therapeutic options are available for metastatic colorectal cancer (mCRC) patients after failure of first- and second-line therapies. SCT200 is a novel fully humanized IgG1 anti-epidermal growth factor receptor (EGFR) monoclonal antibody (mAb), with distinguished antigen-binding epitope, physicochemical properties, and biological activity from other available mAbs like cetuximab or panitumumab in the market. The phase I study results showed manageable safety profile and favorable efficacy in fluorouracil, irinotecan and oxaliplatin refractory patients with *KRAS*/*NRAS*/*BRAF* wild-type mCRC. Therefore, we conducted this open-label, single arm, multicenter, phase Ⅱ study aiming to perform the efficacy, safety and genomic analysis of SCT200 in these patients population.

**Added value of this study**

In this open-label, single arm, multicenter, phase Ⅱ study, the objective response rate (ORR) was 31% (34/110, 95% confidence interval [CI] 22–40%) with a median progression-free survival (PFS) of 5.1 months (95% CI 3.4–5.2) and median overall survival (OS) of 16.2 months (95% CI 11.1–not available [NA]), and SCT200 was well tolerated. Predictive and prognostic biomarkers were analyzed with baseline paired tumor tissue and plasma samples. Positive association between *MYC* amplification and patients’ response (*P* = 0.0058) were found. *RAS*/*BRAF* mutation and *MET* amplification were the most frequently detected resistance mechanisms. Compared with traditional radiological assessment tools, baseline circulating tumor DNA (ctDNA) levels and ctDNA clearance status at the 7th week after the first dose of SCT200 administration before receiving SCT200 were better prognostic tools to stratify patients with worse PFS and OS.

**Implications of all the available evidence**

This study highlights the efficacy and safety of SCT200 in patients with fluorouracil, irinotecan and oxaliplatin refractory *RAS* and *BRAF* wild-type metastatic colorectal cancer, indicating that this regimen could be a promising option as a single agent for such patients. Biomarkers for response were identified, and the prognostic value of circulating tumor DNA level at baseline and first visit was clarified. While further studies are needed to validate these findings.

## Introduction

Colorectal cancer (CRC), the world's third most common cancer, contributes to about 10% of global cancer burden and affects 1,065,960 men and 865,630 women.^1^ Chemotherapies such as fluorouracil, folinic acid combined with oxaliplatin (FOLFOX) or folinic acid combined with irinotecan (FOLFIRI) regimens have shown benefits by improving survival for patients with metastatic CRC (mCRC).^2^

The epidermal growth factor receptor (EGFR) is overexpressed in 30–90% patients with mCRC,^3^ implying treatment targeting EGFR could be a valid strategy. The development of anti-EGFR monoclonal antibody (mAb) has refined the treatment of chemotherapy-refractory mCRC since almost two decades ago. In 2004, the United States Food and Drug Administration approved antibody based targeted therapies, bevacizumab and cetuximab.^4^^,^^5^ In addition to cetuximab, panitumumab was also approved to target against EGFR for the treatment of mCRC.^6^ In China, cetuximab has been approved for the first-line use in patients with *RAS* wild-type mCRC on September 18, 2019 and for the first-line treatment of patients with recurrent and/or metastatic squamous cell carcinoma of the head and neck (R/M SCCHN) on March 2, 2020. Another anti-EGFR mAb, nimotuzumab, was approved in China for the treatment of nasopharyngeal cancer (NPC) on January 7, 2008.^7^

SCT200, developed by Sinocelltech Ltd., Beijing, China, is a recombinant, fully humanized IgG1 anti-EGFR mAb, which is different from the human-mouse chimeric IgG1 anti-EGFR mAb, cetuximab, or the fully humanized IgG2 anti-EGFR mAb, panitumumab. Preclinical studies have shown a higher affinity of SCT200 (Kd = 0.08 nM) than that of cetuximab (Kd = 0.147 nM) to EGFR. Meanwhile, as an IgG1 mAb with higher FcγR-binding affinity,^8^ SCT200 had a superior antibody-dependent cellular cytotoxicity (ADCC) activity to panitumumab. SCT200 can kill tumor cells by complement-dependent cytotoxicity (CDC) and ADCC through the Fc, with a cytotoxicity of over 30%. Compared to cetuximab, the immunogenicity of SCT200 was greatly reduced, which might be more suitable for long-term administration for patients with malignant tumors. The results of the phase I study of SCT200 showed manageable safety profile and favorable efficacy in fluorouracil, irinotecan and oxaliplatin refractory patients with *KRAS*/*NRAS*/*BRAF* wild-type mCRC.^9^

Few therapeutic options are available after failure of first- and second-line therapies. The standard of care (SOC) in this setting is represented by regorafenib, fruquintinib, trifluoridine/tipiracil hydro-chloride (TAS-102), anti-EGFR mAbs for patients with *RAS* wild-type tumors (if no prior exposure), or anti-programmed cell death protein 1 (PD-1) mAbs for patients with microsatellite instability-high (MSI-H) mCRC.^10^, ^11^, ^12^ Various biomarkers, like *RAS*, *BRAF*, and MSI, can be used to guide further stratification for third or posterior line treatments.

CRC is also known for its spatial and temporal intra-tumor heterogeneity. Growing evidences supports the application of circulating tumor DNA (ctDNA) in detecting residual disease, diagnosing recurrence, enabling targeted therapies, and assessing treatment response as well as indicating development of resistance to therapy.^13^

In this open-label, single arm, multicenter, phase Ⅱ study, we performed the efficacy, safety and genomic analysis of SCT200 in patients with fluorouracil, irinotecan and oxaliplatin refractory *RAS* and *BRAF* wild-type mCRC. Targeted sequencing was performed in formalin-fixed paraffin-embedded (FFPE) tumor tissue samples at a minimum interval of four weeks before the first dose of SCT200 administration to exclude the ineligible patients and analyzed the genomic profiles. Genomic analysis was also applied to depict the baseline genomic profiles and dynamically monitor treatment response and emergence of resistance.

## Methods

### Study design and treatment

This is an open-label, single arm, multicenter, phase Ⅱ study aiming to perform the efficacy, safety and genomic analysis of SCT200, a novel fully humanized IgG1 anti-EGFR mAb, in patients with fluorouracil, irinotecan and oxaliplatin refractory *RAS* and *BRAF* wild-type mCRC.

The patients inclusion criteria were as follows: age of 18 years or older; life expectancy of at least three months; Eastern Cooperative Oncology Group (ECOG) performance status (PS) score of 0 or 1; histologically confirmed chemo-refractory mCRC who had undergone disease progression or unacceptable toxicity during or within six months from the last dose of SOC chemotherapies containing fluorouracil, oxaliplatin, or irinotecan; *RAS* and *BRAF* wild-type status in tissue samples assessed by next-generation sequencing (NGS) based mutation profiling; adequate organ and bone marrow function as defined; at least one measurable lesion according to the Response Evaluation Criteria in Solid Tumors (RECIST) version 1.1. A complete list of the inclusion and exclusion criteria are defined in the protocol on line (https://clinicaltrials.gov/ct2/show/NCT03405272). In strict accordance with the guidelines for single arm study design, a superiority test for the objective response rate (ORR) was performed.

SCT200 6 mg/kg was administered once a week for the first six weeks, followed by SCT200 8 mg/kg every two weeks. Treatment was continued until disease progression or unacceptable toxicity. This dosing schedule was designed primarily on the basis of SCT200 phase I study.^9^

### Ethics

The study protocols were approved by the ethics committee of all the participating hospitals in accordance with the Declaration of Helsinki and its amendments. Written informed consent of every patient was obtained before enrollment. The clinical trial registration number was NCT03405272.

### Clinical outcomes

The primary endpoint of this study was independent review committee (IRC)-assessed ORR according to the RECIST version 1.1. The secondary endpoints were IRC-assessed ORR in patients with left-sided tumor, IRC-assessed disease control rate (DCR), duration of response (DoR), time to response (TTR), progression-free survival (PFS), overall survival (OS) and safety. The efficacy analyses were performed in the full analysis set (FAS) defined as those patients who received at least one dose of SCT200. For the safety assessment, the safety set (SS) included all the patients received at least one dose of SCT200 and had safety assessments after treatment. Safety referred to treatment-emergent adverse events (TEAEs), treatment-related adverse events (TRAEs), serious adverse events (SAEs), fatal adverse events (FAEs) and adverse event of special interest (AESI) were evaluated according to the National Cancer Institute Common Terminology Criteria for Adverse Events (CTCAE) version 4.03.

ORR was defined as the percentage of complete response (CR) or partial response (PR). DCR was defined as the percentage of patients with CR or PR or stable disease (SD). PFS was defined as the time from the first dose of SCT200 to the date of progressive disease (PD) or death of any reason. OS was defined as the time from the first dose of SCT200 to the date of death due to any reason or last follow-up.

### Genomic analysis

FFPE tumor tissue sample collection was undertaken for all enrolled patients with a minimum interval of four weeks before the first dose of SCT200 administration. For further exploratory study, dynamic blood plasma samples collected at the baseline within seven days before first dose of SCT200 administration, the 7th week after the first dose of SCT200 administration before receiving SCT200 followed by every eight weeks ± seven days, and at the time of disease progression. Samples of tumor tissue and paired blood for genomic analysis were transported to a designated central laboratory (Burning Rock Biotech, Shanghai, China) for mutation testing. Tissue DNA and ctDNA were isolated from FFPE tumor tissue and paired blood plasma samples, respectively. DNA extraction, targeted capture and sequencing were performed as previously described.^14^^,^^15^

*RAS/BRAF* alteration status was assessed by NGS using a targeted panel of 108 genes (Burning Rock Dx.) for eligibility determination. Additional somatic mutations from genomic DNA and ctDNA were analyzed by the same panel for potential biomarkers that might affect the efficacy or safety of SCT200 treatment, including alterations in *PIK3CA*, *EGFR* and *TP53*.

### Statistical analysis

The sample size of this study was estimated based on the primary endpoint ORR. An ORR of ≤20% was considered to be of little clinical efficacy (null hypothesis), and an ORR of >20% was considered responsive treatment (alternative hypothesis). Considering a 10% dropout rate, 110 patients were planned to accrue in this study, providing over 90% power with a type I error of 0.05 to reject the null hypothesis.

All statistical analyses were performed in R software version 3.3.3 (Vienna, Austria), using two-sided tests, unless otherwise specified. The quantitative parameters were described as the number and percentage of cases with the categorical variables described as median with interquartile range (IQR) and range. Differences between two groups were analyzed by Fisher's exact test for categorical variables. Point estimates and 95% confidence intervals (CIs) for ORR or DCR were generated using exact binomial distribution using the Clopper-Pearson method. DoR, PFS, and OS were summarized descriptively using Kaplan–Meier estimates, and their 95% CIs were estimated by Brookmeyer-Crowley method. *P* < 0.05 were defined as statistically significant for all analyses.

### Role of funders

The sponsor of the study was Sinocelltech Ltd. The trial was financed by Sinocelltech Ltd. and partly supported by the National Science and Technology Major Project for Key New Drug Development (2019ZX09732001-006, 2017ZX09304015). Study design, data collection, analysis, interpretation and writing of the report, as well as the decision to submit the paper for publication were done solely by the authors.

## Results

### Patient baseline characteristics

**Fig. 1:**
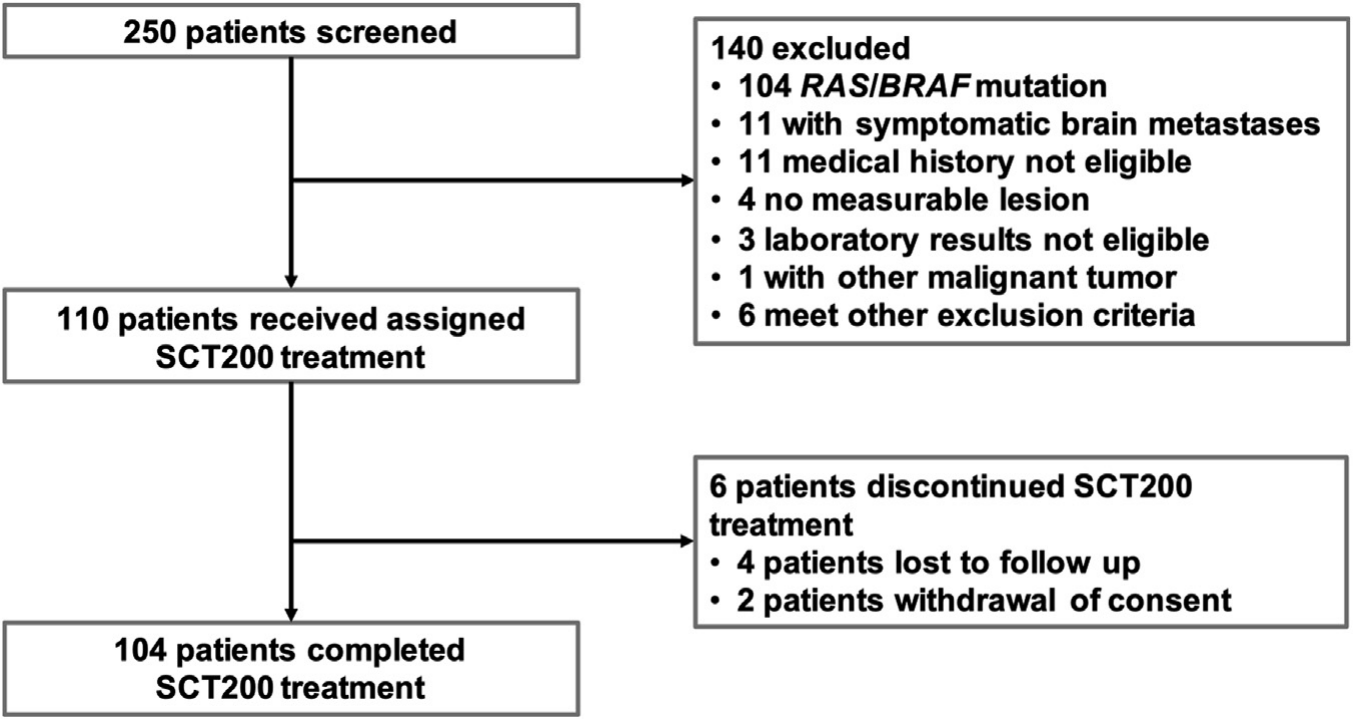
Workflow of this study.

From February 12, 2018 to December 1, 2019, a total of 250 patients with mCRC were screened from 22 hospitals in China, and 110 patients with fluorouracil, irinotecan and oxaliplatin refractory *RAS* and *BRAF* wild-type mCRC were ultimately enrolled in the FAS and SS (Fig. 1). In the FAS, the median follow-up was 14.1 months (IQR, 12.1–17.1; range, 0.49–20.73). The median age was 55 years (IQR, 47–63; range, 26–77), 62% (68/110) of whom were male, all baseline characteristics of the 110 eligible patients were summarized in Table 1. All patients had paired tumor tissue and blood samples at baseline.

**Table 1:** Baseline characteristics of SCT200 treated patients in the FAS (n = 110).

Baseline characteristics	Patients, n (%)
Age Median, years, (IQR)	55 (47–63)
Gender	
Male	68 (62%)
Female	42 (38%)
Ethnic group	
Han	100 (91%)
Non-Han	10 (9%)
ECOG PS score	
0	24 (22%)
1	86 (78%)
Location of primary site	
Left-sided	94 (85%)
Right-sided	14 (13%)
Others^a^	2 (2%)
Sites of involvement	
Liver	74 (67%)
Lung	71 (65%)
Lymph nodes	52 (47%)
Bone	17 (15%)
Peritoneum	12 (11%)
Paranephros	7 (6%)
Clinical stage	
ⅣA	34 (31%)
ⅣB	63 (57%)
ⅣC	13 (12%)
Previous lines to systemic therapy	
2	40 (36%)
≥3	70 (64%)
Previous therapy	
Surgery	100 (91%)
Radiotherapy	31 (28%)
Chemotherapy	110 (100%)
Targeted therapy^b^	67 (61%)

Note: Data are presented as the number of patients (%) or median (IQR).

Abbreviations: FAS, full analysis set; IQR, interquartile range; ECOG, Eastern Cooperative Oncology Group; PS, performance status.

a Others represents the patients with both left-sided and right-sided tumors.

b Sixty one percent of patients received prior targeted therapies including bevacizumab, apatinib, regorafenib, donafenib, and anlotinib.

### Efficacy

**Fig. 2:**
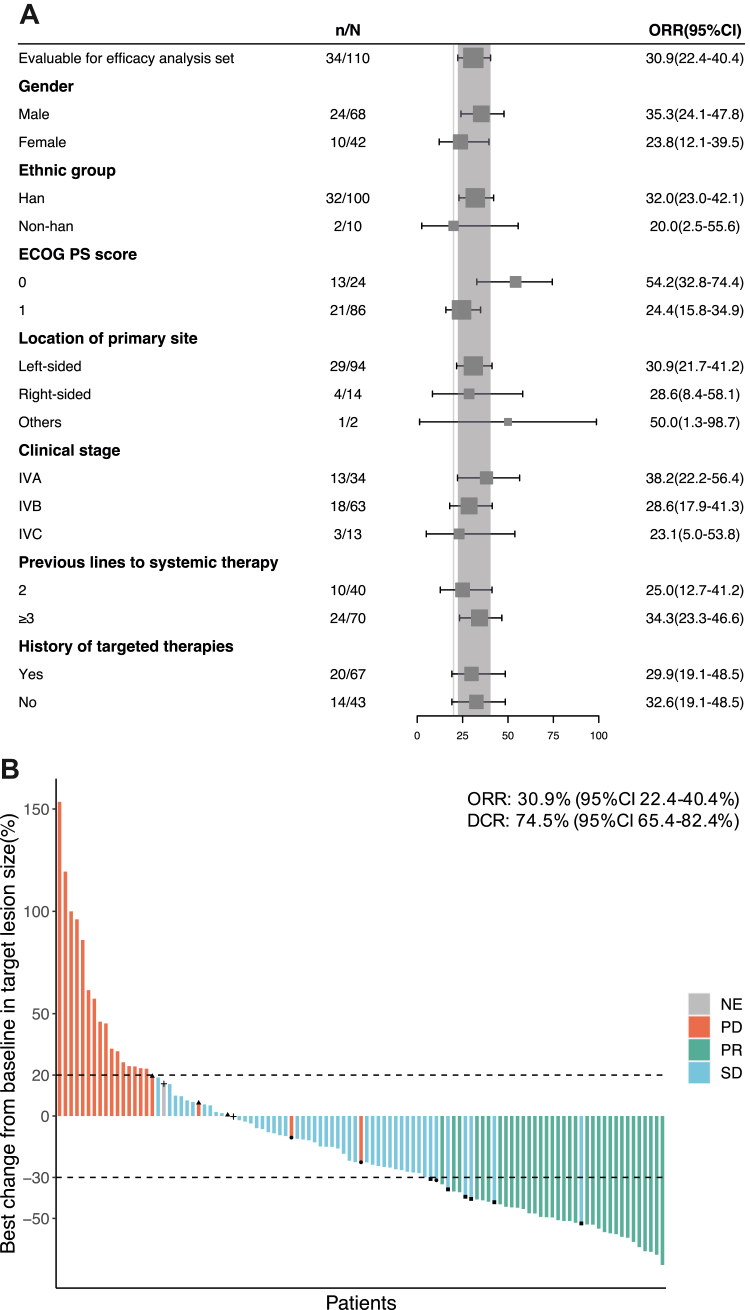
Efficacy of SCT200 in the FAS (n = 110). (A) Forest plot of ORR in different subgroups of patients. The grey vertical line at 20% represents the threshold of clinical efficacy in this study, the grey shadow represents the ORR (95%CI) of all patients in the FAS. (B) Waterfall plot of best percentage change from baseline as assessed by IRC (n = 105). Five patients were absent due to lack of post-treatment evaluation, including four NE and one PD due to non-target lesion progression. The dashed line at 20% and −30% indicate the thresholds for PD and PR, respectively. The dot represents new lesion. The triangle represents non-target lesion progression. The square represents PR not confirmed. The plus sign represents SD less than four weeks. FAS, full analysis set; ORR, objective response rate; CI, confidence interval; ECOG, Eastern Cooperative Oncology Group; PS, performance status; DCR, disease control rate; NE, not evaluable; PD, progressive disease; SD, stable disease; PR, partial response; IRC: independent review committee.

**Fig. 3:**
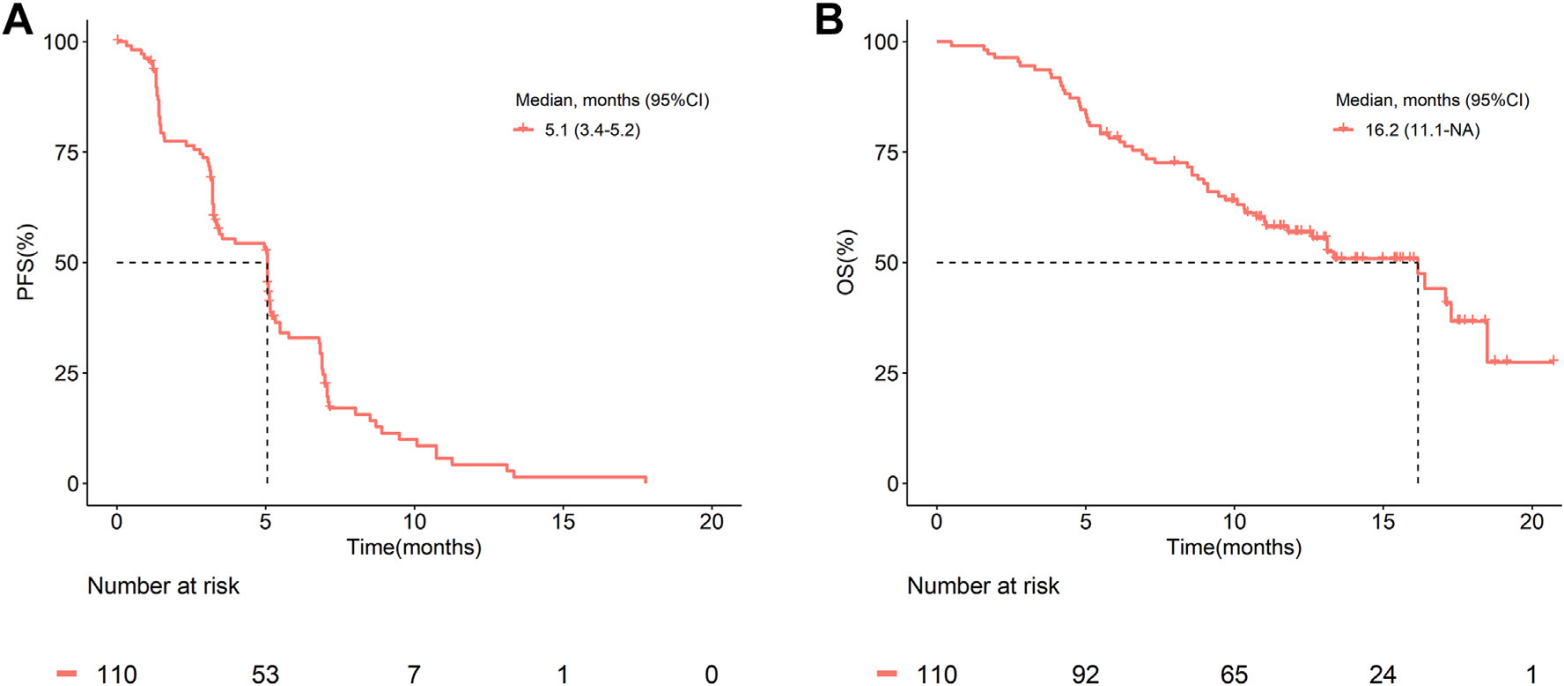
The Kaplan–Meier curves for survival in the FAS. Kaplan–Meier curve of (A) PFS and (B) OS. FAS, full analysis set; PFS, progression-free survival; CI, confidence interval; OS, overall survival; NA, not available.

The data cut-off date of this study was May 15, 2020. In the FAS, IRC-assessed ORR was 31% (34/110, 95% CI 22–40%). Objective responses were found across a variety of predefined subgroups, including gender, ethnic group, ECOG PS score, location of primary site, clinical stage, previous lines to systemic therapy, and history of targeted therapies (Fig. 2A). IRC-assessed DCR was 75% (82/110, 95% CI 65–82%), median DoR was 5.6 months (95% CI 4.0–7.4), and median TTR was 1.4 months (95% CI 1.3–1.4) (Table 2). The best response was PR in 34 (31%) patients. The tumor shrinkage assessed by IRC was seen in 75 of 105 (71%) patients (Fig. 2B). Five patients were absent due to lack of post-treatment evaluation (including four not-evaluable patients and one with PD due to non-target lesion progression). Median PFS and median OS was 5.1 months (95% CI 3.4–5.2) and 16.2 months (95% CI 11.1–not available [NA]), respectively (Fig. 3).

**Table 2:** Efficacy of SCT200 in the FAS (n = 110).

Efficacy	Patients, n = 110
BOR, n (%, 95% CI)	
PR	34 (31, 22–40)
SD	48 (44, 34–53)
PD	22 (20, 13–29)
NE	6 (5, 2–11)
ORR (CR/PR), % (n, 95% CI)	31 (34, 22–40)
DCR (CR/PR/SD), % (n, 95% CI)	75 (82, 65–82)
Median DoR (months, 95% CI)	5.6 (4.0–7.4)
Median TTR (months, 95% CI)	1.4 (1.3–1.4)
Median PFS (months, 95% CI)	5.1 (3.4–5.2)
Median OS (months, 95% CI)	16.2 (11.1-NA)

Abbreviations: FAS, full analysis set; BOR, best of response; CI, confidence interval; PR, partial response; PD, progressive disease; SD, stable disease; ORR, objective response rate; DCR, disease control rate; DoR, duration of response; TTR, time to response; PFS, progression-free survival; OS, overall survival; NE, not evaluable; NA, not available.

**Supplementary Figure S1:**
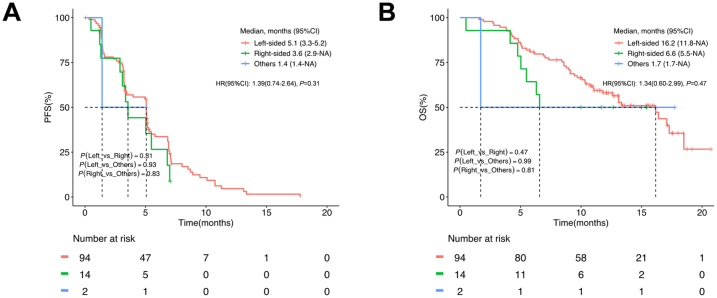


There are 94 left-sided mCRC patients, 14 right-sided mCRC patients and two others (patients with both left-sided and right-sided tumors). IRC-assessed ORR in patients with left-sided tumor was 31% (95% CI 22–41%). The median PFS of the patients with left-sided tumors was 5.1 months (95% CI 3.3–5.2), whereas that in patients with right-sided tumors was 3.6 months (95% CI 2.9-NA). The median OS of the patients with left-sided tumors was 16.2 months (95% CI 11.8-NA), whereas that in patients with right-sided tumors was 6.6 months (95% CI 5.5-NA). Although the patients with right-sided tumors showed worse survival benefit to the SCT200 treatment than the patients with left-sided tumors (hazard ratio [HR] 1.39 [95% CI 0.74–2.64] for PFS and HR 1.34 [95% CI 0.60–2.99] for OS), the difference is not significant (*P* = 0.31 for PFS, and *P* = 0.47 for OS) (Supplementary Figure S1).

### Safety

**Supplementary Figure S2:**
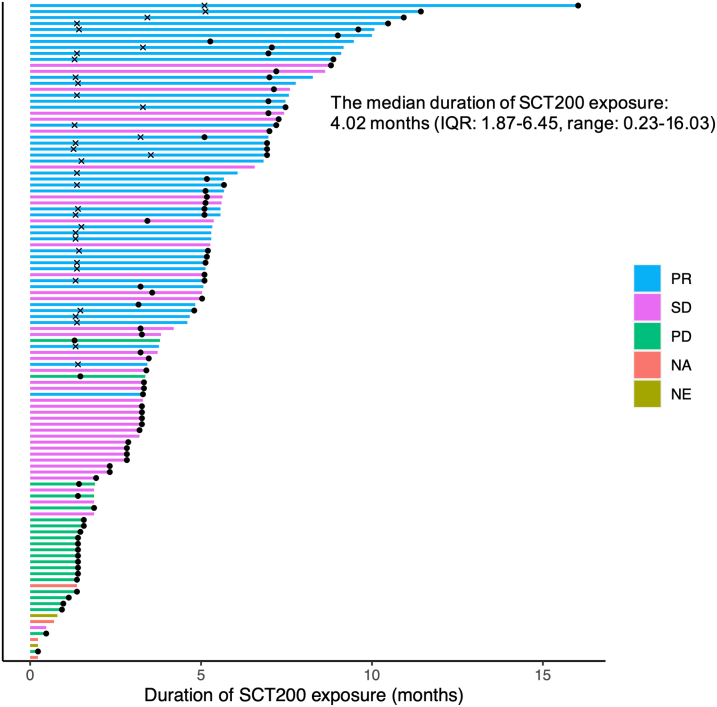


In the SS, the median duration of SCT200 exposure was 4.02 months (IQR, 1.87–6.45; range, 0.23–16.03) (Supplementary Figure S2). There were 11 (10%), 44 (40%), and 55 (50%) patients with exposure duration < six weeks, ≥six weeks to <16 weeks, and ≥16 weeks, respectively. As shown in Supplementary Table S1, all enrolled patients (110/110) experienced at least one TEAE and TRAE. Grade 3/4 TRAEs were reported in 45% (49/110) of patients. Twenty-six patients had TRAEs resulting in SCT200 interruption and eight patients had SCT200 dose reductions. Eight patients had treatment discontinuation due to TRAEs. SAEs were observed in 22% (24/110) patients, and 13 of them were treatment related. The most common ≥ grade 3 TRAEs were hypomagnesemia (17%, 19/110) and acneiform dermatitis (11%, 12/110). No deaths occurred (Table 3).

**Table 3:** Incidence ≥5% TRAE of SCT200 treated patients in the SS (n = 110).

	TRAE, n. (%)	
	Grade 1/2	Grade 3/4
Patients with at least one TRAE	61 (55)	49 (45)
Skin and subcutaneous tissue disorders		
Acneiform dermatitis	37 (34)	12 (11)
Rash	32 (27)	10 (9)
Metabolism and nutrition disorders		
Hypomagnesemia	49 (45)	19 (17)

Abbreviations: TRAE, treatment-related adverse event; SS, safety set.

### Baseline genomic modifiers of response

**Supplementary Figure S3:**
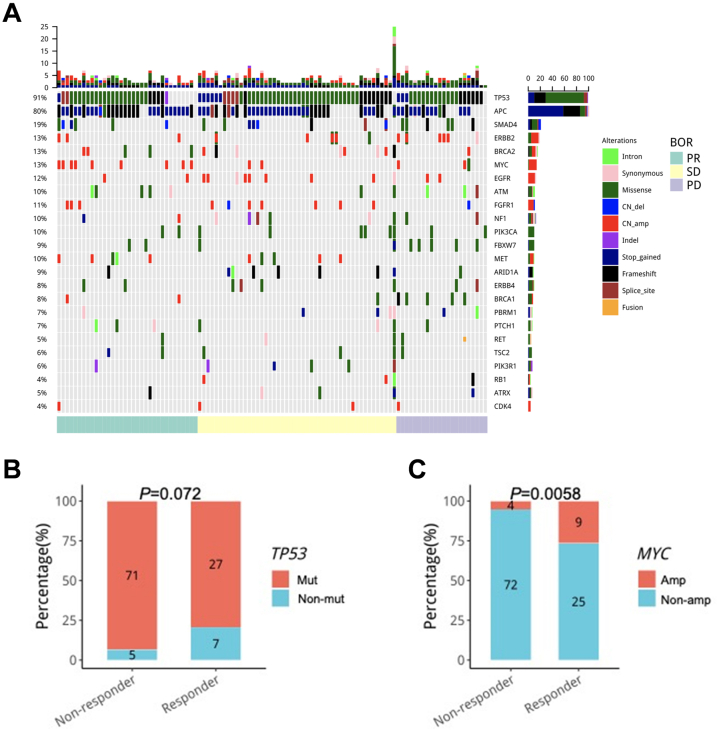


The alteration profile of the 110 patients who had baseline tumor FFPE samples for NGS analytical pipelines was summarized in Supplementary Figure S3A. Of them, the most commonly altered gene was *TP53* in 91% (101/110) of patients, followed by *APC* (80%, 89/110), *SMAD4* (19%, 20/110), *ERBB2* (13%, 14/110), *BRCA2* (13%, 14/110), *MYC* (13%, 14/110), *EGFR* (12%, 13/110), and *ATM* (10%, 10/110). Gene alteration profiles were classified according to patients’ clinical outcome in response to SCT200 treatment, to explore the distinct genomic profile between the responder group (patients with PR) and the non-responder group (patients with SD or PD). Notably, higher percentage of *TP53* mutation was seen in the non-responder group (93% [71/76] vs. 79% [27/34], *P* = 0.072) (Supplementary Figure S3B), while significantly higher percentage of *MYC* amplification was identified in the responder group (26% [9/34] vs. 5% [4/76], *P* = 0.0058) (Supplementary Figure S3C).

### Association between ctDNA abundance and clinical outcome

**Fig. 4:**
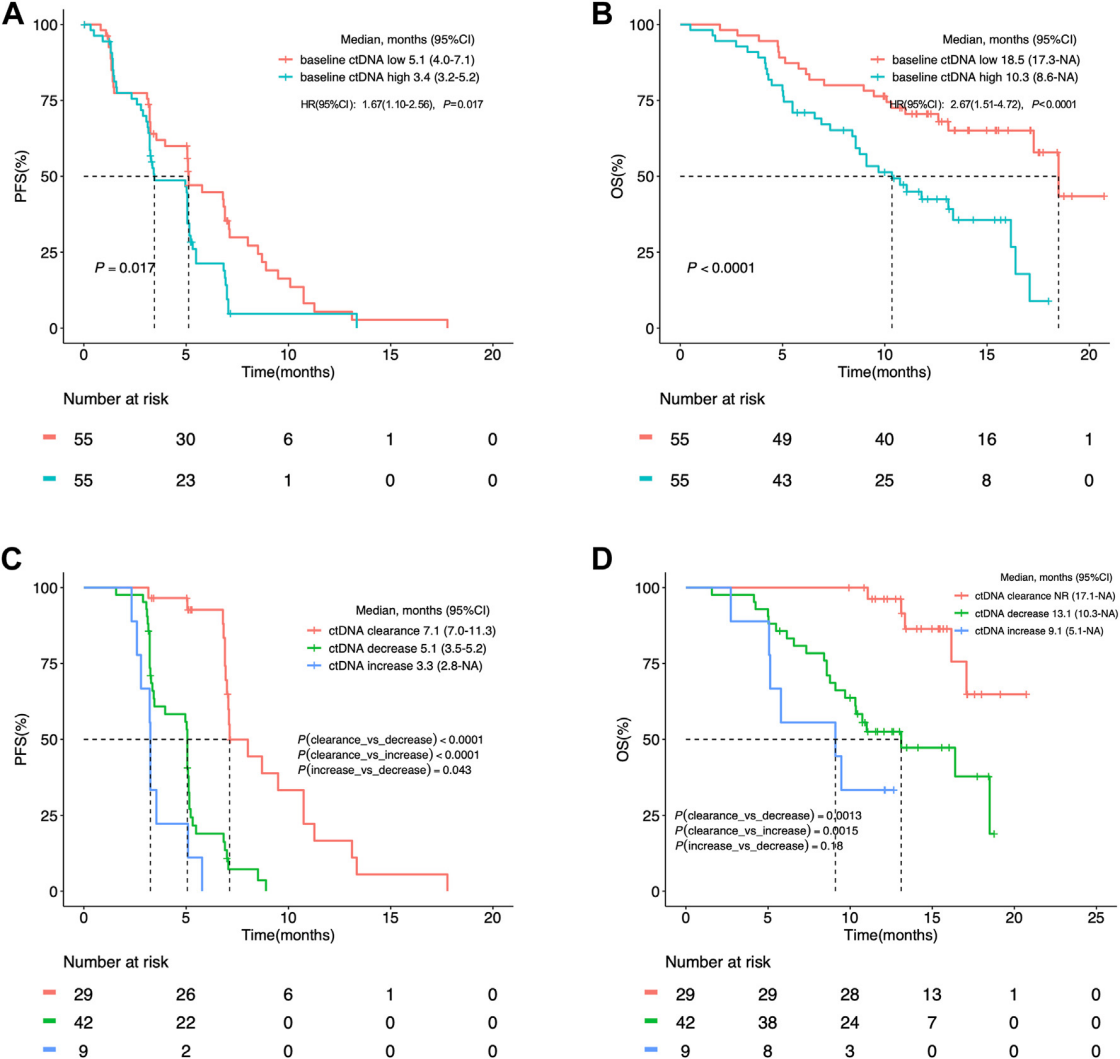
The Kaplan–Meier curves for survival stratified by baseline ctDNA abundance or ctDNA clearance status at the 7th week after the first dose of SCT200 administration before receiving SCT200. Kaplan–Meier curve of (A) PFS and (B) OS according to ctDNA level at baseline in all patients received SCT200 treatment (n = 110). Kaplan–Meier curve of (C) PFS and (D) OS according to ctDNA change between the 7th week after the first dose of SCT200 administration before receiving SCT200 and baseline and provided corresponding blood plasma samples (n = 80). CI, confidence interval; ctDNA, circulating tumor DNA; HR, hazard ratio; PFS, progression-free survival; OS, overall survival; NR, not reached.

Baseline ctDNA was analyzed and the cut-off value for ctDNA low and high was 0.0026 ng/μL. Of the 110 enrolled patients, all patients at baseline, 80 (73%) patients after seven weeks of SCT200 treatment, and 71 (65%) patients at disease progression provided blood plasma samples for ctDNA testing. At baseline, 55 (50%) patients were identified as ctDNA low and 55 (50%) patients were ctDNA high based on the cut-off value. Apparently, patients with low ctDNA abundance at baseline exhibited longer PFS and OS compared to patients with high ctDNA abundance (median PFS: 5.1 vs. 3.4 months, HR 1.67 [95% CI 1.10–2.56], *P* = 0.017; median OS: 18.5 vs. 10.3 months, HR 2.67 [95% CI 1.51–4.72], *P* < 0.0001) (Fig. 4A and B). After seven weeks of SCT200 treatment, ctDNA abundance was analyzed in 80 patients who provided blood plasma samples at the 7th week after the first dose of SCT200 administration before receiving SCT200 and compared with the abundance at baseline. The median PFS of patients with ctDNA clearance (n = 29, 36%), ctDNA decrease (n = 42, 53%) and ctDNA increase (n = 9, 11%) was 7.1 months (95% CI 7.0–11.3), 5.1 months (95% CI 3.5–5.2) and 3.3 months (95% CI 2.8–NA), respectively; whereas the median OS of them was not reached (95% CI 17.1 months–NA), 13.1 months (95% CI 10.3–NA) and 9.1 months (95% CI 5.1–NA), respectively. The median PFS of patients with ctDNA clearance was significantly longer than those with ctDNA decrease (*P* < 0.0001) or those with ctDNA increase (*P* < 0.0001). The median PFS of patients with ctDNA decrease was longer than those with ctDNA increase (*P* = 0.043) (Fig. 4C). The median OS of patients with ctDNA clearance was significantly longer than those with ctDNA decrease (*P* = 0.0013) or those with ctDNA increase (*P* = 0.0015). There was no significant difference of median OS between patients with ctDNA decrease and those with ctDNA increase (*P* = 0.18) (Fig. 4D).

**Supplementary Figure S4:**
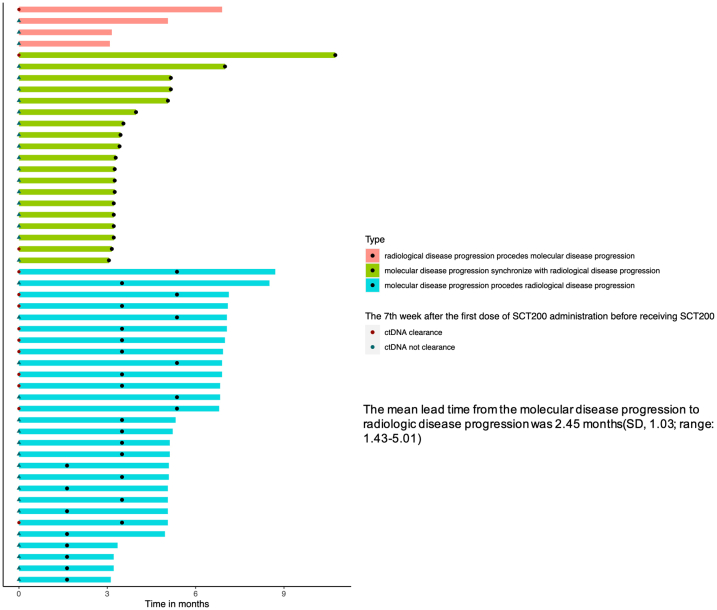


Among all patients who experienced radiological disease progression, 51 had a full ctDNA profile at no less than three time points: baseline, the 7th week after the first dose of SCT200 administration before receiving SCT200, and disease progression. Molecular disease progression in this study was defined as events which include emergence of novel alterations compared to baseline, baseline alterations with over 10% increase of allele frequency (AF), or detectable alterations following clearance. In total, 28 patients (28/51, 55%) experienced preceding molecular disease progression before radiological disease progression, and 19 patients (19/51, 37%) was found molecular disease progression at the same time with radiological disease progression. The mean lead time from the molecular disease progression to radiologic disease progression was 2.45 months (standard deviation 1.03; range, 1.43–5.01). Only four patients (4/51, 8%) had preceding radiological disease progression before molecular disease progression (Supplementary Figure S4).

### Prevalence of resistance mechanisms to SCT200 treatment

**Supplementary Figure S5:**
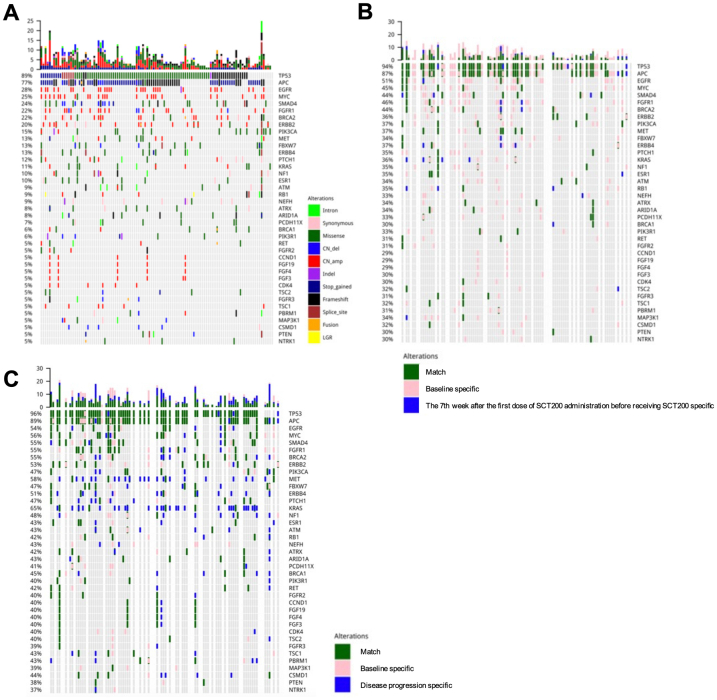


**Supplementary Figure S6:**
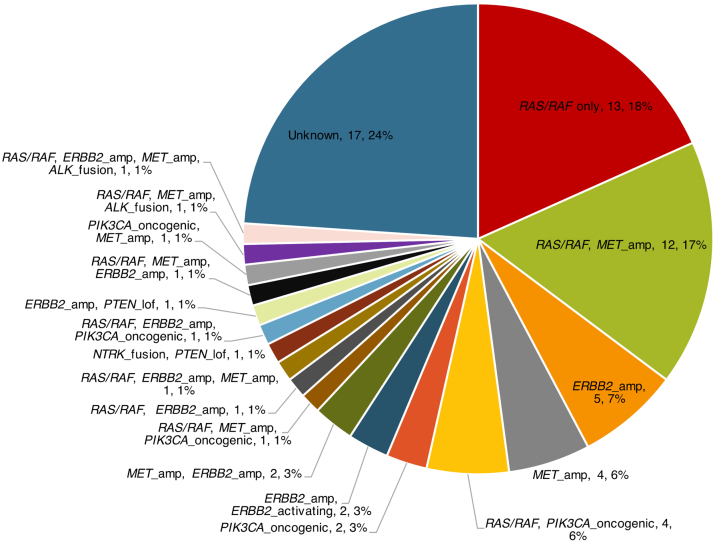


As Supplementary Figure S5 shown, the ctDNA alteration profiles exhibited marked discrepancies between time points (baseline vs. the 7th week after the first dose of SCT200 administration before receiving SCT200, and baseline vs. disease progression). Notably, much more somatic alterations were observed at disease progression than at the 7th week after the first dose of SCT200 administration before receiving SCT200. To figure out alterations associated with SCT200 treatment resistance, the differences of ctDNA alterations between baseline and disease progression were further compared and analyzed (Supplementary Figure S6). The *RAS/RAF* mutations (13/71, 18%), *RAS/RAF* mutations with *MET* amplification (12/71, 17%), *ERBB2* amplification (5/71, 7%), *MET* amplification (4/71, 6%) and *RAS/RAF* mutations with *PIK3CA* mutations (4/71, 6%) are the top five possible resistance mechanisms. To sum up, *RAS/RAF* mutations were identified in half of the patients at disease progression (36/71, 51%), and one third of patients harbored *MET* amplification (24/71, 34%). In up to 24% (17/110) of patients, the resistant alterations still remain unknown.

## Discussion

In this study, we evaluated the efficacy and safety of SCT200, a novel fully humanized IgG1 anti-EGFR mAb, and identified potential predictive biomarkers in patients with fluorouracil, irinotecan and oxaliplatin refractory *RAS* and *BRAF* wild-type mCRC. Median DoR was 5.6 months (95% CI 4.0–7.4), while the median DoR were 5.4 months (95% CI 3.8–5.5) for cetuximab and 3.8 months (95% CI 3.7–4.8) for panitumumab in ASPECCT study.^16^^,^^17^ The median PFS of SCT200 was 5.1 months in this study, while previous third-line regimen received a median PFS ranging from 4.2 to 4.7 months.^16^^,^^18^^,^^19^ The median OS of SCT200 was 16.2 months, the previous studies demonstrated a median OS ranging from 9.8 to 10.2 months.^16^^,^^18^^,^^20^ Several retrospective analysis has shown that primary tumor sidedness (PTS) might play a decisive role in the sensitivity to anti-EGFR mAbs therapy,^21^, ^22^, ^23^ and limited benefit was reported in patients with *RAS* wild-type right-sided tumor. However, few studies reported the association between the PTS of tumor and the clinical benefits of third-line anti-EGFR mAbs treatment in the Asian patient population.^24^ In this study, there was no statistically significant difference of survival benefits between *RAS/BRAF* wild-type patients with left-sided and right-sided tumor. However, as there are only 14 patients with right-sided tumor in this study, the power to detect statistical significance is low.

The SAEs was 34% of cetuximab and 30% of panitumumab as reported in ASPECCT study,^17^ while the SAE of SCT200 was 22% (24/110) in this study. Specifically, no treatment-related FAEs occurred in this study. Consistent with the previous reports, the most common ≥ grade 3 TRAEs were hypomagnesemia (17%, 19/110), acneiform dermatitis (11%, 12/110), and rash (9%, 10/110).^17^^,^^25^

It was reported that CRC was known to have 30–60% of *RAS* gene and 4–12% *RAF* gene mutated^26^, ^27^, ^28^ and the observed *KRAS* mutation (codons 12 and 13) rate was 32.3% (337/1042) in Chinese mCRC patients according to previous study.^29^ The landscape of *RAS*/*BRAF* mutation profiles during screening in this study (39%, 104/250) was comparable to the previous studies. Despite the clarified prognostic and predictive role of *RAS* gene mutations and *BRAF* V600E mutation in anti-EGFR mAbs treatment of mCRC patients, the prognostic and predictive value of alterations in other genes is still unclear.^30^^,^^31^ In this study, two possible predictive biomarkers, *TP53* mutation and *MYC* amplification, associated with SCT200 response in patients with fluorouracil, irinotecan and oxaliplatin refractory *RAS* and *BRAF* wild-type mCRC were identified. Emerging studies have shown the up-regulated levels of MYC protein as a downstream effector of frequently altered kinase MAPK and RAS pathways in CRC, contributing to primary and secondary resistance in targeted therapy.^32^^,^^33^ MYC expression has been found to distinguish patients with a shorter PFS and OS in anti-EGFR mAbs treated mCRC.^34^ However, in this study, *MYC* amplification was associated better anti-EGFR mAbs therapy response. As it might be the first study reporting the positive association of *MYC* amplification with anti-EGFR mAbs therapy response, the underlying molecular mechanism should be further investigated.

Numerous studies have revealed the advantage of ctDNA assessment in monitoring tumor response. Those results are mostly from retrospective analysis only based on blood samples. Our biomarker analysis in this study was based on paired tumor tissue and blood samples from all enrolled patients at baseline. Previously, only limited studies use targeted-sequencing panels with over 100 genes to explore the relationship between computed tomography assessed and molecular biomarkers in a prospective phase Ⅱ study. Compared with tracking of a single alteration like *KRAS* mutation in ctDNA, a panel is more informative due to the heterogeneous nature of CRC genomic alterations, which is supported by previous study.^35^ Thus, in this study, a panel with 108 genes was applied for better ctDNA analysis at both baseline and during follow-up. The association between baseline ctDNA level and survival has been clarified in several studies, especially about the prognostic role of *KRAS* or *BRAF* mutation status in ctDNA.^36^, ^37^, ^38^ Consistent with these results, result from this study also demonstrated better survival of patients with low baseline ctDNA. Although the association between absence of ctDNA after surgery and a better prognosis was reported, few studies illustrated such association in response to systemic therapy and most of the studies focused on alteration of *RAS/RAF* status during treatment. Of note, instead of focusing on alteration status of *RAS* or other EGFR pathway genes,^39^, ^40^, ^41^ here we defined ctDNA clearance as that none of the ctDNA alterations in the panel could be detected above the detection limit. The significantly longer PFS and OS were observed in patients with ctDNA clearance at the 7th week after the first dose of SCT200 administration before receiving SCT200. Since it is sometimes challenging to define the exact function of each alteration and their subsequent impact of prognosis, assessment of ctDNA clearance status at the 7th week after the first dose of SCT200 administration before receiving SCT200 would provide a timely and effective way to identify patients with worse clinical outcomes. Besides, a lack of correlation of ctDNA, *RAS* mutations and clinical outcomes has been reported in panitumumab-based therapies.^39^^,^^42^ To this end, the ctDNA clearance status at the 7th week after the first dose of SCT200 administration before receiving SCT200 might serve as a better generalized decision-making time point to guide the following treatment.

Traditional methods, like imaging based on RECIST version 1.1, would fail to recognize in real-time the actual onset of resistant clones due to the detection sensitivity, leading to a possible delay in the identification of drug resistance, whereas dynamic ctDNA analysis may overcome these limitations and forestall the radiological disease progression. For the surveillance of disease progression, *RAS* mutation emergence in ctDNA has been reported to precede radiological disease progression and the median lead time from the first detection of a *RAS* mutation to radiological disease progression was 3.6 months (range, −0.3 to 7.5).^42^ Similarly, in most of the patients (92%, 47/51), molecular disease progression as defined is no later than the radiological disease progression with a mean lead time of 2.45 months (standard deviation 1.03; range, 1.43–5.01) in this study.

This study was an open-label, single arm, multicenter, phase Ⅱ study lacking a comparison arm to characterize the effect of SCT200 vs. a third-line therapy like regorafenib, fruquintinib or other approved anti-EGFR mAbs. Also, this study was conducted in Chinese patients with mCRC only. Therefore, results of this study should be interpreted with caution in other ethnics.

This study revealed that SCT200 exhibited promising clinical efficacy and manageable safety profiles in *RAS* and *BRAF* wild-type mCRC patients progressed on fluorouracil, irinotecan and oxaliplatin treatment. The baseline ctDNA and ctDNA clearance status at the 7th week after the first dose of SCT200 administration before receiving SCT200 could be a potential prognostic biomarker for *RAS* and *BRAF* wild-type mCRC patients with SCT200 therapy.

## Contributors

YS and WG were responsible for the study conception and design; LY, WZ, NF, PC, YC, LZ, SL, HZ, YB, JZ, YD, YB, TL, MA, XY, KG, GD, JY, JS, YG, WL, GY, and YS contributed to patient enrollment, study care and data collection. YS, LY, WZ, LX, WG, and YW accessed and verified the data; YW and PM analyzed and interpreted the data; YS, WG, YW and PM wrote and revised the manuscript; YS supervised the study. All authors read and approved the final version of the manuscript.

## Data sharing statement

The authors declare that all the analysis data can be found in the main text and the supplementary files. The raw data generated in this study are not publicly available due to the privacy protection policy of personal medical data at our institution, but are available for non-commercial purposes from the corresponding author on reasonable request.

## Declaration of interests

Liangzhi Xie, Wenlin Gai, and Yan Wang are employees of Sinocelltech Ltd., Beijing, China. Peng Meng is employee of Burning Rock Biotech, Shanghai, China. The other authors declare that there is no conflict of interests regarding the publication of this article.

## Appendix A Supplementary data

Supplementary Table

renamed_c001c

## References

[1] Sung H., Ferlay J., Siegel R.L. (2021). Global cancer statistics 2020: GLOBOCAN estimates of incidence and mortality worldwide for 36 cancers in 185 countries. CA Cancer J Clin.

[2] Ogunwobi O.O., Mahmood F., Akingboye A. (2020). Biomarkers in colorectal cancer: current Research and future prospects. Int J Mol Sci.

[3] Saif M.W. (2010). Colorectal cancer in review: the role of the EGFR pathway. Expert Opin Investig Drugs.

[4] Sundar R., Tan I.B.H., Chee C.E. (2019). Negative predictive biomarkers in colorectal cancer: PRESSING ahead. J Clin Oncol.

[5] Food and Drug Administration (2004). https://wwwaccessdatafdagov/drugsatfda_docs/appletter/2004/125084ltrpdf.

[6] Giusti R.M., Shastri K.A., Cohen M.H., Keegan P., Pazdur R. (2007). FDA drug approval summary: panitumumab (Vectibix). Oncol.

[7] Xu S., Ramos-Suzarte M., Bai X., Xu B. (2016). Treatment outcome of nimotuzumab plus chemotherapy in advanced cancer patients: a single institute experience. Oncotarget.

[8] Yu J., Song Y., Tian W. (2020). How to select IgG subclasses in developing anti-tumor therapeutic antibodies. J Hematol Oncol.

[9] Zhang W., Han X., Yang L. (2022). Safety, pharmacokinetics and efficacy of SCT200, an anti-EGFR monoclonal antibody in patients with wild-type KRAS/NRAS/BRAF metastatic colorectal cancer: a phase I dose-escalation and dose-expansion study. BMC Cancer.

[10] Ciardiello D., Martini G., Famiglietti V. (2021). Biomarker-guided anti-Egfr rechallenge therapy in metastatic colorectal cancer. Cancers.

[11] Bekaii-Saab T., Kim R., Kim T.W. (2019). Third- or later-line therapy for metastatic colorectal cancer: reviewing best practice. Clin Colorectal Cancer.

[12] Zhang Y., Zou J.Y., Wang Z., Wang Y. (2019). Fruquintinib: a novel antivascular endothelial growth factor receptor tyrosine kinase inhibitor for the treatment of metastatic colorectal cancer. Cancer Manag Res.

[13] Sartore-Bianchi A., Pietrantonio F., Lonardi S. (2022). Circulating tumor DNA to guide rechallenge with panitumumab in metastatic colorectal cancer: the phase 2 CHRONOS trial. Nat Med.

[14] Mao X., Zhang Z., Zheng X. (2017). Capture-based targeted ultradeep sequencing in paired tissue and plasma samples demonstrates differential subclonal ctDNA-releasing capability in advanced lung cancer. J Thorac Oncol.

[15] Li Y.S., Jiang B.Y., Yang J.J. (2018). Unique genetic profiles from cerebrospinal fluid cell-free DNA in leptomeningeal metastases of EGFR-mutant non-small-cell lung cancer: a new medium of liquid biopsy. Ann Oncol.

[16] Price T., Kim T.W., Li J. (2016). Final results and outcomes by prior bevacizumab exposure, skin toxicity, and hypomagnesaemia from ASPECCT: randomized phase 3 non-inferiority study of panitumumab versus cetuximab in chemorefractory wild-type KRAS exon 2 metastatic colorectal cancer. Eur J Cancer.

[17] Price T.J., Peeters M., Kim T.W. (2014). Panitumumab versus cetuximab in patients with chemotherapy-refractory wild-type KRAS exon 2 metastatic colorectal cancer (ASPECCT): a randomised, multicentre, open-label, non-inferiority phase 3 study. Lancet Oncol.

[18] Vincenzi B., Santini D., Rabitti C. (2006). Cetuximab and irinotecan as third-line therapy in advanced colorectal cancer patients: a single centre phase II trial. Br J Cancer.

[19] Hsu H.C., Thiam T.K., Lu Y.J. (2016). Mutations of KRAS/NRAS/BRAF predict cetuximab resistance in metastatic colorectal cancer patients. Oncotarget.

[20] Kim T.W., Elme A., Kusic Z. (2016). A phase 3 trial evaluating panitumumab plus best supportive care vs best supportive care in chemorefractory wild-type KRAS or RAS metastatic colorectal cancer. Br J Cancer.

[21] Arnold D., Lueza B., Douillard J.Y. (2017). Prognostic and predictive value of primary tumour side in patients with RAS wild-type metastatic colorectal cancer treated with chemotherapy and EGFR directed antibodies in six randomized trials. Ann Oncol.

[22] Tejpar S., Stintzing S., Ciardiello F. (2017). Prognostic and predictive relevance of primary tumor location in patients with RAS wild-type metastatic colorectal cancer: retrospective analyses of the CRYSTAL and FIRE-3 trials. JAMA Oncol.

[23] Yin J., Cohen R., Jin Z. (2021). Prognostic and predictive impact of primary tumor sidedness for previously untreated advanced colorectal cancer. J Natl Cancer Inst.

[24] Chen K.H., Shao Y.Y., Chen H.M. (2016). Primary tumor site is a useful predictor of cetuximab efficacy in the third-line or salvage treatment of KRAS wild-type (exon 2 non-mutant) metastatic colorectal cancer: a nationwide cohort study. BMC Cancer.

[25] Petrelli F., Ardito R., Ghidini A. (2018). Different toxicity of cetuximab and panitumumab in metastatic colorectal cancer treatment: a systematic review and meta-analysis. Oncology.

[26] Siddiqui A.D., Piperdi B. (2010). KRAS mutation in colon cancer: a marker of resistance to EGFR-I therapy. Ann Surg Oncol.

[27] Cancer Genome Atlas N. (2012). Comprehensive molecular characterization of human colon and rectal cancer. Nature.

[28] Sanz-Garcia E., Argiles G., Elez E., Tabernero J. (2017). BRAF mutant colorectal cancer: prognosis, treatment, and new perspectives. Ann Oncol.

[29] Shi Y., Li J., Xu J. (2019). CMAB009 plus irinotecan versus irinotecan-only as second-line treatment after fluoropyrimidine and oxaliplatin failure in KRAS wild-type metastatic colorectal cancer patients: promising findings from a prospective, open-label, randomized, phase III trial. Cancer Commun.

[30] Capalbo C., Belardinilli F., Raimondo D. (2019). A simplified genomic profiling approach predicts outcome in metastatic colorectal cancer. Cancers.

[31] Lo Nigro C., Ricci V., Vivenza D. (2016). Prognostic and predictive biomarkers in metastatic colorectal cancer anti-EGFR therapy. World J Gastroenterol.

[32] Elbadawy M., Usui T., Yamawaki H., Sasaki K. (2019). Emerging roles of C-myc in cancer stem cell-related signaling and resistance to cancer chemotherapy: a potential therapeutic target against colorectal cancer. Int J Mol Sci.

[33] Xu A.M., Huang P.H. (2010). Receptor tyrosine kinase coactivation networks in cancer. Cancer Res.

[34] Strippoli A., Cocomazzi A., Basso M. (2020). c-MYC expression is a possible keystone in the colorectal cancer resistance to EGFR inhibitors. Cancers.

[35] Yao J., Zang W., Ge Y. (2018). RAS/BRAF circulating tumor DNA mutations as a predictor of response to first-line chemotherapy in metastatic colorectal cancer patients. Can J Gastroenterol Hepatol.

[36] Osumi H., Shinozaki E., Yamaguchi K., Zembutsu H. (2019). Clinical utility of circulating tumor DNA for colorectal cancer. Cancer Sci.

[37] Manca P., Corallo S., Busico A. (2021). The added value of baseline circulating tumor DNA profiling in patients with molecularly hyperselected, left-sided metastatic colorectal cancer. Clin Cancer Res.

[38] Moradi-Marjaneh R., Asgharzadeh F., Khordad E., Marjaneh M.M. (2021). The clinical impact of quantitative cell-free DNA, KRAS, and BRAF mutations on response to anti-EGFR treatment in patients with metastatic colorectal cancer. Curr Pharm Des.

[39] Kim T.W., Peeters M., Thomas A. (2018). Impact of emergent circulating tumor DNA RAS mutation in panitumumab-treated chemoresistant metastatic colorectal cancer. Clin Cancer Res.

[40] Yamada T., Matsuda A., Takahashi G. (2020). Emerging RAS, BRAF, and EGFR mutations in cell-free DNA of metastatic colorectal patients are associated with both primary and secondary resistance to first-line anti-EGFR therapy. Int J Clin Oncol.

[41] Peeters M., Price T., Boedigheimer M. (2019). Evaluation of emergent mutations in circulating cell-free DNA and clinical outcomes in patients with metastatic colorectal cancer treated with panitumumab in the ASPECCT study. Clin Cancer Res.

[42] Siena S., Sartore-Bianchi A., Garcia-Carbonero R. (2018). Dynamic molecular analysis and clinical correlates of tumor evolution within a phase II trial of panitumumab-based therapy in metastatic colorectal cancer. Ann Oncol.

