# Supplementary material for: Efficacy, safety and genomic analysis of SCT200, an anti-EGFR monoclonal antibody, in patients with fluorouracil, irinotecan and oxaliplatin refractory RAS and BRAF wild-type metastatic colorectal cancer: a phase Ⅱ study
Source: eBioMedicine. 2024 Jan 13;100:104966. doi: 10.1016/j.ebiom.2024.104966 (PMC10826138; doi:10.1016/j.ebiom.2024.104966)
Supplement: Supplementary Table [file mmc1.docx]

**Supplementary Online Content**

**Supplementary Table 1.** Overview of adverse events

**Supplementary Figure 1.** The Kaplan-Meier curves for survival stratified by primary sites

**Supplementary Figure 2.** Swimmer plot showing the exposure and response duration of SCT200

**Supplementary Figure 3.** Distribution of baseline mutations according to responses

**Supplementary Figure 4.** Swimmer plot of patients according to molecular PD versus radiological PD time

**Supplementary Figure 5.** The oncoprint diagrams of mutational landscape

**Supplementary Figure 6.** The pie chart of resistance mechanisms in patients with progressive disease

**Supplementary Table 1.** Overview of AEs in SCT200 treated patients in the SS (n=110)

|  | **AE, n (%)** |
| --- | --- |
| **TEAE** | 110 (100) |
| **TRAE** | 110 (100) |
| **Grade≥3 TEAEs** | 67 (61) |
| **Grade≥3 TRAEs** | 49 (45) |
| **SAE** | 24 (22) |
| **Treatment-related SAE** | 13 (12) |
| **FAE** | 4 (4) |
| **Treatment-related FAEs** | 0 (0) |
| **AESI** | 104 (95) |
| **Treatment-related AESI** | 104 (95) |
| **Infusion rate reduction due to TEAE** | 3 (3) |
| **Infusion rate reduction due to TRAE** | 3 (3) |
| **Dose reduction due to TEAE** | 8 (7) |
| **Dose reduction due to TRAE** | 8 (7) |
| **Dose interruption due to TEAE** | 33 (30) |
| **Dose interruption due to TRAE** | 26 (24) |
| **Discontinuation due to TEAE** | 12 (11) |
| **Discontinuation due to TRAE** | 8 (7) |

Abbreviations: TEAE, treatment-emergent adverse event; TRAE, treatment-related adverse event; SAE, serious adverse event; FAE, fatal adverse event; AESI, adverse event of special interest.

**Supplementary Figure 1**. The Kaplan-Meier curves for survival stratified by the location of primary site. Kaplan-Meier curve of (**A**) PFS and (**B**) OS in all patients according to the location of primary site (n=110). PFS, progression-free survival; CI, confidence interval; NA, not available; HR, hazard ratio; OS, overall survival

**Supplementary Figure 2.** Swimming plot for the exposure duration and response of SCT200. Each bar represents one subject and the patient’s response to the treatment of SCT200 is illustrated by different colors. The time when the objective response was first observed is indicated by an ×, and the time when the objective response was terminated is indicated by a circle. PR, partial response; SD, stable disease; PD, progressive disease; NA, not available, NE, not evaluated; IQR, interquartile range.

**Supplementary Figure 3**. Distribution of baseline alterations according to BOR. (**A**) The oncoprint diagram of genomic landscape at baseline, stratified by BOR. (**B**) *TP53* mutations associated with SCT200 responder or non-responder*. (**C**) *MYC* amplifications associated with SCT200 responder or non-responder*. *The responder group means patients with PR, and the non-responder group means patients with SD or PD. BOR, best overall response; PR, partial response; SD, stable disease; PD, progressive disease.

**Supplementary Figure 4**. Swimming plot of patients according to molecular disease progression versus radiological disease progression* time. Molecular disease progression: emergence of novel alterations compared to baseline, baseline alterations with over 10% increase of AF, or detectable alterations following clearance. *Radiological disease progression per RECIST version 1.1. ctDNA, circulating tumor DNA; SD, standard deviation; AF, allele frequency; RECIST, Response Evaluation Criteria in Solid Tumors.

**Supplementary Figure 5**. The oncoprint diagrams of genomic landscape at (**A**) baseline (n=110), (**B**) baseline versus the 7th week after the first dose of SCT200 administration before receiving SCT200 (n=80), and (**C**) baseline versus disease progression (n=71). The empty columns represent the missing sample collections at the 7th week after the first dose of SCT200 administration before receiving SCT200 or disease progression.

**Supplementary Figure 6.** The pie chart of resistance mechanisms in patients with disease progression. The number and percentage on the pie chart represent the number and percentage of patients harboring the corresponding gene alterations.
