# Supplementary material for: Efficacy, safety and genomic analysis of SCT200, an anti-EGFR monoclonal antibody, in patients with fluorouracil, irinotecan and oxaliplatin refractory RAS and BRAF wild-type metastatic colorectal cancer: a phase Ⅱ study
Source: eBioMedicine. 2024 Jan 13;100:104966. doi: 10.1016/j.ebiom.2024.104966 (PMC10826138; doi:10.1016/j.ebiom.2024.104966)
Supplement: renamed_c001c [file mmc2.pdf]

一项重组全人源抗人表皮生长因子受体(EGFR)单克隆抗体注射液（SCT200）治疗经氟尿嘧啶、奥沙利铂和伊立替康治疗失败的 RAS/BRAF 野生型转移性结直肠癌受试者的单臂、多中心的安全性和有效性研究

**方案编号**	SCT200mCRCII
**研究药物**	重组全人源抗人表皮生长因子<br>受体（EGFR）单克隆抗体注射液（SCT200）
**申办单位**	神州细胞工程有限公司
**牵头医院**	中国医学科学院肿瘤医院
**主要研究者**	石远凯教授
**版本号**	第 1.1 版
**版本日期**	2018-10-25

保密性声明

本方案中包含的所有信息的所有权归神州细胞工程有限公司，仅提供给研究者、合作研究者、伦理委员会和监督管理部门等相关的机构审阅。在未得到神州细胞工程有限公司书面的批准情况下，除在与可能参加本研究的受试者签署知情同意书时，向其做必要的解释外，严禁将任何信息告知与本研究无关的第三方

临床研究方案签字页

试验药物：重组全人源抗人表皮生长因子受体（EGFR）单克隆抗体注射液（SCT200）

试验名称：一项重组全人源抗人表皮生长因子受体(EGFR)单克隆抗体注射液(SCT200)治疗经氟尿嘧啶、奥沙利铂和伊立替康治疗失败的 RAS/BRAF 野生型转移性结直肠癌受试者的单臂、多中心的安全性和有效性研究

临床方案编号：SCT200mCRCII

研究者声明

我已收到研究者手册，并已知晓该试验药物的临床前研究的情况。我已被告知将及时收到更新的研究者手册。我已详细阅读并理解了本方案的内容，承诺将根据《药物临床试验质量管理规范》(GCP)的规定，认真履行研究者职责，按照本方案的设计及规定开展此项临床试验，并向本试验的所有工作人员详细说明有关试验的资料、规定和职责。

我将严格遵守《赫尔辛基宣言》，向受试者说明经伦理委员会批准同意的此项临床试验的详细情况，并取得知情同意书。我会负责作出与本临床试验相关的医疗决定，保证受试者在试验期间出现不良事件时得到适当的治疗。如在本试验过程中发生严重不良事件，我会立即采取适当的治疗措施以保障受试者的安全，同时报告 GCP 规定的相关单位。

我同意接受申办方派遣的监查员或稽查员及药品监督管理部门的视察，确保临床试验的质量。

我同意对所有收到的或试验中得到的与本方案有关的资料保密。

临床研究负责单位：中国医学科学院肿瘤医院

主要研究者签名 / 日期

________________________　　_____年_____月_____日

申办方声明

我公司将根据《药物临床试验质量管理规范》(GCP)规定，负责发起、申请、组织和稽查本项临床试验，并提供试验经费。特别对试验中发生的与试验相关的损害或死亡的受试者提供治疗的补偿，向研究者提供法律上与经济上的担保。

申办单位：神州细胞工程有限公司

项目负责人签名 / 日期

__________________________ _____年_____月_____日

目录

临床研究方案摘要 10
表 1 研究流程表 19
1. 研究背景 22
1.1 前言......22
1.1.1 结直肠癌流行病学及临床治疗现况......22
1.1.2 EGFR单抗药物简介......22
1.1.3 SCT200 介绍......24
1.2 SCT200 I期临床研究简介......24
1.2.1 研究设计......24
1.2.2 研究结果......25
2. 研究目的 28
2.1 主要目的......28
2.2 次要目的......28
3. 研究设计 28
3.1 研究设计原理......28
3.2 风险/获益评估......29
3.3 研究设计......30
3.3.1 研究总体设计......30
3.3.2 中心疗效评估委员会（IRC）......31
3.3.3 研究终点......31
4. 研究人群 32
4.1 受试者......32
4.1.1 入选标准......32
4.1.2 排除标准：......33
4.2 提前 35
4.2.1 研究者决定的提前退出......35
4.2.2 受试者自行退出研究......35
4.3 试验终止......35
5. 研究药物 36
5.1 研究药物基本信息......36
5.2 包装和标签......36
5.3 药物配制和给药......37
5.4 研究用药剂量调整原则......37
5.4.1 输液反应......37
5.4.2 皮肤毒性......37
5.5 暂停SCT200 治疗标准......39
5.6 重新开始SCT200 治疗的标准......39
5.7 给药超窗及其它停用SCT200 规定......39
5.8 研究用药注意事项......40
5.8.1 静脉输注期间的监护......40
5.8.2 输液反应的处理......40
5.8.3 皮肤毒性处理......40
5.8.4 腹泻、脱水和电解质紊乱处理......41
5.8.5 低镁血症处理......41
5.8.6 其他可能出现的不良事件......42
5.9 研究药物管理......42
5.9.1 运输/接收/储存/保管......43
5.9.2 发放/使用/回收/销毁......43

5.9.3 记录........43
5.10 受试者分配方法........43
5.11 设盲与揭盲规定........44
5.12 合并用药........44
5.12.1 合并用药/治疗........44
5.12.2 禁止用药/治疗........44
6. 研究流程及评估指标........45
6.1 研究流程........45
6.1.1 受试者入组原则........45
6.1.2 筛选期........45
6.1.3 研究治疗期........47
6.1.4 研究提前退出访视........48
6.1.5 随访期........48
6.2 有效性评估指标........50
6.2.1 主要疗效指标........50
6.2.2 次要疗效指标........50
6.3 安全性评估指标........50
6.3.1 安全性评估指标........50
6.3.2 免疫原性评估指标........51
6.4 探索性研究评估指标........51
7. 安全性评估........52
7.1 不良事件和严重不良事件处理........52
7.1.1 定义........52
7.1.2 不良事件严重程度........53
7.1.3 不良事件/严重不良事件与研究药物相关性判定........54
7.1.4 不良事件/严重不良事件的记录与报告........54
7.2 实验室检查异常值处理........55
7.3 妊娠事件处理........56
7.4 生命体征及体格检查........56
7.5 12-ECG不良事件........56
7.6 其他安全性考虑及风险管理........57
8. 统计分析........57
8.1 样本量........57
8.2 分析人群........58
8.3 统计分析方法........58
8.3.1 病例分布........59
8.3.2 基线及人口学特征........59
8.3.3 依从性及药物暴露分析........59
8.3.4 有效性分析........60
8.3.5 安全性分析........61
8.3.6 合并用药........62
8.3.7 亚组分析........62
9. 研究的开展和监督职责........62
9.1 申办方........62
9.2 伦理学和监管........63
9.2.1 伦理委员会........63
9.2.2 受试者知情同意........63
9.2.3 研究者........64
9.2.4 质量保证和检查要求........64
9.3 数据管理........65

9.3.1 数据录入......65
9.3.2 数据核查和质疑管理......65
9.3.3 数据库锁定......65
9.4 临床监查......66
9.5 医学监查......66
9.6 资料的保存......66
10. 研究结果注册及发表......66
11. 参考文献......68
12. 附件......69
附件一：ECOG评分......69
附件二：输液反应处理推荐......70
附件三：皮肤毒性反应处理推荐......72
附件四：实体肿瘤的疗效评价......73

缩略语表

ADCC	抗体依赖性的细胞介导的细胞毒作用
AE	不良事件
ALP	碱性磷酸酶
ALT	丙氨酸转氨酶(血清谷丙转氨酶)
APTT	部分凝血活酶时间
AST	天门冬氨酸转氨酶(血清谷草转氨酶)
ATC	解剖-治疗-化学分类系统
AUC	血药浓度-时间曲线下面积
BOR	最佳疗效
CDC	补体依赖的细胞毒性作用
CFDA	国家食品药品监督管理局
CHO	中国仓鼠卵巢细胞
Cmax	血药峰浓度
CR	完全缓解
CRF	病例报告表
CRO	合同研究组织
Cr	肌酐
CT	计算机体层摄影
CTCAE	常见不良事件术语标准
DCR	疾病控制率
DOR	缓解持续时间
ECOG	美国东部肿瘤协作组体力评分
EGFR	表皮生长因子受体
EMEA	欧洲药品管理局
FAS	全分析集
FDA	美国食品药物管理局
μg	微克
GCP	药物临床试验质量管理规范
GFR	肾小球滤过率

HBV	乙型肝炎病毒
HCV	丙型肝炎病毒
HIV	人类免疫缺陷病毒
β-hCG	人绒毛膜促性腺激素
ICH	人用药品注册技术要求国际协调会
IMP	研究用药物
ILD	间质性肺病
Kg	公斤
mAb	单克隆抗体
mCRC	转移性结直肠癌
MedDRA	国际医学用语词典
mg	毫克
mL	毫升
mmHg	毫米汞柱
MMP	医学监查计划
MRI	磁共振成像
nM	纳摩尔
NE	无法评价
NOAEL	未见明显毒性反应剂量
NYHA	纽约心脏协会
ORR	客观缓解率
PD	疾病进展
PFS	无进展生存期
PK	药代动力学
PPS	符合方案集
PR	部分缓解
PT	凝血酶原时间
QA	质量保证
QW	每周一次
Q2W	每两周一次

RECIST	实体肿瘤疗效评价标准
SAE	严重不良事件
SAP	统计分析计划
SD	疾病稳定
SOC	系统器官分类
SPF	防晒系数
SS	安全性集
TBIL	总胆红素
TEAE	治疗期间不良事件
TKI	酪氨酸激酶抑制剂
TTR	至缓解时间
VEGF	血管内皮生长因子
WBC	白细胞

临床研究方案摘要

**申办方名称**	神州细胞工程有限公司
**药物名称**	SCT200
**活性成分**	重组全人源抗人表皮生长因子受体（EGFR）单克隆抗体
**样本量**	110 例
**方案编号**	SCT200mCRCII
**研究简短标题**	SCT200 治疗经氟尿嘧啶、奥沙利铂和伊立替康治疗失败的RAS/BRAF野生型转移性结直肠癌的有效性和安全性
**研究标题**	一项重组全人源抗人表皮生长因子受体(EGFR)单克隆抗体注射液(SCT200)治疗经氟尿嘧啶、奥沙利铂和伊立替康治疗失败的RAS/BRAF 野生型转移性结直肠癌受试者单臂、多中心的安全性和有效性研究
**研究中心**	约 15 家研究中心
**适应症**	转移性结直肠癌
**研究启动（计划）**	2017 年 12 月
**研发分期**	II 期
**主要目的**	评估SCT200静脉给药在经氟尿嘧啶、奥沙利铂和伊立替康治疗失败的RAS/BRAF野生型转移性结直肠癌受试者中的有效性。
**次要目的**	• 评估SCT200静脉给药在经氟尿嘧啶、奥沙利铂和伊立替康治疗失败的RAS/BRAF野生型转移性结直肠癌受试者中的安全性<br>• 评估SCT200静脉给药在经氟尿嘧啶、奥沙利铂和伊立替康治疗失败的RAS/BRAF野生型转移性结直肠癌受试者中的免疫原性。
**研究设计**	本研究为一项单臂、多中心的II期临床研究，以评估SCT200在经氟尿嘧啶、奥沙利铂和伊立替康治疗失败的RAS/BRAF野生型转移性结直肠癌受试者中的有效性和安全性。本研究包括3个阶段：筛选期、治疗期和随访期。

**筛选期**	受试者签署知情同意书后，进入研究筛选期。根据获得实验室检查结果的时限，筛选期最长28天，期间受试者将按照研究流程表（**表1**）中的访视计划，完成筛选期评估。符合研究所有入选标准且不符合排除标准的受试者将进入研究治疗期。
**治疗期**	进入治疗期的受试者将接受SCT200 6.0mg/kg，连续 6周每周给药1次，之后接受8.0mg/kg，每2周给药1次，持续给药治疗。SCT200静脉输注给药，每位受试者用药量将根据其体重计算得出。输注过程中需密切观察受试者面色、是否有出汗或头痛等，以便及时发现输注反应相关临床表现。受试者接受研究用药治疗后，按访视计划行疗效评估和安全性检查。本研究筛选期及整个研究期间的肿瘤评估，将依据实体瘤疗效评价标准（RECIST版本1.1）。应尽可能采用增强的计算机断层扫描（CT），如受试者对造影剂过敏，可替换为磁共振成像（MRI））检查进行疗效评估，每位受试者的检查方法在研究期间应保持一致。<br><br>如疗效评估为疾病稳定、部分缓解或完全缓解，可允许继续治疗直至出现疾病进展（PD）或不可耐受的毒性、撤销知情同意或死亡或开始后续抗肿瘤治疗。在治疗期间，如出现研究药物相关不良事件，研究者应对受试者风险/利益进行临床综合评估后，根据方案第5.4章节规定的研究药物剂量调整说明对受试者在后续治疗期间的研究用药剂量调整/停用药物作出决定。在治疗期间，受试者应根据方案访视操作流程，接受安全性评估（每4周1次）及疗效评估（在结束每周给药方案即研究第7周时进行首次疗效评估，后续每8周进行1次疗效评估）直至发生PD、出现不可耐受的研究用药相关不良事件、开始后续抗肿瘤治疗、死亡或失访。如果发生了提示PD的临床症状，需安排计划外访视以完成影像学评估确认。
**随访期**	受试者在结束研究治疗后进入随访期，随访期包括安全性随访、疾病进展随访和生存情况随访。所有至少接受过一剂研究治疗的受试者均需进行安全性随访访视，访视时间为最后一剂研究用药后 28

<table>
<tr><td></td><td>（+7）天，期间需按访视流程进行评估。
在安全性随访后，对于尚未发生 PD 且未开始后续抗肿瘤治疗的受试者，将继续每 8 周进行 1 次疗效评估，其操作流程与研究治疗期疗效评估相同，直至发生 PD、开始后续抗肿瘤治疗、死亡或失访。
在安全性随访访视和/或疾病进展随访访视后，将根据标准的临床治疗进行生存情况随访。所有至少接受过一次 SCT200 治疗的受试者均需进行生存情况随访，这些访视计划在安全性随访/疾病进展随访访视后每 3 个月（±14 天）1 次，可通过电话问询方式进行，以收集安全性访视后开始的后续抗肿瘤治疗以及生存状况信息。</td></tr>
<tr><td>研究结束</td><td>本研究结束时间点定义为直至至少 85%受试者出现疾病进展、开始后续抗肿瘤治疗、死亡、提前退出研究或失访。此时仍在接受研究用药的受试者将由研究者基于临床综合判断决定其后续适当的临床治疗/处理。如研究者认为受试者继续用药仍将有临床获益，可继续给予受试者临床研究用药。</td></tr>
<tr><td>研究
流程图</td><td>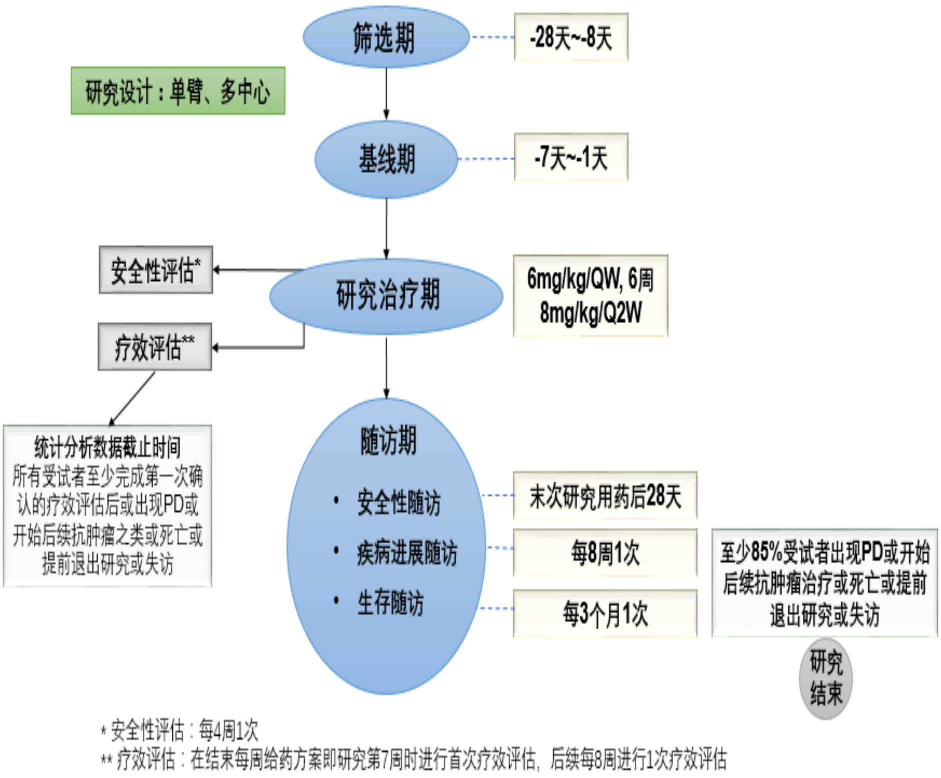
</td></tr>
</table>

**入选/排除标准**	**入选标准：**<br>*符合下列所有标准的受试者可入选本研究：*<br>1) 自愿参加本项临床试验并签署知情同意书；<br>2) 年满 18 周岁，性别不限；<br>3) 预计生存期≥3 个月；<br>4) ECOG 体力状况评分 0～1 级；<br>5) 经病理学检查确诊为转移性结直肠腺癌；<br>6) 受试者既往接受过标准化疗方案治疗并失败。这些标准治疗方案中必须包含氟尿嘧啶类、奥沙利铂、伊立替康。治疗失败的定义为：治疗过程中或末次治疗后的 6 个月内出现疾病进展或毒副作用不可耐受。注：<br>a) 标准治疗方案治疗以至疾病进展为止，用药时间≥1 个周期的一种或多种化疗药物；<br>b) 允许前期进行辅助/新辅助治疗。如果辅助/新辅助治疗期间或者完成后 6 个月内出现复发或转移，则认为辅助/新辅助治疗是一个针对进展期疾病的一线前期全身化疗的失败 ；<br>7) 肿瘤组织状态为 RAS 和 BRAF 基因野生型；<br>8) 实验室检查：<br>i. 血常规：中性粒细胞≥$1.5\times l0^{9}$/L，血小板≥75×10^{9}/L，血红蛋白≥80g/L；<br>ii. 肝功能：谷丙转氨酶（ALT）和谷草转氨酶（AST），无肝转移者 ALT 和 AST≤正常值上限×3，有肝转移者 ALT 和 AST≤正常值上限×5；总胆红素(TBIL)≤正常值上限×1.5；<br>iii. 肾功能：肌酐(Cr)≤正常值上限×1.5；<br>iv. 电解质：镁≥正常下限；<br>9) 根据 RECIST 标准 1.1 版，至少有一个经 CT 或 MRI 检查显示最长径≥10mm（扫描厚度不超过 5.0mm），淋巴结：短径≥15mm，可测量的肿瘤病灶（非放射治疗野）；

	**排除标准：**<br>*符合以下任一标准的受试者应从本研究中排除：*<br>1) 筛选前已知有中枢神经系统转移或有中枢神经系统转移病史的患者。对于临床疑似中枢神经系统转移的患者，随机化前28天内必须进行影像学确认，排除中枢神经系统转移；<br>2) 有其他恶性肿瘤病史，除外：在入组前5年或5年以上恶性病灶已经过治疗性措施的处理且不存在已知的活跃病灶，研究者判断复发风险较低；接受充分治疗的非黑色素瘤皮肤癌，且无病情恶化证据；接受充分治疗的原位宫颈癌，且无病情恶化证据；前列腺上皮内瘤，无前列腺癌复发证据；<br>3) 已知对试验药物中抗体或所含其它成分过敏者；<br>4) 曾接受EGFR抗体（如，帕尼单抗、西妥昔单抗或其类似物），或小分子EGFR抑制剂（如，吉非替尼、埃罗替尼、拉帕替尼等）；<br>5) 在入组前4周或4周内，接受过抗肿瘤药物治疗（如，化疗、激素治疗、免疫治疗、抗体治疗、放疗）或接受研究药物治疗，或入选时受试者仍存在之前抗肿瘤治疗导致的≥2级毒副反应（除外脱发和奥沙利铂引起的≤2级神经毒性）；<br>6) 受试者当前已入组至其他研究器械或研究药物研究中，或距离其他研究药物或研究器械停用时间少于或等于4周；<br>7) 在入组前4周或4周内接受过大手术（如需要全身麻醉），受试者须从与手术相关的损伤中恢复；<br>8) 入组前2周内接受过输血、促红细胞生成素（EPO）、粒细胞集落刺激因子（G-CSF）或粒细胞-巨噬细胞集落刺激因子（GM-CSF）的治疗；<br>9) 有临床意义的心血管疾病（即定义为：不稳定型心绞痛、有症状的充血性心力衰竭（纽约心脏病协会[NYHA]≥II 级）、不可控的严重心律失常）；<br>10) 在入组前6个月内发生过心肌梗死；

	11) 有间质性肺病（ILD）病史，如间质性肺炎、肺纤维化，或在基线胸部 CT 或 MRI 上显示有 ILD 证据；<br>12) 有临床症状，需要临床干预或稳定时间小于 4 周的浆膜腔积液（如胸腔积液和腹水）；<br>13) 可能干扰结果解释的医疗或精神病史或实验室异常病史；<br>14) 妊娠期或哺乳期的受试者，或计划在治疗期间及治疗结束后 6 个月内妊娠的受试者；<br>15) 在治疗期间与治疗结束后 6 个月内，不愿意接受有效避孕措施的受试者（包括男性或女性受试者）；<br>16) 患者处于乙肝或丙肝活动期（既往有乙肝感染病史，无论是否服用药物控制，HBV DNA≥10^4拷贝数或者≥2000 IU/ml；丙肝感染，HCV RNA≥15 IU/ml）；或人免疫缺陷病毒（HIV）抗体阳性（如无临床证据提示可能存在HIV感染时，无需检测）；<br>17) 在入组前 2 周或 2 周内，受试者存在需要系统治疗的活动性感染或不可控感染（简单泌尿道感染或上呼吸道感染除外）；<br>18) 已知受试者存在酒精或药物成瘾；<br>19) 研究者认为受试者存在可能影响其对方案依从性和研究指标评估的其他状况，不适宜参加研究的受试者。
**研究治疗给药方法**	给药方案：SCT200 6.0mg/kg，连续 6 周每周给药 1 次，之后接受 8.0mg/kg，每 2 周给药 1 次，持续给药治疗。<br>每周给药阶段，建议每周第 1 天给药，研究用药时间窗为±2 天。每 2 周给药阶段，建议与每周给药时间点保持平行（如，每周给药周三进行，每 2 周给药在第一周的周三进行），研究用药时间窗为±3 天。原则上应保证相邻两次研究用药间隔为 7/14 天，如因 AE 或其他原因导致研究用药延后（如从周一延后至周三用药），后续研究用药以调整后的每周固定天给药（如周三用药）。如果受试者因 AE 未恢复至可用药水平延迟给药超窗，受试者将在研究者判断其可再次用药时接受研究给药，在后续给药周应按调整后的用药日接受给药（如

	从周一调整至周四，后续在周四接受研究用药）。如因其他原因延迟给药超窗，研究用药日调整原则同上。患者接受研究治疗直至出现疾病进展或不可耐受的毒性、撤销知情同意或死亡或开始后续抗肿瘤治疗。SCT200 静脉输注给药，每位受试者用药量将根据其体重计算得出。
合并用药/治疗	所有从签署知情同意书到安全性随访访视期间给予的药物（非治疗目的用药除外，如配液用生理盐水等）均将记录在 CRF 中。也将记录药物增减或剂量变更情况。<br>入组后研究者可根据本研究人群的临床用药常规进行决定，给予受试者镇静剂、止吐剂、抗生素、镇痛剂、抗组胺药物、类固醇、粒细胞集落刺激因子以及红细胞、红细胞生成素、血小板或新鲜冷冻的血浆输液制品，以帮助治疗疼痛、感染和其他恶性肿瘤并发症。对研究过程中发生低镁血症，可予临床常规治疗，具体内容见 5.8.5 章节。如发生发热性中性粒细胞减少或有证据提示感染，可给予临床常规抗感染治疗。<br>对于出现的皮肤毒性相关 AE，应积极治疗，必要时请皮肤科医生会诊。允许给予局部和/或口服抗生素治疗（参见第 5.8.3 节，皮肤毒性处理）。<br>如果在试验治疗期间考虑进行任何局部放疗，例如因骨痛加重，应首先根据 RECIST 版本 1.1 确认该受试者是否发生 PD。根据 RECIST 版本 1.1 评估的靶和非靶病灶，如果在试验期间接受过放射治疗，则不得进一步用于缓解评估。发生骨转移时可给予双磷酸盐。如果因骨痛加重考虑增加正在进行的双磷酸盐治疗剂量或开始双磷酸盐治疗，首先根据 RECIST 1.1 版本确认该受试者是否发生 PD。
禁用药物/治疗	入组后不得给予受试者额外的系统性免疫治疗、化疗、放疗、用于治疗癌症的激素治疗或任何其他研究药物。<br>入组后不允许给予任何被批准用于抗癌治疗的中药（中药说明书中有抗癌或抗肿瘤字样的不允许使用）。如需要，研究者可决定给予非抗癌治疗适应症的中药，例如支持性治疗的中药。

<table>
<tr><td rowspan="2">研究终点</td><td>主要终点
结直肠癌患者总体人群中的客观缓解率（ORR）</td></tr>
<tr><td>次要终点
次要疗效终点：左半结直肠癌患者人群中的ORR、最佳疗效（BOR），疾病控制率（DCR），缓解持续时间（DOR），至缓解时间（TTR），无进展生存期（PFS），PFS率（6 个月、9 个月和 12 个月），总生存期（OS）。
上述疗效终点中的肿瘤治疗反应均依据实体瘤疗效评估标准（RECIST）版本 1.1 由中心审阅进行评估。首次出现疗效缓解需在 4 周后进行疗效确认影像学检查。
左/右半结肠定义[1]：右半结肠包括盲肠、升结肠和近端 2/3 的横结肠，左半结肠包括远端 1/3 的横结肠、降结肠、乙状结肠和直肠。
安全性评估终点：TEAE、临床实验室检查值、生命体征、12-ECG、免疫原性，体格检查（包括皮肤毒性反应）。</td></tr>
<tr><td>统计学方法</td><td>分析集：
全分析集（full analysis set, FAS）：包括所有接受至少一次研究用药的受试者。
安全性集（safety set，SS）：包括所有接受至少一次研究用药，且具有至少一次用药后安全性评估的受试者。
符合方案集（per-protocol set, PPS）:包括 FAS 中所有研究期间未使用影响有效性评价的合并用药、主要评价指标数据完整且无重大试验方案违背的受试者。导致患者被排除在符合方案集之外的所有试验方案偏离将在统计分析计划（SAP）中详细描述，并在数据锁定前完成。
有效性分析：
在全分析集上对疗效指标进行分析。帕尼单抗与西妥昔单抗在相近研究人群中ORR分别为22.0%和19.8%（参考ASPECCT研究），在单侧$\alpha = 0.025$的水平上，对主要疗效指标ORR进行如下优效性假设检验：</td></tr>
</table>

	H_0: ORR ≤ 20% vs. H_1: ORR > 20%。<br>给出ORR观察值并提供95%确切（exact）置信区间。如果ORR观察值大于35%，且95%置信区间下限大于20%，则认为试验药在治疗结直肠癌患者总体人群中具有显著的临床意义。<br>**数据分析和截止时间：**<br>计划统计分析的数据截止时间为所有受试者至少完成第一次确认的疗效评估后，或至出现以下情况：疾病进展、开始后续抗肿瘤治疗、死亡、提前退出研究或失访（即统计分析数据截止时间点不早于上述时间点）。
**样本量依据**	试验计划入组并治疗 110 例结直肠癌患者，考虑 10%的脱落率，可提供 99 例的有效性分析样本量。该样本量可在单侧 α=0.025 的水平上提供>93%的把握度检测到 15%的优效差异（相对于 20%的历史对照），并为 ORR 的估计提供的精度为 20.4%以上（即 95%置信区间宽度≤ 20.4%）。

表 1 研究流程表

研究项目	筛选期		研究治疗期					研究提前退出访视[1]	随访期		
**访视**	**1**	**2（基线[2-3]）**	首次用药	研究用药	研究用药	安全性评估[4]	疗效评估[5]	-	安全性随访	疾病进展随访	生存情况随访
**研究天数/事件频率**	**-28**	**-7~-1**	**0**	每周 **1** 次	每 **2** 周 **1** 次	每 **4** 周 **1** 次		-	末次研究用药后 **28** 天	每 **8** 周 **1** 次	每 **3** 个月 **1** 次[6]
**时间窗（天）**	**-**	**-**	**-**	**±2**	**±3**	**±7**			**+7**	**±7**	**±14**
知情同意书	X										
肿瘤诊断	X										
RAS、BRAF评估[7]	X										
人口统计学	X										
既往抗肿瘤相关治疗	X										
病史、手术史、治疗史	X										
体格检查	X	X		X	X			X	X	X	
体重[8]		X							X	X	
生命体征	X	X	X	X	X			X	X	X	
12 导联心电图	X	X				X		X	X	X	
ECOG 体能状态	X	X				X		X	X	X	
血常规、尿常规、血生化、凝血功能[9]	X	X				X		X	X	X	
免疫原性[10]		X		X							
探索性研究标本采集[11]	X										
妊娠试验[12]	X							X	X		

研究项目	筛选期		研究治疗期					研究提前退出访视[1]	随访期		
**访视**	**1**	**2（基线[2-3]）**	首次用药	研究用药	研究用药	安全性评估[4]	疗效评估[5]	-	安全性随访	疾病进展随访	生存情况随访
**研究天数/事件频率**	**-28**	**-7~-1**	**0**	每周 **1** 次	每 **2** 周 **1** 次	每 **4** 周 **1** 次		-	末次研究用药后 **28** 天	每 **8** 周 **1** 次	每 **3** 个月 **1** 次[6]
**时间窗（天）**	-	-	-	**±2**	**±3**	±7			**+7**	**±7**	**±14**
HBV、HCV、HIV 检查	X										
肿瘤评估[13]	X						X	X	X	X	
入选/排除标准确认	X	X									
SCT200 静脉给药[14]			X	X	X						
合并用药记录	X	X	X	X	X	X	X	X	X		
不良事件收集	X	X	X	X	X	X	X	X	X	X[15]	X[15]
后续抗肿瘤治疗									X	X	X
死亡情况									X	X	X

备注：

1. 受试者撤回知情同意（研究期间拒绝继续接受治疗、出现 PD 或不可耐受毒性）并拒绝进行安全性随访和生存情况随访，需尽可能在离开本研究前完成研究提前退出访视相关检查项；
2. 基线期所有实验室检查应该在首次研究用药前 3 天内完成。如访视 1 某些实验室检查日期满足此要求，相应检测在基线期可不复测；
3. 基线期体格检查、生命体征、12 导联心电图、ECOG 体能状态评分可接受 7 天（1 周内）结果。如访视 1 相关检查满足此要求，基线期可不复测；
4. 安全性评估：研究治疗期间，每 4 周接受一次完整的安全性评估。此外，每次接受研究用药前 3 天内，均应完成基本的安全性评估检查（血常规、尿常规、血生化）；
5. 在计划的前 6 周研究用药结束后[即研究第 7 周]进行首次疗效评估，后续每 8 周进行 1 次评估，其中，前 6 周给药研究结束后疗效评估时间窗为±3 天，后续评估时间窗为±7 天；
6. 生存随访可通过电话问询方式进行；
7. 签署知情同意书并完成肿瘤确诊的受试者，应对肿瘤组织进行 RAS、BRAF 基因检测（由本研究指定的中心病理实验室完成）；
8. 体重在基线记录，用于研究药物剂量计算。后续研究进行中，每次用药前进行称重，当体重变化与当前用药参考使用体重≤10%，无需进行剂量调整；
9. 实验室检查血样采集前需空腹；
10. 免疫原性血样采集：
    - 血样采集时间点：受试者应分别于基线、开始研究治疗后肿瘤疗效评估时间点（第 7 周，及之后每 8 周±7 天）进行血样采集，采集时间为给药前；
    - 提前退出研究受试者需在研究提前退出访视时采集该血样；

- 进入研究随访期的受试者需在安全性随访访视中采集该血样。

11. 探索性研究样本采集：
    - 肿瘤标本：可来自筛选期 RAS/BRAF 基因野生型确认分析所用相同或剩余的样本。具体要求按中心病理实验室手册操作指南；
    - ctDNA 血样采集时间点：应于筛选期、开始研究治疗后肿瘤疗效评估时间点（第 7 周，及之后每 8 周±7 天）、以及肿瘤进展时获取血液标本进行 KRAS、NRAS、BRAF、PIK3CA、EGFR 等热点基因动态监测。基线和肿瘤疗效评估时间点的血样采集时间与免疫原性研究相同，采集时间均为给药前。具体要求按中心病理实验室手册操作指南。
12. 妊娠试验：对于非绝经后或未接受过手术绝育术的女性，研究筛选期应进行血清 β-人绒毛膜促性腺激素（β-hCG）妊娠检查，入组后可接受尿妊娠结果（如安全访视检查）；
13. 肿瘤评估：筛选期与整个研究期间接受的肿瘤/疗效评估（评估范围含胸、腹或盆腔）方法应保持一致，且均应完成中心影像评估。如受试者参加研究提前退出访视，且自最近一次方案规定的疗效评估已≥4 周、尚未开始后续针对 mCRC 的抗肿瘤治疗，应尽可能在离开研究前完成疗效评估。首次出现疗效缓解需在 4 周后进行疗效确认影像学检查；
14. SCT200 静脉给药：每周给药阶段，建议每周第 1 天给药，研究用药时间窗为±2 天。每 2 周给药阶段，建议与每周给药时间点保持平行（如，每周给药周三进行，每 2 周给药在第一周的周三进行），研究用药时间窗为±3 天，直到出现不可耐受的研究用药相关 AE、PD、开始后续抗肿瘤治疗、死亡或失访。原则上应保证相邻两次研究用药间隔 7/14 天，如因 AE 或其他原因导致研究用药错后/超窗，建议后续研究用药以调整后的每周固定天给药，具体规定见方案第 5 章节描述；
15. 疾病进展随访、生存随访，仅收集研究药物相关不良事件。

1. 研究背景

1.1 前言

1.1.1 结直肠癌流行病学及临床治疗现况

结直肠癌是最常见的消化道恶性肿瘤之一，全球调研数据显示其发病率和死亡率均居恶性肿瘤的第三位[2]，2000年我国约有14.6万结直肠癌新发病例，7.87万受试者死亡，占癌症死因的第5位[3]，且近年来发病率明显上升[4]。由于结直肠癌诊断手段、手术技术及支持治疗的不断进步，结直肠癌死亡率逐渐下降，但20%左右受试者在诊断时已发生转移，其5年生存率较低[5]。

对于晚期结直肠癌，以全身化疗、局部放疗、同步或序贯放化疗为主的治疗缺少特异性，在杀伤肿瘤细胞的同时也对人体正常细胞产生杀伤作用，且传统细胞毒化疗药物进一步提高抗恶性肿瘤临床疗效的空间十分有限，因此，特异性强、毒副作用相对小的肿瘤分子靶向治疗逐渐成为继手术、放疗和化疗三大常规治疗手段的第四种治疗模式。其中，表皮生长因子受体(epidermal growth factor receptor，EGFR)是多种肿瘤治疗的明确靶点。EGFR是由c-ErbB1基因编码、有酪氨酸蛋白激酶活性的跨膜受体，通过介导多种途径信号通路调控细胞生长、分化、增殖、粘附、迁移和存活。EGFR普遍表达于人体表皮细胞和基质细胞，在多种人类恶性实体瘤中存在过表达或突变，其中在大肠癌的表达率为60％～80％[6]，过表达或突变常与预后不良、转移快、短期复发和生存期短等相关。

抗EGFR抗体可竞争性阻断其内源性配体EGF、TGF-α与EGFR的结合，促进EGFR的降解，使EGFR表达下调，进而阻断其酪氨酸激酶磷酸化以及胞内信号转导途径，最终抑制下游信号传导级联过程[7]，抑制癌细胞增殖并诱导其凋亡，还可减少基质金属蛋白酶和血管内皮生长因子(VEGF)的产生[8]，最终抑制肿瘤细胞的增殖和转移。抗EGFR单克隆抗体分子靶向治疗已成功应用于临床抗肿瘤治疗，成为恶性肿瘤患者一新的治疗选择。

1.1.2 EGFR 单抗药物简介

西妥昔单抗（通用名：Cetuximab；商品名：Erbitux [爱必妥]）是一种重组人/鼠嵌合型抗EGFR IgG$_1$单克隆抗体(monoclonal antibody，mAb)，FDA于2004年批准

其用于治疗转移性结直肠癌。2006年经CFDA批准，在中国上市，适应症为转移性结直肠癌，同年FDA批准其用于头颈部鳞癌适应症。

在西妥昔单抗联合最佳支持治疗vs.最佳支持疗法三线治疗mCRC研究中，中位OS在两组分别为6.1个月和4.6个月；ORR在西妥昔单抗联合最佳支持治疗组为8%，而对照组为0；6个月PFS在两组分别为15.0%和3.0%。西妥昔单抗临床推荐剂量为首次剂量400 mg/m^2/周，维持剂量250 mg/m^2/周。

帕尼单抗（通用名：Panitumumab；商品名：Vectibix）是一个全人源IgG2型单抗，被美国FDA和欧洲EMEA批准用于单药治疗氟尿嘧啶、奥沙利铂或伊立替康治疗无效、EGFR呈阳性表达、KRAS基因呈野生型的mCRC。2014年5月23日，美国FDA又批准帕尼单抗联合FOLFOX（一种基于奥沙利铂的化疗方案），用于野生型KRAS的转移性结直肠癌患者的一线治疗。帕尼单抗临床使用推荐剂量为6 mg/kg，每2周一次静脉输注。

帕尼单抗与西妥昔单抗曾针对化疗失败、KRAS野生型mCRC患者人群进行了头对头临床研究（ASPECCT），研究结果为：中位OS在帕尼单抗组为10.4个月，西妥昔单抗组为10.0个月；中位PFS分别为4.1个月和4.4个月；ORR分别为22%和19%。与西妥昔单抗治疗mCRC相似，在KRAS突变mCRC患者中帕尼单抗疗效不佳。

在结直肠癌患者中，肿瘤原发部位在左半结直肠和右半结直肠的比例大致为2:1，且其分子生物学及临床特征明显不同。基于6项随机临床研究结果的回顾性分析提示[9]，在RAS野生型mCRC患者中，OS、PFS及ORR在右半结肠癌患者较左半结肠癌患者差。另有研究结果[10]提示，在采用西妥昔单抗、帕尼单抗或西妥昔单抗/伊立替康治疗的75名mCRC患者中，原发部位在左半结直肠较右半结直肠的治疗反应率高（41%vs.0），中位PFS明显延长（6.6个月 vs. 2.3个月）。此外，野生型BRAF也是西妥昔单抗和帕尼单抗治疗转移性结直肠癌的疗效预测因子。有研究报道BRAF点突变的mCRC患者较BRAF野生型患者的PFS和OS短。

综上，EGFR单抗治疗结直肠癌的效果与RAS基因突变情况和结直肠癌的原发部位有关，对于KRAS 野生型及肿瘤原发部位在左半结直肠的患者疗效优于KRAS突变型及右半结直肠癌患者，故RAS及BRAF基因的突变状态和结直肠癌原发部位被认为是EGFR单抗治疗结直肠癌的疗效预测因子。

1.1.3 SCT200 介绍

神州细胞工程有限公司研制的重组全人源抗人EGFR单克隆抗体（SCT200）是一由1326个氨基酸组成、分子量约为145 kDa的IgG_1 κ型单克隆抗体，能与人表皮生长因子受体特异性结合，临床拟用于转移性结直肠癌的治疗。SCT200临床前药理研究结果表明，抗体亲和力高（Kd 0.08 nM），能与人表皮生长因子受体发生特异性结合，阻断EGF等配体与受体的结合从而抑制受体激活和EGFR下游信号转导。体外研究显示SCT200对多种EGFR阳性表达肿瘤细胞具有增殖抑制作用（MDA-MB-468、A431、FaDu、SW948、PC-3）；不同于帕尼单抗，作为一IgG_1型单抗，SCT200可通过Fc功能区发挥CDC、ADCC效应杀伤肿瘤细胞。SCT200单用可显著抑制表皮鳞癌A431细胞及结肠癌SW948细胞生长；与化药联合使用显示出一定相加作用。

SCT200在相关动物种属食蟹猴模型中完成了一般药理学、急性毒性实验、长期毒性试验、溶血性及局部刺激试验以及免疫原性实验。

SCT200每周静脉注射3、7.5、15 mg/kg，连续给药26周的长期毒性反应主要集中在皮肤和胃肠道系统。皮肤毒性包括发红、干燥、脱屑、溃疡、有渗出物、瘙痒、脱毛，皮肤感染等，其发生率和严重程度呈剂量相关性，停药后有所缓解和恢复。SCT200给药期间，各剂量组均有动物出现包括软便、稀便、水便等粪便异常以及胃肠道不适造成的动物食物摄取量降低、食欲不振、体重下降。研究中未发现SCT200的无明显毒副反应剂量(NOAEL)。尽管上述毒性病变发生率和严重程度较高，但其与抗EGFR抗体的药理学活性及作用机制有关，与临床安全性有很好相关性。

参考同类机制药物临床安全性研究数据可知，上述不良事件通过预防和对症处理可有效控制。作为一全人源IgG_1型抗EGFR抗体，SCT200的抗原结合表位及其理化特性、生物活性与已上市药物有所不同，其临床有效性是值得期待的，且SCT200临床应用的免疫原性将大大降低，更适合恶性肿瘤受试者的长期治疗。

1.2 SCT200 I 期临床研究简介

1.2.1 研究设计

SCT200 I期研究主要目的是评估SCT200静脉单次给药和多次给药在经氟尿嘧啶，奥沙利铂和伊立替康治疗失败的转移性结直肠癌患者中的安全性和耐受性；以

及评估SCT200静脉单次给药和多次给药在经氟尿嘧啶，奥沙利铂和伊立替康治疗失败的转移性结直肠癌患者中的药代动力学。

次要目的是初步评价SCT200临床疗效，同时为本品II期临床试验剂量选择提供依据。

本研究采用单中心、非随机、开放、剂量递增的 I 期临床试验设计，选择了经氟尿嘧啶，奥沙利铂和伊立替康治疗失败的转移性结直肠癌患者作为研究人群。试验设定单次给药试验和多次给药试验，分为 0.5mg/kg、1.0mg/kg、2.0mg/kg、4.0mg/kg、6.0mg/kg 和 8.0mg/kg 六个递增剂量组。完成单次给药耐受性、安全性评估的受试者在经过 3 周洗脱期后，可在原剂量水平继续进入多次给药试验。连续多次给药试验的起始剂量为 0.5mg/kg，各剂量组与单次给药试验相同，将依次逐级爬升，直至最高 8.0mg/kg。给药方式，0.5mg/kg 剂量组至 2.0mg/kg 剂量组接受每周一次连续四次的静脉给药，4.0mg/kg 剂量组接受每周一次连续六次的静脉给药，8.0mg/kg 剂量组接受每二周一次连续三次的静脉给药。6.0mg/kg 剂量组给药方案将根据单次给药药代分析结果，以及前面剂量组的连续多次给药药代结果，决定采用每周一次连续六次的静脉给药（QW，6dose），或/和每二周一次连续三次的静脉给药方案（Q2W，3dose）。完成 6 周研究治疗后至少获得疾病稳定疗效的受试者，进入维持治疗阶段至治疗 12 个月或疾病进展（PD）或出现不可耐受毒性。

剂量爬坡试验结束后，如果确定目标剂量，允许在目标剂量进行增加样本量的放大研究。

1.2.2 研究结果

1.2.2.1 试验总体进展及入组情况

因 I 期研究尚未结束，此方案中给出数据为初步结果。

剂量爬坡研究最终完成了七个组别剂量递增，共入组 22 例，确定了目标剂量为 6.0mg/kg, QW (具体确定依据见药代动力学结果) ，正在此剂量组扩大研究中。

剂量爬坡研究各剂量组入组例数，除了 4.0mg/kg，因 1 例患者在单次给药后疾病进展只完成了单次给药之外，其余剂量组的 3 例患者均完成单次给药和多次给药。多次给药阶段，0.5mg/kg, 1.0mg/kg 和 2.0mg/kg 三个剂量组治疗阶段完成 4 次给药，

6.0mg/kg 完成两种给药方式，每周给药（给药 6 次）和每两周给药（给药 3 次），8.0mg/kg 为每两周给药方式，完成 3 次给药治疗期。 所有治疗期结束后，肿瘤评估达到至少疾病稳定可继续进入维持治疗，结果显示，从 2.0mg/kg 剂量组开始已经有患者进入维持治疗。

扩大入组研究，按照 6.0mg/kg, 每周给药的给药方式，正在入组研究中。给药依然遵循剂量爬坡研究的治疗方案， 每周给药， 完成 6 次给药后，如果肿瘤评估达到至少疾病稳定可继续进入维持治疗，直至疾病进展或不可耐受毒性或死亡。

本方案中的 I 期结果为初步结果，基于现有数据分析，安全性数据基于剂量爬坡研究的 22 例和扩大入组研究的 13 例数据进行分析；有效性结果基于 37 例的数据进行分析，包括剂量爬坡研究的 22 例和扩大入组研究的 15 例数据进行分析。

1.2.2.2 安全性结果

基于现有的初步安全性数据显示， SCT200 在晚期结直肠癌患者中的安全性和耐受性良好。在剂量爬坡研究中（N=22）未见 DLT, 未见药物相关的 SAE。100%患者发生了不良事件（AE），其中与药物相关的 AE 发生率为 90.9%（20/22），有 5 例患者因 AE 而剂量递减或者暂停用药为 22.7%（5/22）。扩大入组研究中（N=13），与药物相关的 AE 发生率为 100%（13/13），其中 6 例因 AE 暂停用药或者剂量递减。

与药物有关的特殊关注的 AE，包括皮肤毒，低镁血症及输液反应情况，基于目前接受过 SCT200 治疗的 35 例患者（包括剂量爬坡研究 22 例和扩大入组研究 13 例），本研究者发现 SCT200 的皮肤毒发生率与帕尼单抗和爱必妥相当，但是严重程度， 即 3 级以上的皮疹发生率低于另外两个同类品种；低镁血症发生率高于帕尼单抗和爱必妥，以 1 级低镁血症为主；输液反应发生率与帕尼单抗类似，均低于爱必妥，仅在扩大入组研究中发生 1 例输液反应，级别是 1 级输液反应，发生时间是在输注结束后 1 小时内。尚未发现腹泻脱水等症状，亦未发现间质性肺疾病的案例。因试验尚未结束，最终数据暂时不能给出。

免疫原性情况，35 例患者中有 1 例发生抗抗体阳性（2.9%），在 0.5mg/kg 剂量组单次给药后 21 天的抗抗体阳性。

至 2017.11.09 日，共发生 3 例 SAE。其中 1 例患者发生右侧输尿管结石，SAE 与研究药物无关。另外 1 例为急性髓性白血病，判断为与既往化疗有关，与研究药

物无关。第三例 SAE 为消化道出血，因肿瘤进展累及胃壁致血管破裂可能性大，加之既往多次行肝内放射性粒子植入术，导致胃黏膜持续放射性损伤存在，经判断与研究药物可能无关。至目前，急性髓性白血病和消化道出血患者已经死亡。

1.2.2.3 有效性结果

共 37 例受试者接受了研究用药后有效性评估，其中剂量爬坡阶段研究共 22 例，扩大入组研究 15 例。截止 2017 年 11 月 09 日，爬坡阶段研究受试者中，8 例受试者出现 SD (仅 1 例为 2.0mg/kg/QW 以下治疗组)， 1 例出现 PR（6.0mg/kg/Q2W 组）。此 9 例受试者进入维持治疗期，其中 7 例已出现 PD，2 例仍在接受维持治疗。在扩大入组研究中，已有 15 例受试者获得疗效评估数据，ORR 为 73.3%（11/15），PFS>24 周，疾病控制率（DCR）达 100%。目前入组受试者进入维持期，总 PR 率（9+15）：54.1%（13/24），PFS＞24 周。

上述数据初步提示 SCT200 在经氟尿嘧啶、奥沙利铂和伊立替康治疗失败的 RAS 野生型转移性结直肠癌受试者中安全性和耐受性可控，疗效显著。

1.2.2.4 药代动力学（PK）结果

<u>单次给药药代动力学研究</u>：SCT200 单次给药PK研究是在 0.5mg/kg、1.0mg/kg、2.0mg/kg、4.0mg/kg、6.0mg/kg和 8.0mg/kg六个剂量水平完成评估。每个剂量组在完成单次给药，经过 3 周洗脱期后可在原剂量水平继续进入多次给药部分。研究结果显示，单次静脉输注SCT200 后，低剂量组SCT200（0.5mg/kg-2.0mg/kg）呈现快速靶向清除，一周后血药浓度已检测不到或接近检测下限，而 4.0mg/kg剂量组及以上的高剂量组（6.0mg/kg-8.0mg/kg），其体内靶向清除则呈现逐渐趋于饱和趋势。在 0.5mg/kg-8.0mg/kg剂量范围，药物呈现非线性药代动力学特征。随着剂量的增加，AUC暴露量亦增加，清除率由 2.33mL/h/kg降至约 0.30 mL/h/kg， $T_{1/2}$也由 17.66 h 延长至 97.97 h。4.0mg/kg，6.0mg/k和 8.0mg/kg剂量组单次给药后的峰浓度C_{max} 分别为 74.98ug/mL, 109.50 ug/mL和 190.97ug/mL。与同靶点帕尼单抗相比，相同剂量SCT200 其体内暴露量低于帕尼。

<u>多次给药的药代动力学研究</u>：0.5mg/kg、1.0mg/kg、2.0mg/kg 3 个剂量组接受QW、连续四次的静脉给药。根据单次给药PK结果，试验过程中 4.0mg/kg剂量组给药方案调整为QW、连续六次静脉给药；6.0mg/kg剂量组则完成两种给药方式，即

QW、连续六次的静脉给药和Q2W、连续三次的静脉给药；8 mg/kg剂量组亦接受Q2W、连续三次的静脉给药。多次给药的初步结果显示，SCT200 每周给药剂量组4.0mg/kg 和 6.0mg/kg达稳态后，其谷浓度分别为 43.9 ug/mL和 92.6 ug/mL，每两周给药剂量组 6.0mg/kg 和 8.0mg/kg 第三次给药的谷浓度分别为 29.1 ug/mL和 58.1 ug/mL。爱必妥和帕尼单抗的多次给药的稳态谷浓度分别为 54ug/mL和 50ug/mL。参考同靶点药临床应用稳态后的PK参数，由此可推测SCT200 4.0mg/kg、6.0mg/kg每周给药，连续给药六次，以及按 8.0mg/kg剂量每两周给药频率，连续给药 3 次，其谷浓度应可达到饱和稳态药理活性平台。

综合 SCT200 I 期各剂量组靶点相关的皮肤毒及临床疗效数据，推荐后续的 II 期给药方案为 6.0mg/kg/QW，连续给药六周；之后进行肿瘤疗效评估，对于稳定及缓解的患者后续给药方案调整为 8 mg/kg/Q2W，持续给药直至肿瘤进展或不能耐受的毒性反应。

2. 研究目的

2.1 主要目的

本研究的主要目的是评估 SCT200 静脉给药在经氟尿嘧啶、奥沙利铂和伊立替康治疗失败的 RAS/BRAF 野生型转移性结直肠癌受试者中的有效性。

2.2 次要目的

1) 评估 SCT200 静脉给药在经氟尿嘧啶、奥沙利铂和伊立替康治疗失败的转移性结直肠癌受试者中的安全性；
2) 评估 SCT200 静脉给药在经氟尿嘧啶、奥沙利铂和伊立替康治疗失败的转移性结直肠癌受试者中的免疫原性。

3. 研究设计

3.1 研究设计原理

已有研究数据及临床治疗情况均提示，抗 EGFR 单抗靶向治疗已经成为晚期转移性结直肠癌患者的一种新的选择方式，已上市的西妥昔单抗和帕尼单抗分别获准联合化疗一线/二线或单药治疗 KRAS 基因野生型的 mCRC。

已完成的 SCT200 I 期临床研究受试者人群为经氟尿嘧啶、奥沙利铂和伊立替康治疗失败的 RAS 野生型转移性结直肠癌患者，该研究评估了 SCT200 静脉单次给药和多次给药在上述患者人群中的安全性、耐受性和药代动力学。安全性结果提示 SCT200 在剂量爬坡阶段未见 DLT。未见研究药物相关 SAE；SCT200 的总体 AE 发生情况与帕尼单抗、西妥昔单抗既往研究安全性数据相似，其中 SCT200 的皮肤毒性可能略低于帕尼单抗和西妥昔单抗，3 级皮肤毒性反应发生率为 11%（帕尼单抗、西妥昔单抗相应发生率为 16%和 12%）。低镁血症总体发生率较帕尼单抗、西妥昔单抗高，但严重程度主要为 1-2 级，对症处理后均痊愈。扩展阶段有效性结果初步提示，SCT200 在目标患者人群中的疗效可能优于同类抗体药物，综合 I 期研究安全性和有效性结果，初步提示 SCT200 对于 RAS 野生型转移性结直肠癌受试者人群疗效显著，同时安全风险可控，但需进一步扩大样本量以证明其对上述患者人群的临床获益。

本研究设计用以确认 SCT200 在经氟尿嘧啶、奥沙利铂和伊立替康治疗失败的 RAS/BRAF 野生型转移性结直肠癌受试者中的有效性和安全性，以支持在中国的注册批准。研究主要终点客观缓解率可直接反应研究药物的抗肿瘤活性。同时，设有一系列次要疗效终点如疾病控制率（DCR）、缓解持续时间（DOR）、至缓解时间（TTR）、无进展生存期（PFS）等，以证明研究药物对于中国目标适应症患者人群的治疗获益。此外，安全性评估终点包括了此类单抗药物常见 AE 包括皮肤毒性、低镁血症等，从而在相对较大规模研究患者人群中进一步评估 SCT200 的安全性。

3.2 风险/获益评估

临床前药理、毒理和药效学研究以及已完成的 I 期临床研究结果均已提示，SCT200 用于经氟尿嘧啶、奥沙利铂和伊立替康治疗失败的转移性结直肠癌受试者的抗肿瘤治疗具有积极的获益。此外，同类作用机制的进口药物对于上述患者人群的临床获益已得到充分证实，临床用药实践表明其在提高患者 ORR 及延长 PFS 方面疗效显著，同时患者出现不良事件相对传统化疗少，但药物临床使用价格相对昂贵。

根据既有临床前、临床研究数据及同类药物上市使用情况分析，依据本方案规定的 SCT200 给药方案开展研究是科学合理的，对于经氟尿嘧啶，奥沙利铂和伊立

替康治疗失败的 RAS/BRAF 野生型转移性结直肠癌患者的治疗可能带来临床获益，同时风险可控。

3.3 研究设计

3.3.1 研究总体设计

本研究为一项单臂、多中心的II期临床研究，以评估SCT200在经氟尿嘧啶、奥沙利铂和伊立替康治疗失败的RAS/BRAF野生型转移性结直肠癌受试者中的有效性和安全性。本研究计划入组110例受试者，试验包括3个阶段：筛选期、治疗期和随访期。

3.3.1.1 筛选期：

受试者签署知情同意书后，进入研究筛选期。根据获得实验室检查结果的时限，筛选期最长28天，期间受试者将按照**表1**中的访视计划，完成筛选期评估。符合研究所有入选标准且不符合排除标准的受试者将进入研究治疗期。

3.3.1.2 治疗期：

进入治疗期的受试者将接受SCT200 6.0mg/kg，连续 6周每周给药1次，之后接受8.0mg/kg，每2周给药1次，持续给药治疗。SCT200静脉输注给药，每位受试者用药量将根据其体重计算得出。输注前至输注结束后1小时内，需严密监测受试者的各项生命体征。输注过程中需密切观察受试者面色、是否有出汗或头痛等，以便及时发现输注反应相关临床表现。受试者接受研究用药治疗后，按访视计划行疗效评估和安全性检查。本研究筛选期及整个研究期间的肿瘤评估，将依据实体瘤疗效评价标准（RECIST版本1.1）。应尽可能采用增强的计算机断层扫描（CT），如受试者对造影剂过敏，可替换为磁共振成像（MRI））检查进行疗效评估，每位受试者的检查方法在研究期间应保持一致。

如疗效评估为疾病稳定、部分缓解或更好疗效，可允许继续治疗直至出现PD或不可耐受的毒性、死亡或开始后续抗肿瘤治疗。在治疗期间，如出现研究药物相关不良事件，研究者应对受试者风险/利益进行临床综合评估后，根据方案第5.4章节规定的研究药物剂量调整说明对受试者在后续治疗期间的研究用药剂量/停用药物作出决定。在治疗期间，受试者应根据方案访视操作流程，接受安全性评估（每

4周1次）及疗效评估（在结束每周给药方案即研究第7周时进行首次疗效评估，后续每8周进行1次疗效评估）直至发生PD、出现不可耐受的研究用药相关不良事件、开始后续抗肿瘤治疗、死亡或失访。

3.3.1.3 随访期：

受试者在结束研究治疗后进入随访期，随访期包括安全性随访、疾病进展随访和生存情况随访。所有至少接受过一剂研究治疗的受试者均需进行安全性随访访视，访视时间为最后一剂研究用药后 28（+7）天，期间需按访视流程进行评估。

在安全性随访后，对于尚未发生 PD 且未开始后续抗肿瘤治疗的受试者，将继续每 8 周进行 1 次疗效评估，其操作流程与研究治疗期疗效评估相同，直至发生 PD、开始后续抗肿瘤治疗、死亡或失访。

在安全性随访访视和/或疾病进展随访访视后，将根据标准的临床治疗进行生存情况随访。所有至少接受过一次 SCT200 治疗的受试者均需进行生存情况随访，这些访视计划在安全性随访/疾病进展随访访视后每 3 个月（±14 天）1 次，可通过电话问询方式进行，以收集安全性访视后开始的后续抗肿瘤治疗以及生存状况信息。

研究结束：定义为直至至少 85%受试者出现疾病进展、开始后续抗肿瘤治疗、死亡、提前退出研究或失访。此时仍在接受研究用药的受试者将由研究者基于临床综合判断决定其后续适当的临床治疗/处理。如研究者认为受试者继续用药仍将有临床获益，可继续给予受试者临床研究用药。

3.3.2 中心疗效评估委员会（IRC）

本研究将按照中心疗效评估委员会章程所述，依据实体瘤疗效评价标准（RECIST）版本 1.1（附件四）对所有受试者的影像学数据进行独立审核。

所有的肿瘤评估影像图像将被送至独立的中心影像实验室进行独立的中心影像阅片。具体细节将在影像手册及独立影像阅片章程中描述。IRC 将报告肿瘤负荷描述、测量结果及最终治疗疗效评估结果。此外，研究者也将根据 RECIST 版本 1.1 完成疗效评估，其结果将在 CRF 上记录。

3.3.3 研究终点

<u>主要终点</u>：结直肠癌受试者总体人群中的客观缓解率（ORR）。

次要终点：

- 次要疗效终点：左半结直肠癌受试者人群中的 ORR、最佳疗效（BOR），疾病控制率（DCR），缓解持续时间（DOR），至缓解时间（TTR），无进展生存期(PFS)，PFS 率(6 个月、9 个月和 12 个月)，总生存期(OS)。

上述疗效终点中的肿瘤治疗反应均依据实体瘤疗效评估标准（RECIST）版本 1.1 由中心审阅进行评估。首次出现疗效缓解需在 4 周后进行疗效确认影像学检查。

左/右半结肠定义[1]：肿瘤原发部位位于右半结肠包括盲肠、升结肠和近端 2/3 的横结肠，左半结肠包括远端 1/3 的横结肠、降结肠、乙状结肠和直肠。

- 安全性评估终点：TEAE、临床实验室检查值、生命体征、12-ECG、免疫原性，体格检查（包括皮肤毒性反应）。

4. 研究人群

4.1 受试者

4.1.1 入选标准

符合下列所有标准的受试者可入选本研究：

1) 自愿参加本项临床试验并签署知情同意书；
2) 年满 18 周岁，性别不限；
3) 预计生存期≥3 个月；
4) ECOG 体力状况评分 0～1 级；
5) 经病理学检查确诊为转移性结直肠腺癌；
6) 受试者既往接受过标准化疗方案治疗并失败。这些标准治疗方案中必须包含氟尿嘧啶类、奥沙利铂、伊立替康。治疗失败的定义为：治疗过程中或末次治疗后的 6 个月内出现疾病进展或毒副作用不可耐受。注：
    a) 标准治疗方案治疗以至疾病进展为止，用药时间≥1 个周期的一种或多种化疗药物；
    b) 允许前期进行辅助/新辅助治疗。如果辅助/新辅助治疗期间或者完成后 6 个月内出现复发或转移，则认为辅助/新辅助治疗是一个针对进展期疾病的一线前期全身化疗的失败 ；

7) 肿瘤状态为 RAS 和 BRAF 基因野生型；

8) 实验室检查：

   - 血常规：中性粒细胞≥1.5×l0^{9}/L，血小板≥75×10^{9}/L，血红蛋白≥80g/L；
   - 肝功能：谷丙转氨酶（ALT）和谷草转氨酶（AST），无肝转移者 ALT 和 AST≤正常值上限×3，有肝转移者 ALT 和 AST≤正常值上限×5；总胆红素(TBIL)≤正常值上限×1.5；
   - 肾功能：肌酐(Cr)≤正常值上限×1.5；
   - 电解质：镁≥正常下限；

9) 根据 RECIST 标准 1.1 版，至少有一个经 CT 或 MRI 检查显示最长径≥10mm（扫描厚度不超过 5.0mm），淋巴结：≥15mm，可测量的肿瘤病灶（非放射治疗野）；

4.1.2 排除标准：

符合以下任一标准的受试者应从本研究中排除：

1) 筛选前已知有中枢神经系统转移或有中枢神经系统转移病史的患者。对于临床疑似中枢神经系统转移的患者，随机化前 28 天内必须进行影像学确认，排除中枢神经系统转移；

2) 有其他恶性肿瘤病史，除外：在入组前 5 年或 5 年以上恶性病灶已经过治疗性措施的处理且不存在已知的活跃病灶，经主治医师判断复发风险较低；接受充分治疗的非黑色素瘤皮肤癌，且无病情恶化证据；接受充分治疗的原位宫颈癌，且无病情恶化证据；前列腺上皮内瘤，无前列腺癌复发证据；

3) 已知对试验药物中抗体或所含其他成分过敏者；

4) 曾接受 EGFR 抗体（如，帕尼单抗、西妥昔单抗或其类似物），或小分子 EGFR 抑制剂（如，吉非替尼、埃罗替尼、拉帕替尼等）；

5) 在入组前 4 周或 4 周内，接受过抗肿瘤药物治疗（如，化疗、激素治疗、免疫治疗、抗体治疗、放疗）或接受研究药物治疗，或入选时受试者仍存在之前抗肿瘤治疗导致的≥2 级毒副反应（除外脱发和奥沙利铂引起的≤2 级神经毒性）；

6) 受试者当前已入组至其他研究器械或研究药物研究中，或距离其他研究药物或研究器械停用时间少于或等于 4 周；
7) 在入组前 4 周或 4 周内接受过大手术（如需要全身麻醉），受试者须从与手术相关的损伤中恢复；
8) 入组前 2 周内接受过输血、促红细胞生成素（EPO）、粒细胞集落刺激因子（G-CSF）或粒细胞-巨噬细胞集落刺激因子（GM-CSF）的治疗；
9) 有临床意义的心血管疾病（即定义为：不稳定型心绞痛、有症状的充血性心力衰竭（纽约心脏病协会[NYHA]≥II 级）、不可控的严重心律失常）；
10) 在入组前 6 个月内发生过心肌梗死；
11) 有间质性肺病（ILD）病史，如间质性肺炎、肺纤维化，或在基线胸部 CT 或 MRI 上显示有 ILD 证据；
12) 有临床症状，需要临床干预或稳定时间小于 4 周的浆膜腔积液（如胸腔积液和腹水）；
13) 可能干扰结果解释的医疗或精神病史或实验室异常病史；
14) 妊娠期或哺乳期的受试者，或计划在治疗期间及治疗结束后 6 个月内妊娠的受试者；
15) 在治疗期间与治疗结束后 6 个月内，不愿意接受有效避孕措施的受试者(包括男性或女性受试者）；
16) 患者处于乙肝或丙肝活动期等（既往有乙肝感染病史，无论是否服用药物控制，HBV DNA≥10^4拷贝数或者≥2000IU/ml；丙肝感染，HCV RNA≥15IU/ml)；人免疫缺陷病毒（HIV）抗体阳性（如无临床证据提示可能存在HIV感染时，无需检测)；
17) 在入组前 2 周或 2 周内，受试者存在需要系统治疗的活动性感染或不可控感染（简单泌尿道感染或上呼吸道感染除外）；
18) 已知受试者存在酒精或药物成瘾；
19) 研究者认为受试者存在可能影响其对方案依从性和研究指标评估的其他状况，不适宜参加研究的受试者。

4.2 提前退出研究

4.2.1 研究者决定的提前退出

如果在本研究过程中发生以下情况之一，研究者应当安排其中断研究治疗并退出研究。退出研究前，研究者应尽可能完成方案规定的研究提前退出访视评估流程。

- 提示有肿瘤疾病进展的证据；
- 发生任一 AE，研究者认为受试者不适合继续参加本研究；
- 任何原因导致的连续暂停 SCT200 给药＞2 次；
- 由于受试者合并疾病或健康状况的改变，研究者认为受试者不适合继续接受研究治疗；
- 怀孕或哺乳；
- 受试者严重不依从方案规定的研究流程包括研究治疗；研究者基于受试者临床情况综合判断，认为受试者需接受其他抗肿瘤药物治疗以最大获益。

4.2.2 受试者自行退出研究

受试者在研究过程中任何时候均可退出研究（包括停用研究药物以及相关研究评估），且不会影响其后续治疗。研究者应尽可能了解受试者退出研究的原因及是否发生任何不良事件。如可能，研究者应面见受试者，通知其尽可能按方案要求完成提前退出研究访视流程，并通知申办方。应对不良事件进行随访。

4.3 试验终止

在所有的受试者完成治疗和评估，并且数据已经锁定后，试验中心会被关闭。除此之外，研究也可能会因为申办方、研究者或者法规当局的要求而终止，任何一方提出终止研究的要求，必须及时且正确地告知其他方，共同协商后可以提前关闭试验中心。如果需要终止或暂停试验，研究者应该按照有关法规要求告知伦理委员会终止或暂停试验的原因。

如果出现以下情况，则应考虑终止本试验：

- ✓ 研究者发现有严重安全性问题；
- ✓ 疗效无法达到预期，无继续进行临床试验的必要；
- ✓ 试验方案有重大失误；

- ✓ 申办者因经费或管理原因；
- ✓ 行政主管部门撤销研究；
- ✓ 如果出现以下情况，申办方有权决定终止某个试验中心的试验：
  - 试验中心无法在约定的时间完成约定的入组人数；
  - 试验中心严重违背 GCP；
  - 试验中心严重违背方案；

终止研究后，全部试验相关记录应予保留备查。

5. 研究药物

5.1 研究药物基本信息

药物名称：重组全人源抗人表皮生长因子受体单克隆抗体注射液

英文名称：Recombinant Humanized Anti-Human Epidermal Growth Factor Receptor Monoclonal Antibody Injection

研究药物编号：SCT200

研究药物具体信息：

成份：抗体浓度 10mg/ml，每瓶 5 ml 含 50mg 重组全人源抗人表皮生长因子受体单克隆抗体及其它组分：氯化钠、组氨酸、冰醋酸、聚山梨酸酯和注射用水。

性状：重组全人源抗 EGFR 单克隆抗体注射液为不含防腐剂的无菌、无色液体制剂，微带乳光。

规格：50 mg(5ml)/瓶

有效期：36 个月

贮藏：2～8℃储存，避光，不得冷冻。配制好的注射液在 2～8℃可保持稳定 24 小时，室温下可保存 12 小时。

5.2 包装和标签

本试验药物为西林瓶内包装，纸盒外包装。每小盒 1 支，每中盒 10 支，中盒内附有药物使用说明书。小盒和中盒包装的标签上印有药物名称、规格、使用方法、贮藏、生产批号、生产日期、有效期、生产企业等信息，并注明“仅供临床研究使

用”字样。

5.3 药物配制和给药

将试验药物从冰箱2～8℃的冷藏室中取出，在无菌条件下抽取所需剂量的重组全人源抗人EGFR单克隆抗体注射液（SCT200），用0.9%无菌注射生理盐水稀释至终体积100 ml，静脉输注给药。药物应在稀释后立即使用。如没有立即使用，应在2～8℃冷藏，且贮藏时间不超过12小时；如室温保存，应在6小时内使用完毕。稀释后的溶液不能冷冻。本品无需预防用药，如果出现1级或2级输液反应，受试者在随后的输液前应给予预防性治疗，如给予抗组胺药物、皮质类固醇、对乙酰氨基酚等药物。研究药物在60分钟内完成输注，如果输注总量超过1000mg，则在90分钟内完成输注。输注过程中如出现输液反应按照本方案章节5.4.1描述处理。

本研究治疗阶段给药方案为：SCT200 6.0mg/kg，连续6周每周给药1次，之后接受8.0mg/kg，每2周给药1次，持续给药治疗。

5.4 研究用药剂量调整原则

5.4.1 输液反应

应考虑 4.03 版常见不良事件毒性反应标准（CTC-AE）中定义的输液相关反应、过敏反应/变态反应内容。

a 对于 1 级或 2 级反应，应将滴速下调约 50％；

b 对于 3 级或 4 级反应，考虑且永久性停止输液。

✓ 滴速调整后，研究者视受试者具体临床表现，判断是否暂停或进行滴速回调。在受试者后续研究中，原则上不受此次滴速调整影响，推荐正常滴速使用。

5.4.2 皮肤毒性

受试者在本研究中如发生 1 级或 2 级皮肤毒性反应时，应根据方案 5.8.3 章节规定接受相应临床处理。对于无法耐受方案规定的剂量的受试者，允许进行剂量调整，以便受试者继续接受研究治疗，必须在用药记录eCRF中记录SCT200 剂量变化。如受试者发生 3 级及以上程度皮肤毒性反应，研究用药具体剂量调整原则见下**表 2**。

表 2　发生 3 级及以上程度皮肤毒性反应时研究用药剂量调整原则

研究起始用药剂量	≥3 级皮肤毒反应	持续时间	后续研究用药日恢复情况	剂量调整方法
前 6 周<br>研究治疗<br>6.0mg/kg/QW	首次出现	延迟给药 1-2 周	恢复至≤2 级	维持原剂量（6.0mg/kg/QW）
			>2 级	停止研究给药
	第二次出现	延迟给药 1-2 周	恢复至≤2 级	剂量减低至 4.8mg/kg/QW<br>后续用药日如受试者未出现达到剂量调整的 AE，研究者可基于临床综合评估，将研究用药剂量回调。
			>2 级	停止研究给药
	第三次出现	延迟给药 1-2 周	恢复至≤2 级	剂量减低至 3.6mg/kg/QW
			>2 级	停止研究给药
	第四次出现	-	-	停止研究给药
后续研究治疗<br>8.0mg/kg/Q2W	首次出现	延迟给药 1-4 周	恢复至≤2 级	维持原剂量（8.0mg/kg/Q2W）
			>2 级	停止研究给药
	第二次出现	延迟给药 1-4 周	恢复至≤2 级	剂量降低至 6.4mg/kg/Q2W。<br>后续用药日如受试者未出现达到剂量调整的 AE，研究者可基于临床综合评估，将研究用药剂量回调。
			>2 级	停止研究给药
	第三次出现	延迟给药 1-4 周	恢复至≤2 级	剂量减低至 4.8mg/kg/Q2W
			>2 级	停止研究给药
	第四次出现	-	-	停止研究给药

5.5 暂停 SCT200 治疗标准

被视为与 SCT200 治疗有关的皮肤或指甲相关毒性：

- 所有 3 级或 4 级皮肤或指甲相关毒性；
- 需要静脉给予抗生素或抗真菌药物治疗的皮肤或指甲感染；
- 需要进行外科清创手术；
- 所有皮肤或指甲相关严重不良事件。

被视为与 SCT200 治疗有关的非皮肤或指甲相关毒性：

- 积极补充镁，但仍出现 3 级或 3 级以上低镁血症，则应停止 SCT200 治疗；
- 尽管给予对症处理，但仍持续出现 3 级或 4 级恶心、呕吐或腹泻，则应停止 SCT200 治疗；
- 不能通过治疗缓解的 3 级或 3 级以上贫血以及 4 级血小板减少症，应停止 SCT200 治疗；
- 除上述情况外的所有 3 级或 4 级毒性反应（除外脱发）。

5.6 重新开始 SCT200 治疗的标准

皮肤或指甲相关毒性：

当皮肤或指甲相关毒性反应恢复至满足下列所有标准时可重新开始 SCT200 治疗：

- 2 级或 2 级以下，或恢复至基线；
- 不再需要静脉给予抗生素或抗真菌药物治疗；
- 经外科清创术后已康复。

非皮肤或指甲相关毒性：

- 一旦不良事件改善为 2 级或 2 级以下，或返回至基线，则可开始给予 SCT200。

5.7 给药超窗及其它停用 SCT200 规定

每周给药阶段，每周的第 1 天（±2 天）给予 SCT200。每 2 周给药阶段，每 2 周的第一周第 1 天（± 3 天）给予 SCT200，建议与每周给药时间点保持平行。原则上应保证相邻两次研究用药间隔为 7/14 天，如因 AE 未恢复导致研究用药延后（如

从周一延后至周三用药），后续研究用药以调整后的固定天给药（如周三用药）。如果受试者因 AE 未恢复至可用药水平延迟给药超窗，受试者将在研究者判断其可再次用药时接受研究给药，在后续给药周应按调整后的用药日接受给药（如从周一调整至周四，后续在周四接受研究用药）。如因其他原因延迟给药超窗，研究用药日调整原则同上。

患者因毒性反应或其它原因而暂停使用 SCT200 连续 2 次以上，也将永久停用 SCT200。

5.8 研究用药注意事项

5.8.1 静脉输注期间的监护

本研究药物所有的给药操作应在研究者的指导下进行。在输注前、输注期间及输注结束后 1 小时，行心电监护并严密监测受试者的各项生命体征（体温、呼吸、血压和心率）以及面色、是否有出汗或头痛等，以便早期发现输注反应征象。部分输注反应可能发生于后续用药阶段，即使在首次使用后未出现任何级别的输注反应，后续的用药过程仍应在医生的监护下进行。

输注期间的监护时间点：

1) 在输注开始前；
2) 输注期间及输注结束后 1 小时：输注期间每半小时监测一次，输注结束后 1 小时监测一次。

5.8.2 输液反应的处理

研究过程中发生输液反应，剂量调整内容，请参考 5.4.1 章节内容。

对于发生输液反应处理，允许合并用药范围内，可依临床常规进行处理。处理过程完整记录于病历内，合并用药完整记录于病历及 CRF 相应页中。

输液反应处理推荐，详见**附件二**。

5.8.3 皮肤毒性处理

抗EGFR单抗药物最常见的不良事件是皮肤毒性，主要表现为痤疮样皮疹。出现皮肤毒性后，要正确解释皮疹严重程度与治疗获益的关系，增强受试者正确应对

皮肤不良事件的信心，嘱咐受试者注意常规皮肤护理外，可根据4.03版NCI-CTC AE毒性分级标准进行皮肤毒性分级和相应的处理。如需要，可请皮肤科专家会诊并给予治疗建议。

常规皮肤护理：出现皮疹后，忌食辛辣、刺激性食物，保证睡眠。穿着宽松、舒适、非化学合成材料的衣裤、鞋袜，避免皮肤损伤。保持皮肤清洁，避免接触碱性洗涤用品。在皮肤干燥部位涂抹不含酒精的润肤霜。尽量减少日晒时间，皮肤暴露部位涂SPF>15 的防晒用品，在日晒前1-2h使用，可根据日晒持续时间重复使用。

推荐处理：在允许合并用药范围内，可依临床常规进行处理。如需要，可请皮肤科专家会诊并给予治疗建议。上述处理过程完整记录于病历内，合并用药完整记录于病历及CRF相应页中。

皮肤毒性反应处理推荐，详见**附件三。**

5.8.4 腹泻、脱水和电解质紊乱处理

受试者接受SCT200治疗过程中，如果出现腹泻、脱水和/或电解质紊乱情况的，推荐予以积极对症处理。在允许合并用药范围内，可依临床常规进行处理。处理过程完整记录于病历内，合并用药完整记录于病历及CRF相应页中。

5.8.5 低镁血症处理

对于研究过程中发生的其他不良反应处理，合并用药应在允许范围内使用，可依临床常规进行处理。

参考既往抗EGFR单抗类药物如西妥昔单抗级帕尼单抗引起的低镁血症相应的临床处理原则。此类药物引起的低镁血症多为1-2级（血清镁浓度在0.4mmol/L~正常值下限），少数（6%-17%）为3级（血清镁浓度为0.3 mmol/L ~0.4 mmol/L）或4级（血清镁浓度<0.3 mmol/L）。

推荐处理：

研究过程中对于受试者发生1级低镁血症时通常无典型临床症状，建议研究者密切观察受试者临床症状的变化必要时给予处理。对于2级低镁血症，可考虑每周给予静脉补镁（如硫酸镁注射液，4g/次）。此类患者既往同类靶点抗体研究中口服补镁效果欠佳，研究中不作为推荐使用。如发生3级及以上低镁血症，可参考5.5暂停SCT200治疗标准章节标准处理，同时可根据其临床症状严重程度接受给予静

脉补镁治疗（4~10g/次），给药频率为 2 次/周至 1 次/日。

5.8.6 其他可能出现的不良事件

对于研究过程中发生的其他不良反应处理，合并用药应在允许范围内使用，可依临床常规进行处理。处理过程完整记录于病历内，合并用药完整记录于病历及 CRF 相应页中。

5.8.6.1 甲沟炎

有趾甲倒刺(逆剥)者,在给药过程中可能出现甲沟炎及局部增生反应,因此其在接受 SCT200 治疗期间需改变足部受力习惯，穿宽松、透气性好的鞋袜。

对指甲脱色和褶皱等改变,可不做特殊处理。一旦出现甲沟炎,推荐应用金银花水泡足或手，研究者认为必要时可给予莫匹罗星(百多邦)、环丙沙星(达维邦)或夫西地酸（立思汀）外涂。若症状无缓解，可给予米诺环素或头孢呋辛口服，严重者可外科拔甲治疗。

5.8.6.2 间质性肺疾病（ILD）样不良事件

在使用同类抗 EGFR 单抗治疗的患者中，有发生的间质性肺疾病（ILD）样不良事件（包括死亡），但不常见。

若患者出现了急性、新的或进展性、无法解释的肺组织症状，如呼吸困难、咳嗽和发烧等等，应中断 SCT200 治疗，以待进行诊断评价。若已确诊 ILD，患者应停用 SCT200，必要时根据临床常规接受相应的治疗。

5.8.6.3 眼科疾病

在同类 EGFR 单抗治疗期间观察到干燥性角结膜炎、眼睛充血、流泪和眼睛/眼睑刺激等症状。若患者出现了急性眼科疾病或其恶化（如眼痛等），应中断或停止 SCT200 治疗。

5.9 研究药物管理

研究药物发放和回收由专人负责，研究者必须保证所有研究药物仅用于参加该临床研究的受试者，其剂量与用法应遵照研究方案，使用剩余的药物经申办方书面授权同意，可由研究机构进行销毁，未使用的药物应退回申办方，不得将研究药物

转交任何非临床研究参加者。

监查员负责对研究药物的供给、使用、储藏及剩余药物的处理过程进行监查。

5.9.1 运输/接收/储存/保管

经过合适包装的研究用药物由申办方发送到临床研究中心，运输中要保证药物的储存条件和运输注意事项，并有运输条件记录。

申办方与研究中心的药品交接应有接收记录，双方签字及日期。临床研究中心应指定专门的药品管理员统一负责管理药品，例如接收药品及按药物的储存条件储存于试验药物专用冰箱中，统一保管。

储存药物的冰箱需要保存温度记录，时间范围涵盖药物首次接受至最后一次回收，并有专人负责并签字。记录频率合理。

5.9.2 发放/使用/回收/销毁

全部研究用药只有药品管理员才能发放，并由药品管理员记录发放药品的数量。领药人领取药品要有药品领取记录，包括领取药品数量、领取人姓名及日期。药品的配制要有当日药物配制记录。

一旦研究全部完成，研究者必须将所有未使用完的研究药物返还给申办方，由申办方派驻的临床监查员将药物和相应的使用记录一起回收，并记录返回药物的数量和使用情况，签字并署明日期，由申办方统一负责销毁。

5.9.3 记录

在整个研究期间，应对研究药物的运输、接收、发放、领取、使用、返还和销毁情况做详细记录。研究结束时，须保证研究药物的运送记录与使用情况和销毁/返还记录一致，如有差异应予以说明。

5.10 受试者分配方法

所有进入本研究的受试者将接受 SCT200 治疗。在受试者签署知情同意书后，且在进行任何研究相关检查之前，将为每位受试者分配一个唯一的筛选号，筛选号组成形式为：中心号（XX）+顺序号（XXX），如 02 中心第 3 位筛选受试者为 02003，用于整个研究期间受试者的鉴别。受试者筛选号不得重复使用。如某受试者因特殊原因筛选失败后研究者认为可重新参加研究筛选，将给予新的筛选号，并重新签署

知情同意书。

在签署知情同意书、完成所有筛选评估、确定受试者满足所有入排标准后，将给予受试者编号。

5.11 设盲与揭盲规定

不适用。

5.12 合并用药

5.12.1 合并用药/治疗

所有从签署知情同意书到安全性随访访视期间给予的药物均将记录在 CRF 中。也将记录药物增减或剂量变更情况。

研究者可根据本研究人群的临床用药常规进行决定，给予受试者镇静剂、止吐剂、抗生素、镇痛剂、抗组胺药物、类固醇、粒细胞集落刺激因子以及红细胞、红细胞生成素、血小板或新鲜冷冻的血浆输液制品，以帮助治疗疼痛、感染和其他恶性肿瘤并发症。对研究过程中发生低镁血症，可予临床常规治疗。如发生发热性中性粒细胞减少或有证据提示感染，可给予临床常规抗感染治疗。

对于出现的皮肤毒性相关 AE，应积极治疗，必要时请皮肤科医生会诊。允许给予局部和/或口服抗生素治疗（参见第 5.8.3 节及**附件三**）。

如果放射区域非靶病灶位置，允许为缓解疼痛进行局部放射治疗。根据 RECIST 版本 1.1 评估的靶和非靶病灶，如果在试验期间接受过放射，则不得进一步用于缓解评估。如果在试验治疗期间考虑进行任何局部放疗，例如因骨痛加重，应首先根据 RECIST 版本 1.1 确认该受试者是否发生 PD。

发生骨转移时或可给予双磷酸盐。如果因骨痛加重考虑增加正在进行的双磷酸盐治疗剂量或开始双磷酸盐治疗，首先根据 RECIST 1.1 版本确认该受试者是否发生 PD。

5.12.2 禁止用药/治疗

不得给予受试者额外的长期系统性免疫治疗、化疗、放疗、用于治疗癌症的激素治疗或任何其他研究药物。

不允许给予任何被批准用于抗癌治疗的中药（中药说明书中有抗癌或抗肿瘤字

样的不允许使用）。如需要，研究者可决定给予非抗癌治疗适应症的中药，例如支持性治疗的中药。

6. 研究流程及评估指标

6.1 研究流程

6.1.1 受试者入组原则

参加本研究的各研究中心受试者入组为竞争入组。参与本研究的每名受试者均将分配一唯一的筛选号码。一旦确定受试者符合入组和排除标准后，研究中心须将受试者入组审核表格发送给申办方或指定代表，以进一步确认受试者入选资格。受试者将被分配唯一的受试者编号并进入研究。入组状态和受试者编号确认单将被发送回研究中心。

6.1.2 筛选期

访视 1，研究第-28 天~ -8 天

将筛选所有潜在的受试者，在研究入组前确定其入选资格。将在开始研究用药的第-28 天至-8 天内进行下列流程和评估：

1) 获得书面知情同意；
2) RAS 和 BRAF 评估（在本研究指定的中心实验室完成检测）；
3) 收集人口统计学资料；
4) 体格检查（请对皮肤伴随疾病详细检查，并记录）；
5) 记录受试者基线肿瘤相关临床症状；
6) 生命体征及体重测量；
7) 审核既往病史/手术史/治疗史；
8) 12-导联心电图（12-ECG）检查；
9) 评估并记录 ECOG 体能状态（参考附件一）；
10) 实验室检查：血常规、尿常规、血生化、凝血功能、病毒学检查（包括 HBV、HCV 及 HIV）；
11) 妊娠检测：对于非绝经后或未接受过手术绝育术的女性，需进行血清 β-人绒毛膜促性腺激素（β-hCG）妊娠检查；

12) 记录组织病理学肿瘤诊断结果，如需要，可将病理学样本递交研究中心病理审核。

- 结直肠癌初始诊断及复发时采集的肿瘤活检标本；

13) 根据以下影像检查流程测量并记录可测量病灶参数：

- 胸、腹和盆腔的增强 CT。详细技术要求请参考本研究提供的中心影像研究中心成像手册；
- 记录胸、腹和盆腔之外其他疾病部位的其他相关影像（如适用）；

14) 采集血清肌酐血样，根据下列 Cockroft and Gault 方程用肾小球滤过率（GFR）计算肌酐清除率：

$$GFR^* = \frac{(140-\text{年龄[岁]}) \times \text{实际体重（kg）}}{72 \times \text{血清肌酐}}$$

*对于女性受试者，将根据上述公式得出的结果乘以 0.85 作为其结果；

15) 审核入选本研究的资格标准；

16) 获得 ICF 后收集不良事件信息；

17) 收集合并用药信息。

访视 2，研究第-7 天~ -1 天

1) 体格检查；
2) 生命体征；
3) 记录体重；
4) 12-ECG 检查；
5) 评估并记录 ECOG 体能状态（参考附件一）；
6) 实验室检查：血常规、尿常规、血生化和凝血检查，上述检查需在首次研究用药前 3 天内完成。如访视 1 某些实验室检查日期满足此要求，相应检测在基线期可不复测；
7) 体格检查、生命体征、12 导联心电图、ECOG 体能状态评分可接受 7 天（1 周内）结果，如访视 1 体格检查、生命体征、12 导联心电图、ECOG 体能状态，相应检查在基线期可不复测；
8) 妊娠检测：对于非绝经后或未接受过手术绝育术的女性，需进行尿 β-人绒

毛膜促性腺激素（β-hCG）妊娠检查；

9) 获得筛选及基线期所有检查结果后，应依据**受试者入组审核表格**内容，确认受试者入选研究资格；

10) 收集不良事件信息；

11) 收集合并用药信息。

6.1.3 研究治疗期

访视 3 开始至研究提前退出访视/随访期前，包括每周 1 次研究用药/每 2 周 1 次研究用药、每 4 周 1 次安全性评估、每 8 周次疗效评估。

1) 体格检查（访视 3 无需进行）；
2) 生命体征检查；
3) 记录体重（访视 3 无需进行）；
4) 12-ECG 检查；
5) 评估并记录 ECOG 体能状态（访视 3 无需进行，参考附件一）；
6) 收集不良事件信息；
7) 收集合并用药信息；
8) 实验室检查：血常规、血生化、尿常规和凝血功能检查（访视 3 无需进行）
9) 将进行下列流程/检查以评估疗效（访视 3 和安全性评估时无需进行）：

A. 医学影像检查（采用与筛选时相同的成像技术，无论在研究中心或中心评估，建议在整个研究期间尽可能由同一名影像科研究人员完成所有评估）

i. 胸、腹和盆腔的增强 CT 扫描。对于在基线时具有可能的骨转移或在试验期间被怀疑发生骨转移的受试者应考虑进行骨扫描和/或正电子发射计算机断层扫描术（PET）扫描以支持 CR 及 PD 诊断。详细技术要求请参考本研究提供的中心影像研究中心成像手册；

ii. 记录胸、腹或盆腔之外、疾病相关其他部位影像检查结果（如适用）；

B. 体格检查；

C. 如进行肿瘤活检，应对活检标本完成病理学评估，以确认最终疗效评估结果；

本研究将按照 IRC 章程所述，所有的影像图像将被送至独立的中心影像实验室

进行独立的中心影像阅片。具体细节将在中心影像研究中心成像手册及独立影像阅片章程中描述。IRC 阅片将依据 RECIST 1.1 版（见附件四）对研究抗肿瘤疗效进行评估。

PD 需基于影像学评估结果进行确认。如果发生了提示 PD 的临床症状，需安排计划外访视以完成影像学评估确认。

此外，研究者也将根据 RECIST 1.1 版标准及中心影像研究中心成像手册进行疗效评估，与 IRC 评估相对独立。

6.1.4 研究提前退出访视

如果某名受试者撤销知情同意并拒绝继续参加随访期访视，应在受试者撤销知情同意前完成以下流程。

1) 如果发生下列情况，参考第 6.2 章节规定进行疗效评估：
   - 受试者至少接受过一次 SCT200 治疗；
   - 受试者除接受 SCT200 治疗外，尚未开始后续针对 mCRC 的抗肿瘤治疗，且距上次肿瘤评估≥4 周；
2) 体格检查；
3) 生命体征检查；
4) 测量体重；
5) 12-ECG 检查；
6) 评估并记录 ECOG 体能状态（参考附件一）；
7) 实验室检查：血常规、血生化、尿常规和凝血功能检查；
8) 妊娠检测：对于非绝经后或未接受过手术绝育术的女性，需进行尿 β-人绒毛膜促性腺激素（β-hCG）妊娠检查；
9) 收集不良事件信息；
10) 收集合并用药信息。

6.1.5 随访期

安全性随访访视

所有至少接受过一次 SCT200 治疗的受试者将在最后 1 次研究用药后 28（+7）

天进行安全性随访访视，包括下列流程和评估：

1) 体格检查；
2) 测量体重；
3) 生命体征检查；
4) 12-ECG 检查；
5) 评估并记录 ECOG 体能状态（参考附件一）；
6) 实验室检查：血常规、血生化、尿常规和凝血功能检查；
7) 妊娠检测：对于非绝经后或未接受过手术绝育术的女性，需进行尿 β-人绒毛膜促性腺激素（β-hCG）妊娠检查；
8) 收集不良事件信息；
9) 收集合并用药信息；
10) 如果受试者未发生 PD 且尚未开始针对 mCRC 的后续抗肿瘤治疗，则完成疗效评估流程（参见第 6.2 章节规定）；
11) 记录死亡日期（如适用）；
12) 记录后续的针对 mCRC 的抗肿瘤治疗（如适用）。

疾病进展随访访视

在安全性随访后，对于尚未发生 PD 且未开始后续抗肿瘤治疗的受试者，将继续每 8 周进行 1 次疗效评估，具体操作流程见 6.2 章节相关规定，直至受试者发生 PD、开始后续抗肿瘤治疗、死亡或失访。

生存情况随访访视

在安全性随访访视和/或疾病进展随访访视后，将根据标准的临床治疗进行生存情况随访。所有至少接受过一次 SCT200 治疗的受试者均需进行生存情况随访，这些访视计划在安全性随访/疾病进展随访访视后每 3 个月（±14 天）1 次，可通过电话问询方式进行，以收集安全性访视后开始的后续抗肿瘤治疗以及生存状况信息，直至至少 85%受试者出现疾病进展、开始后续抗肿瘤治疗、死亡、提前退出研究或失访。应在每次生存情况随访访视时进行下列流程/评估：

1) 收集研究药物相关不良事件信息；
2) 进行疗效评估（见第 6.2 章节规定）；

3) 记录所有后续针对 mCRC 的治疗（如适用）;

4) 记录死亡日期（如适用）。

6.2 有效性评估指标

6.2.1 主要疗效指标

客观缓解率（ORR）：研究治疗后的ORR，定义为获得完全缓解（CR）或部分缓解（PR）最佳总疗效（BOR）的受试者比例。

6.2.2 次要疗效指标

1) 左半结直肠癌亚组ORR：入选受试者中左半结直肠癌亚组的ORR；
2) 疾病控制率（DCR）：定义为获得完全缓解（CR）、部分缓解（PR）和疾病稳定（SD）的受试者比例；
3) 缓解持续时间（DOR）：是指第1次疗效评估为CR或PR开始至第1次疗效评估为PD或任何原因死亡的时间；
4) 治疗缓解时间（TTR）：是指从研究用药第1天开始至出现缓解的时间；
5) 无进展生存期（PFS）：是指从研究用药第1天至第1次PD或死亡时间；
6) 总生存期（OS）：是指从研究用药第1天至死亡时间。

6.3 安全性评估指标

6.3.1 安全性评估指标

接受至少 1 次研究用药的所有受试者均需接受安全性评估，研究者依据 CTCAE V4.03 标准评估不良事件。

将在整个研究期间密切监测受试者不良事件的发生情况。安全性结果包括不良事件（AEs）、严重不良事件（SAEs）、因任何原因的停药、因不良事件停药、实验室检查异常、生命体征改变及体格检查结果改变。受试者将随访至经研究者判定 AE 或其他异常情况已缓解、恢复至基线水平或无临床意义、或被确定为不可逆。研究者认为有临床意义的实验室检查结果应当记录为 AE。此外，研究者应当确定 AE 与研究药物给药之间的关系。

本研究的实验室检查内容详见**表 3**。将参考各研究中心的正常值范围用于本研究各项实验室检查参数的判断。

表 3 实验室检查内容

项目	观察指标
心电图	标准 12-ECG
血常规	红细胞计数、血红蛋白浓度、白细胞计数、血小板计数、中性粒细胞绝对值、淋巴细胞绝对值、单核细胞绝对值
尿常规	尿蛋白（PRO）、尿糖（GLU）、尿白细胞、尿红细胞
凝血功能	凝血酶原时间（PT）、活化部分凝血活酶时间（APTT）、D-二聚体、FIB
血生化	谷草转氨酶（AST）、谷丙转氨酶（ALT）、碱性磷酸酶（ALP）、谷氨酰转肽酶（GGT）、尿素（UREA）、血肌酐（CREA）、总蛋白（TP）、白蛋白（ALB）、总胆汁酸（TBA）、A/G比、总胆红素（TBIL）、直接胆红素（DBIL）、肌酸激酶（CK）、胆碱酯酶（PCHE）、空腹血糖（Glu）、总胆固醇（TCHO）、低密度脂蛋白胆固醇（LDL-C）、高密度脂蛋白胆固醇（HDL-C）、甘油三酯（TG）Ca^{2+}、Na^{+}、Cl^{-}、K^{+}、Mg^{2+}

6.3.2 免疫原性评估指标

采用 SCT200 I 期临床研究中已建立的免疫原性检测方法（MSD）检测本研究受试者用药前后血清中抗 SCT20 抗体水平以评估免疫原性。

血样采集时间计划：

- 血样采集时间：受试者应分别于基线、开始研究治疗后肿瘤疗效评估时间点（第 7 周，及之后每 8 周±7 天）进行血样采集，采集时间为给药前；
- 提前退出研究受试者需在研究提前退出访视时采集该血样；
- 进入研究随访期的受试者需在安全性随访访视中采集该血样。

6.4 探索性研究评估指标

结直肠癌的发生发展是多基因、多分子、多通路间错综复杂、相互作用的结果。通过对组织和血液样本结直肠癌关键基因(ctDNA)的检测，可比对组织和血液样本之间基因变异谱图变化的相关性；同时，可动态监测用药前、用药过程中及进展后基因变异信息，结合临床证据，更好预测 SCT200 疗效和耐药机理，以及进一步的个体化治疗方案选择指导。

探索性研究重点对**KRAS、NRAS、BRAF、 PIK3CA、EGFR等**多个影响结直肠癌个体化治疗的热点基因变异信息进行筛查和检测。如有可能，也将基于同类药

物相关的新科学发现数据对可能影响SCT200 药物疗效、受试者疾病和/或安全性相关的其它潜在生物标志物进行查找。

探索性研究样本采集：

- **肿瘤标本**：可来自筛选期RAS/BRAF基因野生型确认分析所用相同或剩余的样本。具体要求按中心病理实验室手册操作指南；
- **ctDNA 血液样采集时间点**：应于筛选期、开始研究治疗后肿瘤疗效评估时间点（第 7 周，及之后每 8 周±7 天）、以及肿瘤进展时获取血液样本。样本采集具体要求按中心病理实验室手册操作指南。

7. 安全性评估

7.1 不良事件和严重不良事件处理

7.1.1 定义

不良事件（AE）：指临床试验受试者接受试验用药物后出现的所有不良医学事件，可以表现为症状体征、疾病或实验室检查异常，但不一定能推论出与试验药物有明确的因果关系。

AE 可以是：

- 任何不利和非预期的体征（包括由于研究药物用药差错、过量使用或与其他药品相互作用引起的）；
- 任何新的疾病或一种既有疾病的加重（例如发生频度增加或性质加重）；
- 研究者判断为有临床意义的实验室检查/其他检查异常（新出现或基线异常加重）基线时未出现的一种间歇性医学状况（例如，头痛）的反复发生；
- 其他医学事件，不论与研究药物关系如何，例如意外、跌倒或任何因这些事件产生的受伤。

严重不良事件（SAE）是满足下列任意条件的 AE：

- 导致死亡；
- 危及生命（即，在发生时受试者具有因这种 AE 即刻致死的风险）；
- 受试者需要住院治疗或延长既有的住院时间；

注：由于择期手术（参与研究前已经计划好）、非医疗原因（福利，疗养或

某种便利）导致的住院不被认为是 SAE。

- 造成永久性或显著性的功能丧失或残废；
- 导致先天畸形/出生缺陷；
- 重要医学事件；

重要医学事件的评估：

某些医学事件虽然不符合上述 5 项 SAE 标准，不会立即危及生命、导致死亡或住院，但可能损害受试者或需要治疗干预以防出现以上情况，这些被研究者判断为可能导致严重临床后果的情况即重要医学事件。重要医学事件的判定是基于研究者的医学知识及临床经验。重要医学事件也需按照 SAE 报告程序报告。

治疗期间不良事件（TEAE）：临床试验受试者接受研究药物后新出现的，或者在给药前已经存在、给药后加重的任何不良医学事件。

7.1.2 不良事件严重程度

不良事件的严重程度是对研究者确定的或受试者报告的不良事件的范围或程度的定性评价。严重程度不反映事件在临床上的严重性，也不反映与研究药物的相关性。

所有不良事件的严重程度需按照美国国家癌症研究所（NCI）发布的常见不良反应/事件评价标准（CTCAE，4.03 版，2010/06/14）进行分级。对于 NCI-CTCAE 中未列出的不良事件，可参照**表 4**进行分级：

表 4 不良反应/事件评价分级标准

1 级	轻度；无症状或轻度症状；仅临床或诊断发现；无需治疗；
2 级	中度；最小的、局部的或非侵入性治疗指征；年龄相关工具性日常生活活动受限*；
3 级	重度或重要医学意义，但不会立即危及生命；住院治疗或延长住院时间指征；致残；自理性日常生活活动受限**；
4 级	危及生命，需紧急治疗；
5 级	死亡

*工具性日常生活活动是指做饭、购买杂货或衣服、使用电话、理财等等。

**自理性日常生活活动是指洗澡、穿衣和脱衣、进食、如厕、服用药物，而不是卧床不起。

7.1.3 不良事件/严重不良事件与研究药物相关性判定

不良事件与研究药物相关性的判定标准：

研究者需对研究中发生的所有 AE/SAE 进行与研究药物相关性的判定。研究者应基于常规临床诊疗规则， 且参考研究者手册或产品信息完成相关性判断。研究者在判断 AE/SAE 与研究药物的相关性，需同时考虑以下因素：与研究药物用药时间是否有合理的时间顺序；所出现的症状/体征是否可以用研究药物的作用机制解释；减量或者停药后，症状/体征是否好转；再次用药后，症状/体征是否再次出现或加重；是否能用其他原因解释， 例如基础疾病、伴随疗法、其他危险因素等。根据药物与 AE/SAE 因果关系判断标准，将 AE/SAE 与研究药物的相关性分为 5 级：肯定有关、可能有关、可能无关、肯定无关、无法判定 （备注：仅由客观原因无法获得进一步信息，不足以判断相关性时，才可以选择这一项）。其中“肯定有关、可能有关、无法判定”将被判定为与研究药物有关；“可能无关及肯定无关”将被认为与研究药物无关。

7.1.4 不良事件/严重不良事件的记录与报告

从签署知情同意书开始至末次研究用药后 28 天内发生的所有的 AE（除外依据方案规定，不需要搜集的事件），均需要记录在 eCRF“不良事件页”中。需要记录以下内容：AE 名称、发生日期、CTCAE 分级、对研究药物采取措施、AE 转归、结束日期、与研究药物关系和是否是 SAE 等。

疾病进展：本研究目标恶性肿瘤的自然进展或恶化将纳入有效性评估部分进行记录，不记录/报告为 AE/SAE。

试验中发生的任何SAE（除外依据方案规定， 不需要搜集的事件），除在eCRF中记录外，研究者需填写CFDA版SAE报告表，在获知后的 24 小时内，报告给国家/省食品药品监督管理局（CFDA/PFDA）、国家卫生和计划生育委员会（NHFPC）、伦理委员会（EC）、组长单位负责人和申办方，详见**表 5**。 SAE报告应至少包含以下四个要素：1）可识别的报告人；2）可识别的受试者（例如受试者编码）；3）研究药物；4）SAE。注：以上四要素仅仅是构成一个有效SAE报告的四个必要条件。

除以上四要素外，应按照时间顺序，详细记录SAE的事件经过 （SAE报告填写可参考“SAE填写指导”）。所有的AE/SAE均需要随访至事件解决、状态稳定或恢复到基线状态。

在疾病进展随访和生存随访期间，仅当事件与研究药物因果关系被判定为“可能有关”“肯定有关”时，才需记录在 eCRF “不良事件”页。若此 AE 被研究者评估为 SAE，则需按照 SAE 程序报告。这些 AE/SAE 需随访至事件解决、状态稳定或恢复至基线状态。

表 5 严重不良事件报告单位

报告单位	邮箱	联系电话	传真
神州细胞工程有限公司	SCT-safety@sinocelltech.com	010-58628288-9492	010-58628299
各中心伦理委员会	-	-	-
国家食品药品监督管理总局注册司	-	010-68313344	010-88363228
国家卫生和计划生育委员会医疗安全与血液管理处	-	-	010-68792734

7.2 实验室检查异常值处理

符合以下任何一种情况的实验室检查异常值通常被认为具有临床意义：

- 这些异常导致研究药物给药日程变化（剂量变化、延迟给药、暂停或永久停用研究药物）；
- 这些异常需要其他医学或治疗干预（例如，需要输血的贫血或需要补充钾的高血糖）；
- 这些异常提示新的疾病或原有疾病恶化。

研究者需根据受试者自身实际情形，结合以上情况， 经医学判断，给出实验室检查异常值“有临床意义”或“无临床意义”的结论。

有临床意义的实验室检查异常值满足 AE 或者 SAE 定义时，需记录在 eCRF “不

良事件”页。筛选期间具有意义的实验室检查异常值，如果是受试者原发恶性肿瘤或伴随疾病的表现，则此异常值不被认为是 AE 或者 SAE（例如：筛选期实验室检查结果提示受试者血糖升高，受试者伴随疾病有糖尿病，则血糖升高不被认为是 AE/SAE）。在筛选期间出现的其他的无法用原发恶性肿瘤或者伴随疾病解释的、被判定为具有临床意义的实验室检查异常值，需要记录为 AE/SAE。如果该有临床意义实验室异常值是某一疾病诊断的表现，则只需要记录疾病诊断为 AE 或者 SAE（例如：实验室检查提示受试者红细胞降低，后受试者诊断为贫血， 则只需要记录贫血为 AE 或者 SAE）。

上述实验室检查异常值的处理原则，同时适用于生命体征及体格检查异常情况的处理。

7.3 妊娠事件处理

首次使用研究药物开始至末次使用研究药物后 120 天之内，如果女性受试者或男性受试者配偶发生妊娠，研究者需在获知后 24 小时内，填写妊娠报告表，向申办方报告。所有的妊娠事件需随访至妊娠结束/终止。自然流产，选择性流产，引产，异位妊娠，任何胎儿异常（例如：胎停育，葡萄胎，死胎，死产，胎儿畸形）等均被认为是 SAE，需按照 SAE 的程序报告。这些事件均需随访跟踪至事件结束或稳定。女性受试者一旦确定妊娠，将被终止参与研究。男性受试者配偶发生妊娠，将需按照上述要求跟踪随访。

7.4 生命体征及体格检查

生命体征或体格检查评估的 AE 包括任何下列情况的变化、检测值或结果（异常）：

- 导致医学干预；
- 和/或被研究者认为具有临床意义；
- 和/或符合临床关注的异常标准。

7.5 12-ECG 不良事件

受试者需按照试验流程接受 12-导联静息 ECG 检查应尽可能由同一名医师阅读给定受试者的所有的 ECG，以保持判断的一致性。研究者判断为具有临床意义的异

常检查结果应记录为 AE 或 SAE。

7.6 其他安全性考虑及风险管理

研究者应根据方案设计给予受试者研究药物，用药差错应立即作为方案背离向申办方/CRO 报告。用药差错可以导致 AE/SAE 发生或无临床异常结果。

例如：

- 药物过量 -对于 SCT200 用药过量的治疗尚无特定信息。如果研究者怀疑受试者接受过量研究药物且引发 AE/SAE，研究者需采取必要的临床处理措施以保证受试者安全，记录方案违背的同时，应记录或报告此 AE/SAE。如果研究者怀疑受试者接受过量药物，经密切观察，受试者无临床异常症状发生，则仅需按照方案违背记录。
- 药物转移 - 定义为研究治疗有意或意外的销售或给予其他人。这可以包括研究药物意外错发到医院常规主供应药品系统。药物转移后在非受试者个体发生的不良事件均需按照 7.1.4 中原则处理。

任何被发现造成潜在风险（例如，因与其他产品相似或说明不清晰）的包装或标识均须立即报告申办方。

8. 统计分析

8.1 样本量

本研究为单臂、多中心II期临床试验，其主要分析将基于对结直肠癌总体人群ORR的评估。试验计划入组并治疗110例结直肠癌患者，考虑10%的脱落率，可提供99例的有效性分析样本量。对主要疗效指标ORR进行如下优效性假设检验（exact test）：

H_0: ORR ≤ 20% vs. H_1: ORR > 20%,

在单侧 α=0.025 的水平上，99 例的样本量可提供>93%的把握度检测到 15%的优效差异。

此外，该样本量可为ORR的估计提供的精度为 20.4%以上（即 95%置信区间宽度≤ 20.4%）。**表 6**中列出了对应不同的ORR观察值，99 例的样本量所提供的 95%置信区间。当ORR观察值为 35% 时，95%置信区间为（25.7%，45.2%），其下限大于

20%，显示试验药对于结直肠癌的疗效具有显著临床意义；当ORR观察值为 20%时，95%置信区间为（12.6%，29.4%），其 上限小于 35%，显示试验药对于结直肠癌的疗效不具有显著临床意义（参见 8.3.4.1 章节）。

表 6　总数 99 例结直肠癌患者对应 ORR 观察值所提供的 95%置信区间

ORR 观察值	95%CI 下限	95%CI 上限	95%CI 宽度
20%	12.6%	29.2%	16.6%
25%	16.8%	34.7%	17.9%
35%	25.7%	45.2%	19.6%
40%	30.3%	50.3%	20.1%
45%	35.0%	55.3%	20.3%
50%	39.8%	60.2%	20.4%
55%	44.7%	65.0%	20.3%
65%	54.8%	74.3%	19.6%

8.2 分析人群

全分析集（full analysis set, FAS）：包括所有接受至少一次研究用药的受试者。

安全性集（safety set，SS）：包括所有接受至少一次研究用药，且具有至少一次用药后安全性评估的受试者。

符合方案集（per-protocol set, PPS）：包括 FAS 中所有研究期间未使用影响有效性评价的合并用药、主要评价指标数据完整且无重大试验方案违背的受试者。导致患者被排除在符合方案集之外的所有试验方案偏离将在统计分析计划（SAP）中详细描述，并在数据锁定前完成。

8.3 统计分析方法

计划统计分析的数据截止时间为所有受试者至少完成第一次确认的疗效评估后，或至出现以下情况：疾病进展、开始后续抗肿瘤治疗、死亡、提前退出研究或失访（即统计分析数据截止时间点不早于上述时间点）。

统计分析将采用 SAS 9.4 进行编程计算。

计划将参加本次研究的各个中心的数据合并起来进行分析。使用描述性统计值对人口统计学和基线特征、有效性和安全性等数据进行分析。对于连续型变量，列出例数、均数、标准差、中位数、最小值、最大值。对于分类变量，列表描述其频数和百分数。

基线定义为首次用药前的最后一次观测数据。

详细的分析方法将在统计分析计划中给出。

8.3.1 病例分布

在全分析集中，对各中心入组的受试者例数和百分比、试验完成情况、及退出原因进行总结。

8.3.2 基线及人口学特征

在全分析集中，对人口学和其他筛选数据（包括疾病特征）进行列表和描述性汇总。

对相关的病史及当前医疗状况按系统器官分类及首选术语分类(MedDRA)进行汇总。

8.3.3 依从性及药物暴露分析

通过有方案偏离的患者人数和比例评估对方案的依从性；根据减量和暂停治疗次数对研究药物的依从性进行评估。分析将以列表和汇总的形式进行。

列出实际剂量和治疗天数以及剂量强度（计算方法为，所达到的实际剂量/实际持续时间）和相对剂量强度（计算方法为，剂量强度与计划剂量/计划持续时间的比值），并用描述性统计量进行汇总。相对剂量强度的类别规定为，< 0.5、≥ 0.5 - < 0.8、≥ 0.8 - < 1.0、≥ 1.0 - < 1.2 和≥ 1.2。给出每个类别内患者的人数和比例。

汇总终止治疗的原因，并列出首次和末次给药的日期、暴露持续时间和每名患者的停药日期。

8.3.4 有效性分析

在全分析集上对疗效指标进行分析。

8.3.4.1 主要疗效指标

主要疗效指标为结直肠癌受试者总体人群中的客观缓解率（ORR）。ORR 定义为依据 RECIST 1.1，肿瘤中心影像评估最佳疗效为确认的 PR 和 CR 的受试者的比例。帕尼单抗与西妥昔单抗在相近研究人群中 ORR 分别为 22.0%和 19.8%（参考 ASPECCT 研究），在此基础上>15%的提高被认为具有显著临床意义。因此，将总体结直肠癌 ORR 划分为如下疗效范围：

- ORR <20% ，无临床意义；
- 20% ≤ ORR <35%，有限临床意义;
- ORR ≥ 35%，显著临床意义。

作为主要分析方法，将在单侧$\alpha = 0.025$的水平上，对主要疗效指标ORR进行如下优效性假设检验（exact test）：

H_0: ORR ≤ 20% vs. H_1: ORR > 20%。

将给出 ORR 观察值并提供 95%确切（exact）置信区间。如果 ORR 观察值大于 35%，且 95%置信区间下限大于 20%，则认为试验药在治疗结直肠癌患者总体人群中具有显著的临床意义。

缺失值处理

在主要分析中，BOR 为未知（依据 RECIST 1.1 定义）或无（即无治疗后肿瘤评估有效记录）的受试者将被认为无缓解。

支持性分析

将采用Bayesian方法对ORR进行分析。根据同类药物西妥昔单抗和帕尼单抗ASPECCT研究的结果，设定ORR的先验均值为 20%，采用相应的最小信息先验概率分布Beta(0.25,1)[11]，获得ORR后验概率分布，估计ORR并提供不确定性描述，给出ORR在上述各疗效范围的后验概率。

此外，如果符合方案集与全分析集所包括的病例不同，在符合方案集上重复上

述所有分析。

8.3.4.2 次要疗效指标

次要疗效指标包括左半结直肠癌受试者人群 ORR、最佳疗效（BOR），疾病控制率（DCR），缓解持续时间（DOR）,至 缓解时间（TTR），无进展生存期（PFS）及 6 个月、9 个月和 12 个月 PFS 率，总生存期（OS）。其中肿瘤响应依据 RECIST 版本 1.1 进行评估。

针对总体人群及左、右半结直肠癌亚组人群分别进行以下分析：

- 提供左、右半结直肠癌亚组人群的 ORR 观察值并提供 95%确切（exact）置信区间。如果符合方案集与全分析集所包括病例不同，在符合方案集上重复上述分析。
- 对 BOR 进行汇总，对每个疗效类别的受试者比例给出观察值及 95%确切置信区间；对受试者肿瘤体积较基线最佳变化进行图示。
- 提供 DCR 的观察值及 95%确切置信区间。
- 采用 Kaplan-Meier 方法对 PFS 和 OS 进行分析，提供中位数及 Brookmeyer-Crowley 95%置信区间，并提供 6 个月、9 个月和 12 个月的 PFS 率。
- 对 DOR 和 TTR 做列表和汇总。

8.3.5 安全性分析

安全性评价终点的分析在安全性分析集中进行。

- **不良事件**

不良事件采用国际医学用语辞典（MedDRA）中文版进行编码，按照系统器官分类（SOC）和首选术语（PT）对 TEAE 进行汇总。统计所有患者的不良事件及不良反应的发生例数、发生率及发生例次。另外，按 AE 的严重程度、与研究用药的相关性分别计算各类别患者例数和百分比。列出不良事件清单。筛选期及用药前发生的 AE 只在列表中给出。

- **实验室检查**

对各实验室检查，在各计划时间点对各指标进行描述性汇总分析，并以治疗前

后交叉表（根据临床意义判断）的形式总结所有完成的检查项目。列出受试者的检查项目治疗前正常治疗后异常，以及治疗前异常治疗后异常加重的项目的清单，并进行汇总。

- **生命体征**

汇总出现异常生命体征的患者例数及百分比，并以治疗前后交叉表（根据临床意义判断）的形式进行汇总。列出受试者的检查项目治疗前正常治疗后异常，以及治疗前异常治疗后异常加重的项目的清单，并进行汇总。对于至少出现 1 例异常的受试者的生命体征评估列表。

- **ECG**

汇总出现异常 ECG 的患者例数及百分比，并以治疗前后交叉表（根据临床意义判断）的形式进行汇总。列出受试者治疗前正常治疗后异常，以及治疗前异常治疗后异常加重的 ECG 评估清单，并进行汇总。对于至少出现 1 例异常的受试者的心电图评估列表。

- **免疫原性分析**

提供受试者血清中抗 SCT200 抗体水平随时间的变化图，并进行描述性汇总。

8.3.6 合并用药

治疗开始之前和之后的伴随药物和重要非药物治疗按患者列出，按 ATC 术语（解剖-治疗-化学分类系统）汇总。

8.3.7 亚组分析

针对左、右半结直肠癌亚组进行有效性分析（见 8.3.4.2 节）。

9. 研究的开展和监督职责

9.1 申办方

本研究申办方为神州细胞工程有限公司。

9.2 伦理学和监管

本研究的开展将符合申办方和合同研究组织（CRO）的标准操作规范，这些规范旨在确保遵循药物临床试验质量管理规范（GCP）指南。研究者在签署方案时将同意遵循方案中阐述的说明和流程，并遵循本方案所符合的药物临床试验质量管理规范原则以及所有管理中国法规和医学研究的原则。

9.2.1 伦理委员会

在研究开始前，研究者有责任向伦理委员会提供临床研究方案、知情同意书以及提供给受试者的信息资料（招募受试者的广告、受试者补偿方案等）递呈伦理委员会审批，以获得实施该临床研究的独立批准文件。伦理委员会的批准文书须以书面的形式送交研究者，然后再由研究者将批准文件的副本提供给申办方。伦理委员会的批准文件须附有所有参与批准文件讨论的委员会成员的名单及其各自的专业背景。

在临床研究过程中，任何与临床研究安全性相关的问题，如临床研究方案或受试者知情同意书的更改以及临床研究中的严重不良事件，都必须及时向伦理委员会报告。临床研究的结束或提前终止也都必须及时向伦理委员会报告。

9.2.2 受试者知情同意

受试者的权利、安全性和身心健康是最重要的考虑因素，应优先于科学和社会利益。研究者必须在知情同意中向每一位受试者，或者在受试者不能给出同意的情况下向其法定代表，清晰并全面地告知本次研究的性质、目的、有关程序、预期时间、潜在风险和利益、以及可能出现的任何不适。每位受试者必须知道参加该研究是自愿的，他/她在任何时候都可以退出该研究以及撤回知情同意，不会影响他/她随后的治疗和与治疗医生的关系。知情同意书应采用标准的书写格式给出，并尽量使用非专业语言。每份知情同意书必须包括以上所有的有关内容，并包括一项自愿声明。知情同意书需递交有关的伦理委员会审批。

研究者在解释了有关研究的基本内容，并且已确信受试者理解研究的目的后，应要求每位参加研究的受试者在知情同意书上签署姓名及日期。受试者在签署姓名和日期之前应仔细阅读和考虑其声明，并应获得签署文件的复印件。在开始任何与

临床研究相关的操作程序之前，必须获得受试者的知情同意书，包括为了确定受试者参与本研究资格而进行的任何筛选流程的知情同意。未获得知情同意，受试者不能进入研究。

对于那些因任何原因而不能自己签署知情同意书的受试者，须由其法定监护人或保护人签署知情同意书。由受试者亲自签署姓名和日期的知情同意书原件必须由研究者妥善保存，在病例报告表和相关研究原始记录中也需记录。

9.2.3 研究者

研究者职责：

1) 受试者入选前，研究者应向入选者或家属解释研究的意义，征得其同意并签署知情同意书
2) 发生不良事件时，研究人员需调查其原因并做出相应处理，同时，需上报研究负责人。如果受试者死亡，应负责提供受试者的组织病理学及其他相关资料
3) 将数据真实、准确、完整、及时、合法地载入病历和病例报告表(CRF)
4) 配合申办方委派的临床监查员的定期检查
5) 完整保留实验室检查记录、临床记录以及受试着的原始医疗记录
6) 随访严重不良事件

9.2.4 质量保证和检查要求

将遵循方案、申办方和 CRO 的 SOP 要求组织、开展和报告这项研究。在 ICH E6 中，质量保证（QA）定义为“所有那些拟定和系统性的旨在确保试验实施和数据产生、记录和报告遵循药物临床试验质量管理规范（GCP）和适用的监管部门要求的行动”。申办方 QA 活动将根据研究稽查计划的规定进行。ICH E6 第 5.19.3（b）节指出，稽查计划和试验稽查流程的制定应受到该试验在向有资格的主管部门的申报资料中的重要性、该试验中的受试者数、试验类型和复杂程度、试验受试者的风险水平以及任何发现的问题的指导。QA 活动可外包给 CRO 或独立的咨询机构。要求研究者支持稽查活动，按稽查员要求出席，并允许稽查员直接调阅原始数据/文档。

有资格的主管部门（CA）/或获得授权的第三方也可能进行检查（研究期间或在研究完成之后）。如果某 CA 提出检查要求，研究者须立即通知申办方收到此检查

要求。

9.3 数据管理

研究者的主要职责是确保以 CRF 或其他形式报告的数据准确、完整、及时，而且应保证 CRF 上的数据来自于受试者的源数据。

本研究将采用电子数据采集系统（Electronic Data Collection, EDC）进行数据的收集和管理。数据管理过程应符合《药物临床试验质量管理规范》（Good Clinical Practice, GCP）及相应法规要求，遵照数据管理部门的标准操作规程（SOP），确保临床试验数据的真实，准确，完整，可靠和可溯源性（EDC 系统将记录所有稽查轨迹）。数据管理的详细内容将在数据管理计划中提供。

9.3.1 数据录入

本研究数据录入为直接录入电子数据采集系统（Electronic Data Collection, EDC），研究者或 CRC 根据受试者的原始资料信息，准确、及时、完整、规范地通过 EDC 系统填写受试者信息。

临床监查员应监督临床试验是否遵循试验方案，进行原始资料核查（Source Document Verification, SDV），确认所有电子病例报告表（eCRF）填写与原始资料一致。如有错误和差异，应通知研究者，并根据所发现的错误或差异，记录相应的质疑，以确保所有数据的记录和报告正确和完整。

9.3.2 数据核查和质疑管理

根据数据核查计划进行数据核查，主要包括人工核查以及计算机系统的逻辑核查。核查产生的质疑需由研究者或得到授权的 CRC 进行回答，如果研究者的答复解决了质疑，数据管理人员将关闭质疑，如果质疑未被解决则质疑会再次发出。此过程持续直至数据清理完成。

9.3.3 数据库锁定

根据数据库锁定流程，一旦完成所有锁库前步骤，应书面批准数据库锁定，收回数据库的数据编辑权限，进行数据库锁定。数据库锁定之后发现的问题如需修改，应严格按照解锁和再锁定的流程，进行数据的修改。

9.4 临床监查

将由 CRO 有资质的人员监查本研究。监查计划将详细描述该过程。研究者需按照要求，允许对临床、实验室和药房设施进行监查、稽查和视查，以确保遵从药物临床试验质量管理规范和实验室质量管理规范。EDC 系统和受试者对应的原始医学记录（原始文件）需准备好供 CRO 代表定期审核。这些审核验证对研究方案的遵循以及数据按照当地法规和药物临床试验质量管理规范要求的准确性。研究中心的所有记录均会受到当地有资格的主管部门以及申办方 QA 部门的检查。

9.5 医学监查

本研究的具体医学监查工作内容将在单独的医学监查计划（MMP）中规定，包括医学监查概念、主要参与人员及其职责分工、本研究医学监查流程及完成时限等重要内容。MMP 的准备将自研究方案定稿开始，最迟至首例受试者入组前完成，在后续医学监查工作开展中如需要将进一步更新，以保证本研究医学监查工作开展的科学性及合理性。研究医学监查员以及其他联络人的联系方式及信息将在研究中心文件中列出。

9.6 资料的保存

研究者应当使资料保存完整。按照我国 GCP 原则，研究者应保存临床研究资料至研究结束后五年。申办者应保存临床研究资料至研究药物被批准上市后五年。

10. 研究结果注册及发表

临床研究开始前，申办方将在 CFDA 的药物临床研究登记与信息公示平台（http://www.chinadrugtrials.org.cn/eap/main）登记并公示。研究结果的公布遵循国家相关法规要求。

申办方在临床研究报告中将报告本研究结果，且包含所有参与本研究的临床研究中心的病例报告表数据。将根据入组表现或与研究相关的技能，及相关专业知识确定协调研究人员。在临床研究报告发布后得到的分析结果，将写入单独的报告中，无需对临床研究报告进行修改。在出版结果时，不会公开受试者的身份信息。所有与研究相关的著作以及受到版权保护的数据（除外下文所述的研究者发表的任何文献），将是申办方的财产，并且作为作者和版权所有者。

如果研究人员希望发表该研究的相关信息，须在投稿或展示前至少 60 天向申

办方提供文稿复件，供申办方审查。对于摘要、会议海报或其它材料（如邀请讲演或客座讲演等），可安排快速审查。在申办方书面要求下，研究人员需要保留最多额外 60 天，以允许完成专利申请。如果出现科学完整性或管理依从性方面的问题，则申办方将与研究人员审查这些问题。申办方不得强制要求对科学内容进行修改，并且无权删除信息。

11. 参考文献

［1］ 徐建明. 从左右半结肠癌的生物学差异谈结直肠癌[J]. 中华肿瘤杂志, 2016, 38(5):397-400.

［2］ Peto J．Cancer epidemiology in the last century and the next decade.［J］Nature, 2001,411 (6835)：390 -395．

［3］ 蔡琳，Yang B，Parkin D M，等. 亚太若干地区恶性肿瘤流行趋势分析［J］. 肿瘤，2004，24(5) : 422-426.

［4］ 董志伟，乔友林，李连弟，等. 中国癌症高发现场报告[J]. 中国肿瘤，2009，18(1): 429.

［5］ Golfinopoulos V, Salant IG, Pavlidis N, et al. Survival and disease progression benefits with treatment regimens for advanced colorectal cancer: a meta analysis [J]. Lancet Oncol, 2007, 8 (10) : 8982911.

［6］ Porebska I,Harlozinska A,Bojarowski T．Expression of the tyrosine kinase activity growth factor receptors (EGFR,ERB-B2,ERB-B3)in colorectal adenocarcinomas and adenomas [J]．Tumour Biol 2000．21：105-ll5．

［7］ Harris M. Monoclonal antibodies as therapeutic agents for cancer [J].Lancet Oncol, 2004,5 (5) :292-302.

［8］ CereaG, RicottaR, SchiavettoI, et al.Cetuximab for treatment of metastatic colorectal cancer [J]. Ann Oncol, 2006, 17 (Supp l7) : vii66-vii67.

［9］ D.Arnold, B.Lueza, J.-Y. Douilard, et al. Prognostic and predictive value of primary tumor side in patients with RAS wild-type metastatic colorectal cancer treated with chemotherapy and EGFR directed antibodies in six randomized trials. Annals of Oncology 0: 1-17, 2017

［10］Roberto Moretto, Chiara Cremolini, Daniele Rossini, et al. Location of primary tumor and benefit from anti-epidermal growth factor receptor monoclonal antibodies in patients with RAS and BRAF wild-type metastatic colorectol cancer. Oncologist 2016 Aug: 21(8): 988-994.

［11］Neuenschwander B, Branson M, Gsponer T (2008) Critical aspects of the Bayesian approach to phase I cancer trials. Statist. Med, 2008; 27: 2420-2439.

12. 附件

附件一：ECOG 评分

活动状态	描述
**0**	无症状，完全主动活动，及能够进行无限制的患病前活动
**1**	有症状，完全能行走，但重体力活动受限，能从事轻的或以坐为主的工作，如轻微家务，办公室工作
**2**	有症状，能行走，生活可自理，但不能进行任何的体力活动，约有 50% 以上的时间清醒：即白天卧床时间〈50%
**3**	有症状，有限的生活自理能力，清醒时间卧床或坐椅〉50%，但尚未卧床不起
**4**	完全失去功能，生活完全不能自理，卧床不起
**5**	死亡

附件二：输液反应处理推荐

1 级输液反应：

- 降低输液速度 50%；
- 观察患者症状体征是否恶化；
- 在随后 SCT200 输液治疗前，肌肉注射盐酸苯海拉明 40mg（或同等药物）；其他预防性用药由研究者决定。

2 级输液反应：

- 降低输液速度 50%或暂停输液；
- 肌肉注射盐酸苯海拉明 40mg （或同等药物），如有发热口服 650mg 对乙酰氨基酚，同时给氧；
- 暂停输液后，一旦输液反应消失或降低至 1 级，则以之前 50%的输液速度继续静脉输液；
- 观察患者症状体征是否恶化；
- 在随后 SCT200 输液治疗前，肌肉注射盐酸苯海拉明 40mg（或同等药物）；其他预防性用药由研究者决定。

对于第二次出现的 1 级或 2 级输液反应，静脉给予 8-10mg 地塞米松（或同等药物）；然后，在随后输液前，预防性地给予苯海拉明 40mg（或同等药物）、口服对乙酰氨基酚，和静脉 8-10mg 地塞米松（或同等药物）。

3 级输液反应：

- 停止输液，去除输液导管；
- 肌肉注射 40mg 盐酸苯海拉明（或同等药物）、静脉给予地塞米松 8-10mg、如有支气管痉挛给予气管扩张剂，和其他对症治疗；
- 停止 SCT200 治疗。

4 级输液反应

- 停止输液，去除输液导管；

- 肌肉注射 40mg 盐酸苯海拉明（或同等药物）、静脉给予地塞米松 8-10mg，和其他对症治疗；
- 如有临床指征给予肾上腺素或气管扩张剂；
- 如有临床指征住院观察；
- 停止 SCT200 治疗。

附件三：皮肤毒性反应处理推荐

常规皮肤护理：出现皮疹后，忌食辛辣、刺激性食物，保证睡眠。穿着宽松、舒适、非化学合成材料的衣裤、鞋袜，避免皮肤损伤。保持皮肤清洁，避免接触碱性洗涤用品。在皮肤干燥部位涂抹不含酒精的润肤霜。尽量减少日晒时间，皮肤暴露部位涂 SPF>15 的防晒用品，在日晒前 1-2h 使用，可根据日晒持续时间重复使用。

处理原则：出现 I/II 级皮肤毒， 除加强皮肤护理外，研究者认为必要可局部使用复方醋酸地塞米松软膏(皮炎平)、氢化可的松软膏(1%或 2.5%)或氯林可霉素凝胶(10%)，红霉素软膏及磺胺嘧啶银乳膏。对皮肤干燥伴瘙痒者，可予薄酚甘油洗剂(每天 2 次)或苯海拉明软膏涂抹瘙痒局部。

出现 III 级皮肤毒时，需关注皮肤发炎和感染情况，一旦出现严重的皮肤感染，研究者认为必要时可选择采用局部或口服抗生素， 或行静脉给予抗生素治疗，或遵循皮肤科专家意见，但不推荐局部使用皮质类固醇治疗。

附件四：实体肿瘤的疗效评价

实体肿瘤的疗效评价标准

(按照 RECIST1.1 版本)

完全缓解**(CR):**自基线期评估后，所有可见病灶消失(淋巴结短径<10mm)；

部分缓解**(PR):**各目标病灶最大径之总和较基线的总和缩小≥30%，无新病灶出现；

稳定病情**(SD):**各目标病灶最大径之总和的变化界于 PR 和 PD 之间；

病情进展**(PD):**各目标病灶最大径之总和较原目标病灶(包括基线)的总和增加至少超过 20%，且目标病灶长径之总和绝对值增加至少 5mm，或出现新病灶。

可测量病灶：根据 RECIST1.1 标准，采用临床或影像学方法可以测量直径的病灶。如肺内病灶 X 胸片≥ 20 mm，普通 CT 或 MRI 扫描≥ 10 mm，螺旋 CT 扫描直径≥ 10 mm, 淋巴结短径≥15mm。

不可测量的病灶：包括单径可测病灶、边界不清的肿块、细小病灶无法测径肿瘤(如肺内粟粒或点片状病灶)、双径均小于 10 mm 的病灶、直径小于扫描间距的肿块等。不可测量的病灶也包括不可评价病灶，如成骨性转移、胸水、腹水、心包积液、炎性乳腺癌、皮肤或肺内癌性淋巴管炎，软脑膜病灶，囊性病变、成像技术不能重现的腹部肿块或包块等以及过去曾经放疗或局部治疗过的病灶且无进展者。

说明：

1、目标病灶(靶病灶)为可测量病灶。根据病灶最长径大小、能否易于准确重复测量评价等来确定目标病灶。

2、总计最多记录5个目标病灶。如可测量病灶涉及有多个器官，每个所涉及的器官应选择不多于2个目标病灶作为评价对象。其余可作为非目标病灶。

3、新病灶的出现必须是明确的。应排除因影像学技术的不同，成像形态的改变，或者肿瘤以外的其它病变(如：原病灶出现部分或完全缓解的复发，或肝脏病灶的坏死可能在CT报告上定为新的囊性病变等)以及非常小的不明确的新病灶。必要时需

要进行4周进一步评价以补充确认其是否是新的病灶。

非目标病灶的评估：

	非目标病灶
CR	自基线期后，非目标病灶全部消失
非 CR/非 PD	有1 个或多个非目标病灶存在，“稳定或减少”
PD	已存在的可测量病灶出现整体恶化的明确性进展或出现一个或多个新病灶#

#新病灶的指征必须非常明确

非目标病灶明确进展的定义，必须满足可测量的非目标病灶整体的恶化程度已达到必须终止治疗的程度。而一个或多个非目标病灶尺寸的一般性增大往往不足以达到进展标准，因此，在目标病灶为稳定或部分缓解时，仅依靠非目标病灶的改变就定义整体肿瘤进展的情况几乎是十分稀少的。

总体疗效评价：

目标病灶	非目标病灶	新病灶*	整体疗效评价
CR	CR	无	CR
CR	非CR/非PD或不能评估	无	PR
PR	非PD或不能评估	无	PR
SD	非 PD 或不能评估	无	SD
PD	任何情况	有或无	PD
任何情况	任何情况	有	PD

*新病灶的指征必须非常明确
